# Predicting atrial fibrillation in patients with acute respiratory failure using machine learning: application of the MIMIC-III and MIMIC-IV datasets

**DOI:** 10.3389/fcvm.2025.1696609

**Published:** 2025-10-09

**Authors:** Rixuan Li

**Affiliations:** Jinzhou Medical University, Jinzhou, China

**Keywords:** machine learning, predictive model, atrial fibrillation, acute respiratory failure, MIMIC- IV database

## Abstract

**Background:**

Acute respiratory failure (ARF) and atrial fibrillation (AF) are common diseases. This study established a predictive model for the risk of atrial fibrillation in patients with ARF, aiming to provide tools for clinical application.

**Methods:**

This study examined the data of 21,594 patients in the MIMIC-IV database, including factors such as age, vital signs, and laboratory results on the first day of admission. Six feature selection techniques and six machine learning algorithms were used to construct the prediction model, and then the prediction model was verified using the MIMIC-III database. Evaluate the performance of the model through the comparison of results.

**Results:**

A total of 59 predictor variables were identified, among which age was the most important factor. These variables are used to establish predictive models. The verification results show that the XGBoost model (AUC: 0.816) and the Random Forest (RF) model (AUC: 0.822) have the best performance. This study presents the first predictive model for atrial fibrillation in patients with acute respiratory failure.

**Conclusions:**

Both the XGBoost and RF models demonstrated outstanding performance. These findings will make significant contributions to the diagnosis of clinical complications and the resolution of public health issues.

## Introduction

Respiratory failure is a respiratory disorder characterized by hypoxemia or hypercapnia (1). Atrial fibrillation (AF) is the most prevalent type of persistent arrhythmia in the circulatory system. The incidence of AF in patients with respiratory failure is between 10% and 15%, and it can reach as high as 50% in patients with type 2 respiratory failure (2, 3). Factors such as atrial fibrosis, aging, hypertension, obesity, diabetes, and genetic predisposition play a significant role in triggering AF (4). Bemis et al. propose that hypoxemia and hypercapnia are risk factors for AF. In hypercapnic patients, factors such as intrathoracic pressure, autonomic nervous system fluctuations, atrial stretching, and remodeling play a role in the development of AF. Furthermore, elevated pulmonary artery pressure in respiratory failure patients leads to right ventricular hypertension and right atrial enlargement, which in turn triggers AF (5). AF can result in serious complications, such as stroke, coronary heart disease, cognitive impairment, and even dementia, suggesting that respiratory failure may trigger AF by impacting the pulmonary veins (6–8).

Schüttler et al. simulated human atrial fibrillation using modeling techniques (9). Roussos et al. highlighted that patients with respiratory failure often experience neural suppression and neuromuscular conduction disorders, which may be indicative of neurotransmitter disturbances associated with AF and myocardial dysfunction (10). However, the mechanisms by which respiratory failure induces AF remain poorly understood.

Although the diagnosis of respiratory failure and AF is well-established (11–13), two-thirds of AF patients report shortness of breath, making it difficult to promptly identify AF in patients with respiratory failure (14). Consequently, developing an early prediction model to prevent or predict AF in these patients is crucial for improving clinical outcomes.

Machine learning has become a powerful tool in medicine, driving significant progress across various medical disciplines (15). Previous AF prediction models have focused on ICU patients (16) and are limited by cohort heterogeneity, which can reduce accuracy and generalizability. This study includes both ICU and non-ICU patients but targets a single disease, minimizing heterogeneity and improving the model's sensitivity and applicability.To date, no predictive model exists for forecasting acute respiratory failure in patients with pneumonia. This study aims to fill this critical research gap by developing a machine learning-based model for early detection of AF in patients with acute respiratory failure. The main objective of this model is to help healthcare professionals proactively prevent or detect AF early, facilitating personalized treatment plans, improving patient outcomes, reducing healthcare costs, and potentially saving lives.

To date, predictive models specifically designed to identify atrial fibrillation (AF) in patients with acute respiratory failure remain scarce. Existing research has predominantly focused on general AF risk factors or populations with cardiovascular disease, leaving a critical research gap in this high-risk cohort. To our knowledge, this study represents the first development and validation of a machine learning model specifically designed to predict AF in this distinct population. By integrating comprehensive clinical, laboratory, and physiological variables, our model demonstrates robust predictive performance, offering an innovative and clinically actionable tool for early risk stratification in patients with acute respiratory failure.

This study utilizes the MIMIC-Ⅳ and MIMIC-Ⅲ databases alongside machine learning techniques to create a predictive model for the development of AF in patients with acute respiratory failure.

## Materials and methods

### Study population

This retrospective observational study extracted data from patients diagnosed with acute respiratory failure (ARF) using the MIMIC-IV (version3.1) and MIMIC-III (version1.4) databases. At present, there are no international guidelines for diagnosing respiratory failure. However, as mentioned earlier, the diagnosis of respiratory failure and atrial fibrillation (AF) is widely recognized. The main purpose of this study is to rule out the diagnosis of respiratory failure before AF, thereby laying the foundation for further research.

The diagnostic criteria for acute respiratory failure (ARF) are as follows: Type I ARF is characterized by arterial oxygen partial pressure (PaO₂) ≤60 mmHg and arterial carbon dioxide partial pressure (PaCO₂) ≥45 mmHg, while Type II ARF is characterized by PaCO₂ ≥45 mmHg. In the MIMIC database, it is defined by specific ICD-10 and ICD-9 codes (e.g., 51881, J95821).

Diagnostic criteria for atrial fibrillation (AF) include a history of diagnosed or undiagnosed AF, an electrocardiogram (ECG) upon admission showing arrhythmia, irregular heartbeats, and absent P waves, as well as a history of AF. In leads II, III, and aVF, fibrillation waves replace P waves. Additionally, rapid ventricular response (RVR) or a normal ventricular rate may be present, particularly in patients with chronic atrial fibrillation.

By identifying the diagnosis through specific label (seq_num) in the MIMIC database, the sequence of events in the patient's illness is determined, thereby establishing the occurrence of atrial fibrillation following acute respiratory failure. Furthermore, based on the label (seq_num) and diagnosis, all patients were free of atrial fibrillation upon admission.

### Data collection

In this retrospective study, patient information was obtained from the MIMIC-IV (version 3.1) (17) and MIMIC-III (version 1.4) (18) databases. Extract the following data points for analysis:

### Demographic information

Specific demographic details of the patient, including age, gender and other identifying characteristics.

### Vital signs and laboratory results on the first day of admission

Vital signs: body temperature, respiratory rate, systolic blood pressure, diastolic blood pressure, mean arterial pressure, weight.

Laboratory inspection White blood cell count, hemoglobin, platelet count, urea nitrogen, serum creatinine, blood glucose, serum sodium, serum chlorine, serum potassium, hematocrit, eosinophils, basophils, neutrophils, monocytes, lymphocytes, serum calcium, international normalized ratio, prothrombin time, activated partial thromboplastin time, bicarbonate concentration, anion gap Alanine aminotransferase, aspartate aminotransferase, alkaline phosphatase, bilirubin.

**Scores and Indices:**

Glasgow Coma Scale (GCS)

Simplified Acute Physiology Score II (SAPS II)

Acute Physiology Score III (APS III)

Sequential Organ Failure Assessment (SOFA)

Oxford Acute Severity of Illness Score (OASIS)

**Comorbidities:**

Congestive heart failure

Cerebrovascular disease

Liver disease

Kidney disease

AIDS

Chronic lung disease

Diabetes

Paralysis

Malignancies

Metastatic solid tumors

Peptic ulcer

Dementia

Rheumatic diseases

**Blood Gas Analysis:**

Partial pressure of oxygen (PaO2)

Partial pressure of carbon dioxide (PaCO2)

Oxygen saturation

Lactate

pH

Base excess

Total carbon dioxide (TCO2)

**Other Variables:**

Mechanical ventilation status

Oxygen flow rate

Whether elective surgery was performed

Length of hospitalization prior to ICU admission

### Handling missing data

Variables with missing values exceeding 20% will be excluded from the analysis. For variables with missing values of 20% or less, multiple substitutions will be applied using the “mice” package in R (version 4.5.1).Missing values in the dataset were handled using multiple imputation methods from the mice package in R. Specifically, five imputed datasets (m = 5) were generated via the random forest method (method = “rf”), which preserves nonlinear relationships and interactions between variables. The second imputed dataset was selected as the representative sample for subsequent analyses [“complete(imputed_data, 2)”], while the consistency of results across all imputed datasets was verified.

This method ensures thorough data processing while adhering to best practices for handling missing data in clinical research.

### Statistical analysis

Continuous variables will be summarized using the median and interquartile range (IQR). The normality of continuous variables was evaluated using the Kolmogorov–Smirnov (K-S) test, and the rank sum test was used for inter-group comparisons. Categorical variables will be expressed in terms of frequency and percentage, and chi-square tests will be used for inter-group comparisons. The MIMIC-IV dataset will be divided into a training set and an internal validation set in a ratio of 4:1 (or 8:2), while the MIMIC-III dataset will serve as the external validation set. This study did not employ K-fold cross-validation or bootstrapping methods, but no statistically significant differences were observed between the internal training set and the internal validation set.

Feature selection will employ a variety of methods, including LASSO, MDA, MDG, FS, BS and BE. The intersection of the selected variables will be used to develop models for XGBoost, random forest, logistic regression, decision tree, support vector machine and artificial neural network.

The performance of the model was evaluated by using ROC curve, calibration curve, decision curve analysis, f1 score, sensitivity, specificity, PLR, NLR, PPV, NPV and cutoff analysis. The model with the highest ROC performance was selected as the main model. SHAP analysis will be used to explain the final model.

All statistical analyses were conducted using R software (version 4.5.1), *p* < 0.05 is considered statistically significant.

### Ethics approval and consent to participate

The Collaborative Institution Training Program (CITI) certification has been completed, meeting the database access requirements. All the databases used in this study contain de-identified data, so patient consent is not required. All procedures comply with relevant guidelines and regulations.

## Result

**Figure 1:**
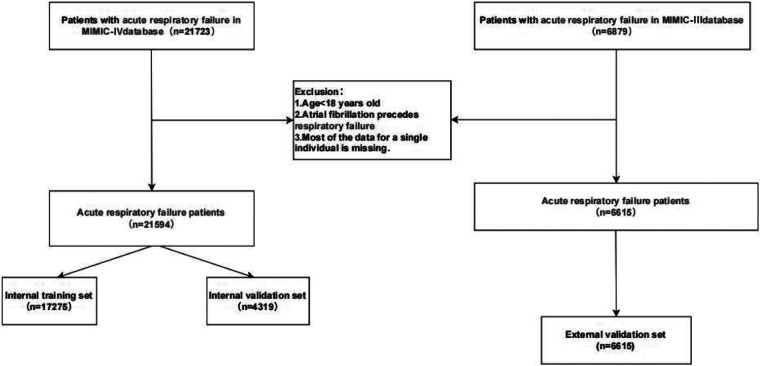
Flow chart.

A total of 21,723 patients with acute respiratory failure were extracted from the MIMIC-IV (v3.1) database. After excluding patients under 18 years old and those diagnosed with atrial fibrillation before the onset of acute respiratory failure, the final cohort included 21,594 patients diagnosed with acute respiratory failure, as shown in Figure 1.

Patients in the MIMIC-IV database were divided into two groups based on the presence of atrial fibrillation. Tables 1, 2 presents a comparison of baseline characteristics between the atrial fibrillation group and the non-atrial fibrillation group.

**Table 1:** Internal set.

Characteristic	Non-AF(*n* = 14,372)	AF(*n* = 7,222)	*P*
Age, M (Q_1_, Q_3_)	64.30 (52.98, 75.09)	76.17 (67.58, 84.06)	<.001
ABG			
Po2, M (Q_1_, Q_3_)	79.00 (50.00, 140.00)	77.00 (47.00, 142.00)	0.031
Pco2, M (Q_1_, Q_3_)	43.00 (36.00, 52.00)	44.00 (37.00, 53.00)	<.001
Ph, M (Q_1_, Q_3_)	7.36 (7.29, 7.42)	7.36 (7.29, 7.42)	<.001
Baseexcess, M (Q_1_, Q_3_)	0.00 (−4.00, 2.00)	0.00 (−4.00, 2.00)	<.001
Totalco2, M (Q_1_, Q_3_)	25.00 (21.00, 29.00)	26.00 (22.00, 30.00)	<.001
Lactate, M (Q_1_, Q_3_)	1.70 (1.20, 2.70)	1.70 (1.20, 2.60)	0.549
Spo2, M (Q_1_, Q_3_)	96.78 (95.00, 98.42)	96.64 (94.95, 98.21)	<.001
Lab			
Hematocrit, M (Q_1_, Q_3_)	34.50 (29.60, 39.70)	33.50 (29.10, 38.40)	<.001
Hemoglobin, M (Q_1_, Q_3_)	11.10 (9.40, 12.90)	10.60 (9.10, 12.30)	<.001
Platelets, M (Q_1_, Q_3_)	223.00 (158.00, 300.00)	211.00 (152.00, 286.00)	<.001
Wbc, M (Q_1_, Q_3_)	13.10 (9.30, 18.40)	13.30 (9.50, 18.60)	0.011
Aniongap, M (Q_1_, Q_3_)	16.00 (13.00, 19.00)	16.00 (14.00, 19.00)	0.034
Bicarbonate, M (Q_1_, Q_3_)	24.00 (21.00, 27.00)	25.00 (22.00, 28.00)	<.001
Bun, M (Q_1_, Q_3_)	23.00 (15.00, 39.00)	32.00 (21.00, 51.00)	<.001
Calcium, M (Q_1_, Q_3_)	8.70 (8.20, 9.10)	8.60 (8.20, 9.10)	0.440
Chloride, M (Q_1_, Q_3_)	105.00 (100.00, 109.00)	104.00 (99.00, 108.00)	<.001
Creatinine, M (Q_1_, Q_3_)	1.10 (0.80, 1.90)	1.40 (1.00, 2.30)	<.001
Glucose, M (Q_1_, Q_3_)	154.00 (122.00, 209.00)	156.00 (125.00, 209.00)	0.058
Sodium, M (Q_1_, Q_3_)	140.00 (137.00, 143.00)	140.00 (137.00, 143.00)	0.276
Potassium, M (Q_1_, Q_3_)	4.50 (4.10, 5.10)	4.60 (4.20, 5.20)	<.001
Abs Basophils, M (Q_1_, Q_3_)	0.02 (0.00, 0.05)	0.02 (0.00, 0.04)	0.021
Abs Eosinophils, M (Q_1_, Q_3_)	0.03 (0.00, 0.12)	0.03 (0.00, 0.11)	0.004
Abs Lymphocytes, M (Q_1_, Q_3_)	1.09 (0.66, 1.72)	0.98 (0.59, 1.56)	<.001
Abs Monocytes, M (Q_1_, Q_3_)	0.64 (0.37, 0.99)	0.67 (0.40, 1.03)	<.001
Abs Neutrophils, M (Q_1_, Q_3_)	9.73 (6.37, 14.27)	9.95 (6.71, 14.45)	<.001
Inr, M (Q_1_, Q_3_)	1.30 (1.10, 1.60)	1.50 (1.20, 2.10)	<.001
Pt, M (Q_1_, Q_3_)	14.00 (12.40, 17.10)	16.40 (13.70, 23.20)	<.001
Ppt, M (Q_1_, Q_3_)	32.00 (28.00, 42.70)	35.70 (30.00, 52.90)	<.001
Alt, M (Q_1_, Q_3_)	28.00 (17.00, 62.00)	26.00 (16.00, 60.00)	<.001
Alp, M (Q_1_, Q_3_)	91.00 (67.00, 133.00)	93.00 (68.00, 134.00)	0.008
Ast, M (Q_1_, Q_3_)	42.00 (24.00, 92.00)	39.00 (24.00, 88.00)	0.006
Bilirubin Total, M (Q_1_, Q_3_)	0.60 (0.40, 1.10)	0.70 (0.40, 1.10)	<.001
Vital Signs			
Sbp, M (Q_1_, Q_3_)	114.90 (105.35, 127.13)	111.70 (103.67, 122.61)	<.001
Dbp, M (Q_1_, Q_3_)	63.12 (56.55, 70.88)	60.95 (54.77, 68.16)	<.001
Mbp, M (Q_1_, Q_3_)	77.52 (71.19, 85.46)	75.37 (69.68, 82.37)	<.001
Resp Rate, M (Q_1_, Q_3_)	20.17 (17.56, 23.30)	20.40 (17.91, 23.36)	<.001
Temperature, M (Q_1_, Q_3_)	36.89 (36.64, 37.22)	36.80 (36.56, 37.09)	<.001
Weight, M (Q_1_, Q_3_)	78.00 (64.60, 95.30)	79.80 (65.60, 96.80)	<.001
Scores			
Gcs, M (Q_1_, Q_3_)	15.00 (15.00, 15.00)	15.00 (15.00, 15.00)	<.001
Gcs Motor, M (Q_1_, Q_3_)	6.00 (4.00, 6.00)	6.00 (4.00, 6.00)	<.001
Gcs Verbal, M (Q_1_, Q_3_)	1.00 (0.00, 5.00)	4.00 (0.00, 5.00)	<.001
Gcs Eyes, *n*(%)			<.001
1	4,190 (29.15)	1,969 (27.26)	
2	1,238 (8.61)	554 (7.67)	
3	1,907 (13.27)	952 (13.18)	
4	7,037 (48.96)	3,747 (51.88)	
Gcs Unable, *n*(%)			<.001
0	7,778 (54.12)	4,303 (59.58)	
1	6,594 (45.88)	2,919 (40.42)	
Oasis, M (Q_1_, Q_3_)	34.00 (28.00, 40.00)	36.00 (30.00, 43.00)	<.001
Oasis Prob, M (Q_1_, Q_3_)	0.14 (0.07, 0.25)	0.17 (0.09, 0.33)	<.001
Sofa, M (Q_1_, Q_3_)	5.00 (3.00, 8.00)	6.00 (3.25, 9.00)	<.001
Urineoutput, M (Q_1_, Q_3_)	1,380.00 (770.00, 2,215.00)	1,185.00 (645.00, 2,000.00)	<.001
Apsiii, M (Q_1_, Q_3_)	46.00 (34.00, 63.00)	52.00 (40.00, 68.00)	<.001
Sapsii, M (Q_1_, Q_3_)	37.00 (28.00, 48.00)	43.00 (35.00, 53.00)	<.001
Sapsii Prob, M (Q_1_, Q_3_)	0.20 (0.09, 0.41)	0.31 (0.17, 0.53)	<.001
Respiration, *n*(%)			<.001
0	2,514 (17.49)	1,047 (14.50)	
1	372 (2.59)	168 (2.33)	
2	6,281 (43.70)	3,462 (47.94)	
3	2,661 (18.52)	1,228 (17.00)	
4	2,544 (17.70)	1,317 (18.24)	
Coagulation, *n*(%)			<.001
0	9,388 (65.32)	4,501 (62.32)	
1	2,536 (17.65)	1,514 (20.96)	
2	1,511 (10.51)	873 (12.09)	
3	716 (4.98)	247 (3.42)	
4	221 (1.54)	87 (1.20)	
Liver, *n*(%)			<.001
0	11,158 (77.64)	5,466 (75.69)	
1	1,311 (9.12)	919 (12.73)	
2	1,195 (8.31)	626 (8.67)	
3	374 (2.60)	135 (1.87)	
4	334 (2.32)	76 (1.05)	
Cardiovascular, *n* (%)			<.001
0	2,661 (18.52)	825 (11.42)	
1	7,857 (54.67)	4,065 (56.29)	
2	43 (0.30)	55 (0.76)	
3	837 (5.82)	596 (8.25)	
4	2,974 (20.69)	1,681 (23.28)	
Cns, *n*(%)			<.001
0	8,900 (61.93)	4,161 (57.62)	
1	2,738 (19.05)	1,626 (22.51)	
2	1,016 (7.07)	572 (7.92)	
3	1,024 (7.12)	535 (7.41)	
4	694 (4.83)	328 (4.54)	
Renal, *n*(%)			<.001
0	6,981 (48.57)	2,449 (33.91)	
1	3,060 (21.29)	1,933 (26.77)	
2	1,418 (9.87)	1,049 (14.53)	
3	1,456 (10.13)	951 (13.17)	
4	1,457 (10.14)	840 (11.63)	
Charlson			
Myocardial Infarct, *n*(%)			<.001
0	12,069 (83.98)	5,444 (75.38)	
1	2,303 (16.02)	1,778 (24.62)	
Congestive Heart Failure, *n*(%)			<.001
0	10,088 (70.19)	2,786 (38.58)	
1	4,284 (29.81)	4,436 (61.42)	
Peripheral Vascular Disease, *n*(%)			<.001
0	13,041 (90.74)	6,117 (84.70)	
1	1,331 (9.26)	1,105 (15.30)	
Cerebrovascular Disease, *n*(%)			<.001
0	12,244 (85.19)	5,948 (82.36)	
1	2,128 (14.81)	1,274 (17.64)	
Dementia, *n*(%)			<.001
0	13,660 (95.05)	6,654 (92.14)	
1	712 (4.95)	568 (7.86)	
Chronic Pulmonary Disease, *n*(%)			<.001
0	9,365 (65.16)	4,480 (62.03)	
1	5,007 (34.84)	2,742 (37.97)	
Rheumatic Disease, *n*(%)			0.043
0	13,879 (96.57)	6,935 (96.03)	
1	493 (3.43)	287 (3.97)	
Peptic Ulcer Disease, *n*(%)			0.282
0	13,981 (97.28)	7,007 (97.02)	
1	391 (2.72)	215 (2.98)	
Mild Liver Disease, *n*(%)			<.001
0	12,117 (84.31)	6,412 (88.78)	
1	2,255 (15.69)	810 (11.22)	
Diabetes Without Cc, *n*(%)			<.001
0	10,935 (76.09)	5,323 (73.71)	
1	3,437 (23.91)	1,899 (26.29)	
Diabetes With Cc, *n*(%)			<.001
0	12,512 (87.06)	5,937 (82.21)	
1	1,860 (12.94)	1,285 (17.79)	
Paraplegia, *n*(%)			0.074
0	13,453 (93.61)	6,805 (94.23)	
1	919 (6.39)	417 (5.77)	
Renal Disease, *n*(%)		<.001	
0	11,174 (77.75)	4,422 (61.23)	
1	3,198 (22.25)	2,800 (38.77)	
Malignant Cancer, *n*(%)			<.001
0	11,983 (83.38)	6,152 (85.18)	
1	2,389 (16.62)	1,070 (14.82)	
Severe Liver Disease, *n*(%)			<.001
0	13,187 (91.75)	6,913 (95.72)	
1	1,185 (8.25)	309 (4.28)	
Metastatic Solid Tumor, *n*(%)			<.001
0	13,183 (91.73)	6,783 (93.92)	
1	1,189 (8.27)	439 (6.08)	
Aids, *n*(%)			<.001
0	14,198 (98.79)	7,208 (99.81)	
1	174 (1.21)	14 (0.19)	
Intervention/Status			
Preiculos, M (Q_1_, Q_3_)	106.00 (47.00, 1,218.51)	125.00 (51.00, 2,338.21)	<.001
O2 Flow, M (Q_1_, Q_3_)	8.00 (4.00, 10.00)	6.00 (3.00, 10.00)	0.012
Nsaid, *n*(%)			<.001
0	8,331 (57.97)	3,227 (44.68)	
1	6,041 (42.03)	3,995 (55.32)	
Mechvent, *n*(%)			<.001
0	6,105 (42.48)	3,424 (47.41)	
1	8,267 (57.52)	3,798 (52.59)	
Electivesurgery, n(%)			<.001
0	14,294 (99.46)	7,094 (98.23)	
1	78 (0.54)	128 (1.77)	
Ventilation Status, n(%)			<.001
0	745 (5.18)	325 (4.50)	
1	4,880 (33.95)	2,872 (39.77)	
2	480 (3.34)	271 (3.75)	
3	406 (2.82)	237 (3.28)	
4	7,861 (54.70)	3,517 (48.70)	

**Table 2:** External set.

Variables	Non-AF (*n* = 4,274)	AF (*n* = 1,802)	*P*
Age, M (Q_1_, Q_3_)	61.37 (49.39, 74.51)	75.55 (65.44, 83.06)	<.001
ABG			
Po2, M (Q_1_, Q_3_)	100.00 (69.00, 189.00)	101.00 (68.00, 182.00)	0.740
Lactate, M (Q_1_, Q_3_)	2.10 (1.40, 3.70)	2.10 (1.40, 3.40)	0.081
Totalco2, M (Q_1_, Q_3_)	25.00 (21.00, 29.00)	26.00 (22.00, 31.00)	<.001
Spo2, M (Q_1_, Q_3_)	97.38 (95.67, 98.74)	97.33 (95.74, 98.65)	0.528
LAB			
Hematocrit, M (Q_1_, Q_3_)	34.70 (30.60, 39.40)	34.00 (30.33, 38.30)	0.002
Hemoglobin, M (Q_1_, Q_3_)	11.50 (10.10, 13.20)	11.20 (9.90, 12.70)	<.001
Platelets, M (Q_1_, Q_3_)	235.00 (159.25, 321.00)	232.00 (170.00, 313.00)	0.406
Wbc, M (Q_1_, Q_3_)	13.00 (9.00, 18.20)	13.20 (9.30, 18.20)	0.260
Aniongap, M (Q_1_, Q_3_)	16.00 (14.00, 19.00)	16.00 (14.00, 19.00)	0.352
Bicarbonate,M (Q_1_, Q_3_)	22.00 (18.00, 25.00)	23.00 (19.00, 26.00)	<.001
Bun, M (Q_1_, Q_3_)	24.00 (16.00, 41.00)	32.00 (21.00, 50.00)	<.001
Chloride, M (Q_1_, Q_3_)	107.00 (103.00, 111.00)	106.00 (102.00, 111.00)	<.001
Creatinine, M (Q_1_, Q_3_)	1.10 (0.80, 2.00)	1.40 (0.90, 2.20)	<.001
Glucose, M (Q_1_, Q_3_)	159.00 (126.00, 214.00)	162.00 (128.00, 211.00)	0.346
Sodium, M (Q_1_, Q_3_)	141.00 (138.00, 143.00)	141.00 (138.00, 143.75)	0.450
Potassium, M (Q_1_, Q_3_)	4.50 (4.10, 5.10)	4.60 (4.10, 5.20)	<.001
Abs Eosinophils, M (Q_1_, Q_3_)	0.08 (0.01, 0.23)	0.08 (0.01, 0.24)	0.256
Abs Lymphocytes, M (Q_1_, Q_3_)	1.31 (0.71, 2.27)	1.23 (0.68, 2.07)	0.006
Abs Neutrophils, M (Q_1_, Q_3_)	0.13 (0.09, 0.18)	0.13 (0.09, 0.18)	0.260
Inr, M (Q_1_, Q_3_)	1.30 (1.10, 1.60)	1.50 (1.20, 2.20)	<.001
Pt, M (Q_1_, Q_3_)	14.40 (13.20, 16.90)	15.85 (13.90, 21.30)	<.001
Ppt, M (Q_1_, Q_3_)	32.60 (27.50, 44.48)	35.40 (29.20, 49.30)	<.001
Bilirubin Total, M (Q_1_, Q_3_)	0.60 (0.40, 1.20)	0.70 (0.40, 1.20)	0.051
Vital signs			
Sbp, M (Q_1_, Q_3_)	115.22 (105.07, 128.58)	113.00 (104.51, 124.99)	<.001
Dbp, M (Q_1_, Q_3_)	60.38 (53.68, 67.88)	57.76 (51.33, 64.75)	<.001
Mbp, M (Q_1_, Q_3_)	76.83 (69.85, 85.03)	73.78 (67.83, 81.28)	<.001
Resp Rate, M (Q_1_, Q_3_)	19.74 (16.87, 23.33)	19.90 (17.25, 22.85)	0.398
Temperature, M (Q_1_, Q_3_)	36.90 (36.45, 37.39)	36.73 (36.32, 37.20)	<.001
Scores			
Gcs, M (Q_1_, Q_3_)	15.00 (14.00, 15.00)	15.00 (13.00, 15.00)	0.099
Gcsmotor, M (Q_1_, Q_3_)	6.00 (4.00, 6.00)	6.00 (5.00, 6.00)	0.057
Gcsverbal, M (Q_1_, Q_3_)	1.00 (0.00, 5.00)	1.00 (0.00, 5.00)	0.002
Gcs Eyes, *n*(%)		0.087	
1	1,076 (25.18)	409 (22.70)	
2	472 (11.04)	188 (10.43)	
3	1,111 (25.99)	470 (26.08)	
4	1,615 (37.79)	735 (40.79)	
Oasis, M (Q_1_, Q_3_)	36.00 (30.00, 43.00)	38.00 (32.00, 44.00)	<.001
Oasis Prob, M (Q_1_, Q_3_)	0.17 (0.09, 0.33)	0.21 (0.11, 0.36)	<.001
Sofa, M (Q_1_, Q_3_)	5.00 (3.00, 8.00)	5.00 (3.00, 8.00)	0.449
Urineoutput, M (Q_1_, Q_3_)	1,530.00 (870.00, 2,425.00)	1,310.00 (740.00, 2,058.75)	<.001
Apsiii, M (Q_1_, Q_3_)	49.00 (35.00, 66.00)	53.00 (40.00, 68.00)	<.001
Apsiii Prob, M (Q_1_, Q_3_)	0.11 (0.06, 0.21)	0.13 (0.07, 0.23)	<.001
Sapsii, M (Q_1_, Q_3_)	40.00 (29.00, 51.00)	44.00 (36.00, 54.00)	<.001
Sapsii Prob, M (Q_1_, Q_3_)	0.25 (0.10, 0.48)	0.33 (0.18, 0.55)	<.001
Respiration, *n*(%)			<.001
0	1,511 (35.35)	681 (37.79)	
1	181 (4.23)	75 (4.16)	
2	933 (21.83)	429 (23.81)	
3	1,090 (25.50)	461 (25.58)	
4	559 (13.08)	156 (8.66)	
Coagulation, *n* (%)			<.001
0	2,881 (67.41)	1,222 (67.81)	
1	599 (14.01)	345 (19.15)	
2	487 (11.39)	161 (8.93)	
3	231 (5.40)	58 (3.22)	
4	76 (1.78)	16 (0.89)	
Liver, *n*(%)			<.001
0	3,207 (75.04)	1,394 (77.36)	
1	324 (7.58)	195 (10.82)	
2	462 (10.81)	144 (7.99)	
3	138 (3.23)	36 (2.00)	
4	143 (3.35)	33 (1.83)	
Cardiovascular, *n*(%)			<.001
0	683 (15.98)	174 (9.66)	
1	2,431 (56.88)	1,078 (59.82)	
2	68 (1.59)	31 (1.72)	
3	314 (7.35)	174 (9.66)	
4	778 (18.20)	345 (19.15)	
Cns, *n*(%)			0.013
0	2,700 (63.17)	1,087 (60.32)	
1	730 (17.08)	344 (19.09)	
2	289 (6.76)	147 (8.16)	
3	345 (8.07)	157 (8.71)	
4	210 (4.91)	67 (3.72)	
Renal, *n*(%)			<.001
0	2,071 (48.46)	671 (37.24)	
1	956 (22.37)	495 (27.47)	
2	459 (10.74)	267 (14.82)	
3	399 (9.34)	187 (10.38)	
4	389 (9.10)	182 (10.10)	
Other			
Preiculos, M (Q_1_, Q_3_)	1.86 (1.22, 1,032.95)	1.89 (1.27, 1,817.11)	0.028
Baseexcess, M (Q_1_, Q_3_)	0.00 (−5.00, 2.00)	0.00 (−4.00, 3.00)	<.001
O2flow, M (Q_1_, Q_3_)	4.00 (2.00, 6.00)	4.00 (2.00, 9.00)	0.207
Electivesurgery, *n*(%)			0.008
0	4,137 (96.79)	1,719 (95.39)	
1	137 (3.21)	83 (4.61)	
Mechvent, *n*(%)			<.001
0	994 (23.26)	524 (29.08)	
1	3,280 (76.74)	1,278 (70.92)	

### Baseline characteristics of patients with acute respiratory failure, with or without atrial fibrillation

Some clinical and laboratory indicators of patients with atrial fibrillation are significantly higher than those of non-atrial fibrillation patients, including age, pCO₂, pH, alkali excess, TCO₂, SpO₂, Hct, Hgb, PLT, WBC, AG, HCO_3_, BUN, Cl, Cr, K, absolute basophils and absolute eosinophils. In addition, variables such as different blood cell counts, coagulation indicators, liver function tests, vital signs, GCS, OASIS, SOFA, APS III, SAPS III, Charlson comorbidity index, and Preiculos score were also recorded. As well as therapeutic factors such as oxygen flow rate, the use of non-steroidal anti-inflammatory drugs, mechanical ventilation and elective surgery.

There were statistically significant differences in ALP, total bilirubin, respiratory rate, body weight, OASIS, SOFA, APS III, SAPS III, and Preiculos scores between the two groups. The scores were higher in the atrial fibrillation group, while the other indicators were either comparable or lower.

Supplementary Figure 1 shows the correlations among the variables in the internal dataset, and Supplementary Table 2 presents the collinearity analysis. Hemoglobin and hematocrit (*r* = 0.959, *P* < 0.001), PT and INR (*r* = 0.920, *P* < 0.001), DBP and MBP (*r* = 0.909, *P* < 0.001); There was a strong correlation among 0.001), and there were significant correlations between SOFA and APSIII (*r* = 0.724), total CO₂ and alkali excess (*r* = 0.884), bicarbonate and alkali excess (*r* = 0.722), AST and ALT (*r* = 0.819), etc. (all *P* < 0.001).

The variance inflation factor (VIFs) showed significant multicollinearity in base excess, total CO₂, hematocrit, hemoglobin, SOFA, SAPSII, DBP and MBP (VIFs ranged from 11.20 to 29.73). However, the VIFs of bicarbonate, AST, ALT, total bilirubin, liver function, PT, INR and systolic blood pressure were all below 10, indicating that there was no significant multicollinearity.

Table 3 presents the selected predictor variables. Due to the differences between the MIMIC-IV and MIMIC-III databases and the lack of data on variables such as PCO₂, pH, calcium, absolute basophils, absolute neutrophils, ALT, ALP, AST, body weight, and non-steroidal anti-inflammatory drugs, despite these differences, the performance of the model was not significantly affected.

**Table 3:** Predictor variables for the internal set obtained using six selection methods.

Methods	Predictor variables
LASSO	apsiii, po2, pco2, ph, totalco2, lactate, myocardial_infarct, congestive_heart_failure, peripheral_vascular_disease, cerebrovascular_disease, dementia, chronic_pulmonary_disease, rheumatic_disease, mild_liver_disease, diabetes_without_cc, diabetes_with_cc, paraplegia, renal_disease, malignant_cancer, severe_liver_disease, metastatic_solid_tumor, aids, hematocrit, hemoglobin, platelets, wbc, aniongap, bicarbonate, bun, calcium, chloride, creatinine, glucose, sodium, potassium, abs_basophils, abs_eosinophils, abs_lymphocytes, abs_monocytes, abs_neutrophils, inr, pt, ppt, alt, alp, ast, bilirubin_total, sofa, respiration, coagulation, liver, cardiovascular, cns, renal, urineoutput, sbp, dbp, mbp, resp_rate, temperature, spo2, weight, gcs, gcs_motor, gcs_verbal, gcs_eyes, gcs_unable, nsaid, oasis, oasis_prob, age, preiculos, mechvent, electivesurgery, o2_flow, sapsii, sapsii_prob, ventilation_status
RF-MDA	age, congestive_heart_failure, pt, inr, sapsii_prob, sapsii, bun, ppt, apsiii, bilirubin_total, creatinine, sofa, oasis, nsaid, mbp, sbp, oasis_prob, ast, totalco2, dbp, renal_disease, baseexcess, severe_liver_disease, platelets, myocardial_infarct, bicarbonate, hemoglobin, lactate, liver, hematocrit
RF-MDG	age, congestive_heart_failure, pt, inr, sapsii_prob, sapsii, bun, ppt, apsiii, bilirubin_total, creatinine, sofa, oasis, nsaid, mbp, sbp, oasis_prob, ast, totalco2, dbp, renal_disease, baseexcess, severe_liver_disease, platelets, myocardial_infarct, bicarbonate, hemoglobin, lactate, liver, hematocrit
SR-FS	age, congestive_heart_failure, inr, weight, sbp, mbp, electivesurgery, ph, renal_disease, oasis, nsaid, hemoglobin, cerebrovascular_disease, glucose, preiculos, severe_liver_disease, spo2, mechvent, gcs, po2, bicarbonate, bun, calcium, aids, ast, apsiii, cns, renal, pt, diabetes_with_cc, malignant_cancer, platelets, hematocrit, sodium, abs_neutrophils, aniongap, ppt, dbp, abs_eosinophils, creatinine, dementia, oasis_prob, urineoutput, abs_lymphocytes, gcs_eyes, temperature, chloride, peripheral_vascular_disease, mild_liver_disease, bilirubin_total, lactate, paraplegia, liver, metastatic_solid_tumor, o2_flow, ventilation_status, chronic_pulmonary_disease, sapsii, potassium, sapsii_prob
SR-BS	apsiii, po2, ph, lactate, congestive_heart_failure, peripheral_vascular_disease, cerebrovascular_disease, dementia, chronic_pulmonary_disease, mild_liver_disease, diabetes_with_cc, paraplegia, renal_disease, malignant_cancer, severe_liver_disease, metastatic_solid_tumor, aids, hematocrit, hemoglobin, platelets, aniongap, bicarbonate, bun, calcium, chloride, creatinine, glucose, potassium, abs_eosinophils, abs_lymphocytes, abs_neutrophils, inr, pt, ppt, ast, bilirubin_total, liver, cns, renal, urineoutput, sbp, dbp, mbp, temperature, spo2, weight, gcs, gcs_verbal, gcs_eyes, gcs_unable, nsaid, oasis, oasis_prob, age, preiculos, mechvent, electivesurgery, o2_flow, sapsii, sapsii_prob, ventilation_status
SR-BE	abs_eosinophils, abs_lymphocytes, abs_neutrophils, age, aids, aniongap, apsiii, ast, bicarbonate, bilirubin_total, bun, calcium, cerebrovascular_disease, chloride, chronic_pulmonary_disease, cns, congestive_heart_failure, creatinine, dbp, dementia, diabetes_with_cc, electivesurgery, gcs, gcs_eyes, glucose, hematocrit, hemoglobin, inr, lactate, liver, malignant_cancer, mbp, mechvent, metastatic_solid_tumor, mild_liver_disease, nsaid, o2_flow, oasis, oasis_prob, paraplegia, peripheral_vascular_disease, ph, platelets, po2, potassium, ppt, preiculos, pt, renal, renal_disease, sapsii, sapsii_prob, sbp, severe_liver_disease, spo2, temperature, urineoutput, ventilation_status, weight

### Selection of predictor variables

The predictor variables selected by six methods are. The specific parameters of the six methods, namely LASSO, RF-MDA, RF-MDG, SR-FS, SR-BS and SR-BE, are detailed in Supplementary Tables 3–7, Supplementary Figures 3–12. Figure 2 shows the intersection of six groups of predictor variables. Select the features that appear in at least four of the six methods as the final predictor variables.

**Figure 2:**
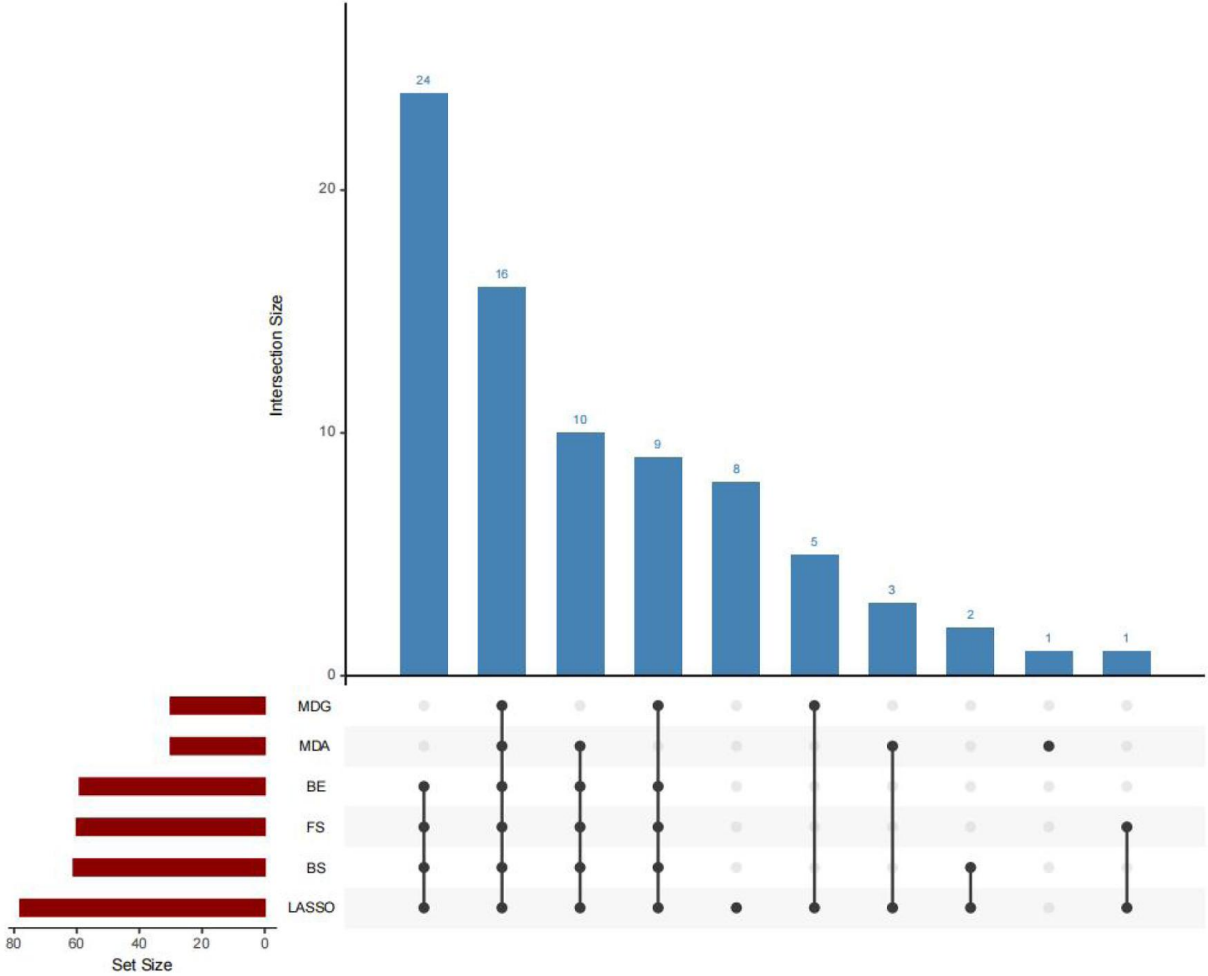
UpSet plot.

Table 3 shows various performance metrics of the six models on both the internal validation set and the external validation set.

### Model development and comparison

All internal datasets are divided into training sets and validation sets in a ratio of 4:1. Supplementary Table 6 summarizes the baseline characteristics of these two groups, showing comparable distributions. The Logistic regression (LR) results, including odds ratio and p value, are shown in Supplementary Tables 7, 8, and the corresponding forest plots are presented in Supplementary Figures 6, 7.

Table 4 presents the evaluation metrics for the internal and external validation sets along with their 95% confidence intervals. The AUC range for the internal set was 0.734–0.816, while that for the external set was 0.685–0.822. Delong tests (Supplementary Tables 9–11) identified XGBoost and Random Forest (RF) as the top performers on the internal and external datasets, respectively. Specifically, XGBoost achieved AUC values of 0.816 [0.804–0.829] and 0.771 [0.758–0.784], with SEN, SPE, PLR, NLR, PPV, NPV, and F1-score at 0.626 (0.601–0.651), 0.800 (0.785–0.815), 3.133 (2.882–3.405), 0.467 (0.436–0.501), 0.610 (0.585–0.635), 0.811 (0.797–0.825), 0.6267; 0.704 (0.684–0.725), 0.702 (0.689–0.716), 2.366 (2.247–2.501), 0.421 (0.390–0.452), 0.499 (0.478–0.520), 0.849 (0.838–0.860), 0.58. The corresponding values for the Random Forest model are 0.810[0.796–0.823], 0.822[0.811–0.834], 0.673 (0.648–0.697), 0.786 (0.771–0.810), 3.148 (3.040–3.256), 0.416 (0.378–0.450), 0.611 (0.596–0.646), 0.828 (0.813–0.842), 0.6332. 0.748 (0.728–0.766), 0.741 (0.727–0.754), 2.889 (2.718–3.057), 0.341 (0.315–0.368), 0.549 (0.529–0.569), 0.874 (0.864–0.884), 0.63. Both models performed excellently across multiple metrics, with the Random Forest model showing a slight overall advantage over XGBoost.

**Table 4:** Predictive performance metrics of different machine learning algorithms of the validation set.

Models	XGBOOST	RF	LR	DT	SVM	ANN
Internal validation set						
AUC (95%CI)	0.816 [0.804–0.829]	0.81 [0.796–0.823]	0.802 [0.789–0.816]	0.734 [ 0.719–0.75]	0.806 [ 0.793–0.819]	0.759 [ 0.744–0.774 ]0.779
Cutoff value	0.27	0.32	0.33	0.17	0.01	0.01
SEN (95%CI)	0.626 (0.601–0.651)	0.673 (0.648–0.697)	0.642 (0.618–0.667)	0.512 (0.486–0.538)	0.597 (0.572–0.622)	0.649 (95% CI: 0.624–0.673)
SPE (95%CI)	0.800 (0.785–0.815)	0.786 (0.771–0.810)	0.787 0.772–0.8)	0.840 (0.826–0.853)	0.827 (0.813–0.841)	0.666 (0.649–0.684)
PLR (95%CI)	3.133 (2.882–3.405)	3.148 (3.040–3.256)	3.009 (2.778–3.260)	3.194 (2.897–3.521)	3.447 (3.149–3.773)	1.944 (1.824–2.073)
NLR (95%CI)	0.467 (0.436–0.501)	0.416 (0.378–0.450)	0.455 (0.423–0.489)	0.581 (0.550–0.614)	0.487 (0.457–0.520)	0.527 (0.489–0.568)
PPV (95%CI)	0.610 (0.585–0.635)	0.611 (0.596–0.646)	0.600 (0.587–0.635)	0.614 (0.587–0.642)	0.632 (0.607–0.658)	0.492 (0.470–0.515)
NPV (95%CI)	0.811 (0.797–0.825)	0.828 (0.813-0.842)	0.815 (0.813–0.842)	0.775 (0.761–0.790)	0.804 (0.790–0.819)	0.792 (0.776–0.808)
F1 score	0.6267	0.6332	0.627	0.4685	0.5571	0.6811
External validation set						
AUC (95%CI)	0.771:0.758–0.784)	0.822 (0.811–0.834)	0.742 (0.729–0.755)	0.685 (0.671–0.697)	0.750 (0.737–0.764)	0.739 (0.725-0.752)
Cutoff value	0.17	0.36	0.26	0.28	0.66	0.229
SEN (95%CI)	0.704 (0.684–0.725)	0.748 (0.728–0.766)	0.752 (0.734–0.771)	0.644 (0.621–0.667)	0.691 (0.651–0.729)	0.658 (0.636–0.677)
SPE (95%CI)	0.702 (0.689–0.716)	0.741 (0.727–0.754)	0.620 (0.606–0.634)	0.677 (0.663–0.690)	0.744 (0.732–0.755)	0.700 (0.687–0.713)
PLR (95%CI)	2.366 (2.247–2.501)	2.889 (2.718–3.057)	1.981 (1.900–2.074)	1.993 (1.889–2.110)	2.696 (2.657–2.718)	2.191 (2.071–2.313)
NLR (95%CI)	0.421 (0.390–0.452)	0.341 (0.315–0.368)	0.400 (0.367–0.431)	0.526 (0.491–0.561)	0.416 (0.359–0.477)	0.489 (0.458–0.521)
PPV (95%CI)	0.499 (0.478–0.520)	0.549 (0.529–0.569)	0.455 (0.439–0.472)	0.457 (0.438–0.475)	0.216 (0.197–0.236)	0.480 (0.460–0.500)
NPV (95%CI)	0.849 (0.838–0.860)	0.874 (0.864–0.884)	0.856 (0.844–0.867)	0.818 (0.806–0.831)	0.959 (0.953–0.965)	0.829 (0.817–0.840)
F1 score	0.58	0.63	0.57	0.53	0.22	0.56

**Figure 3:**
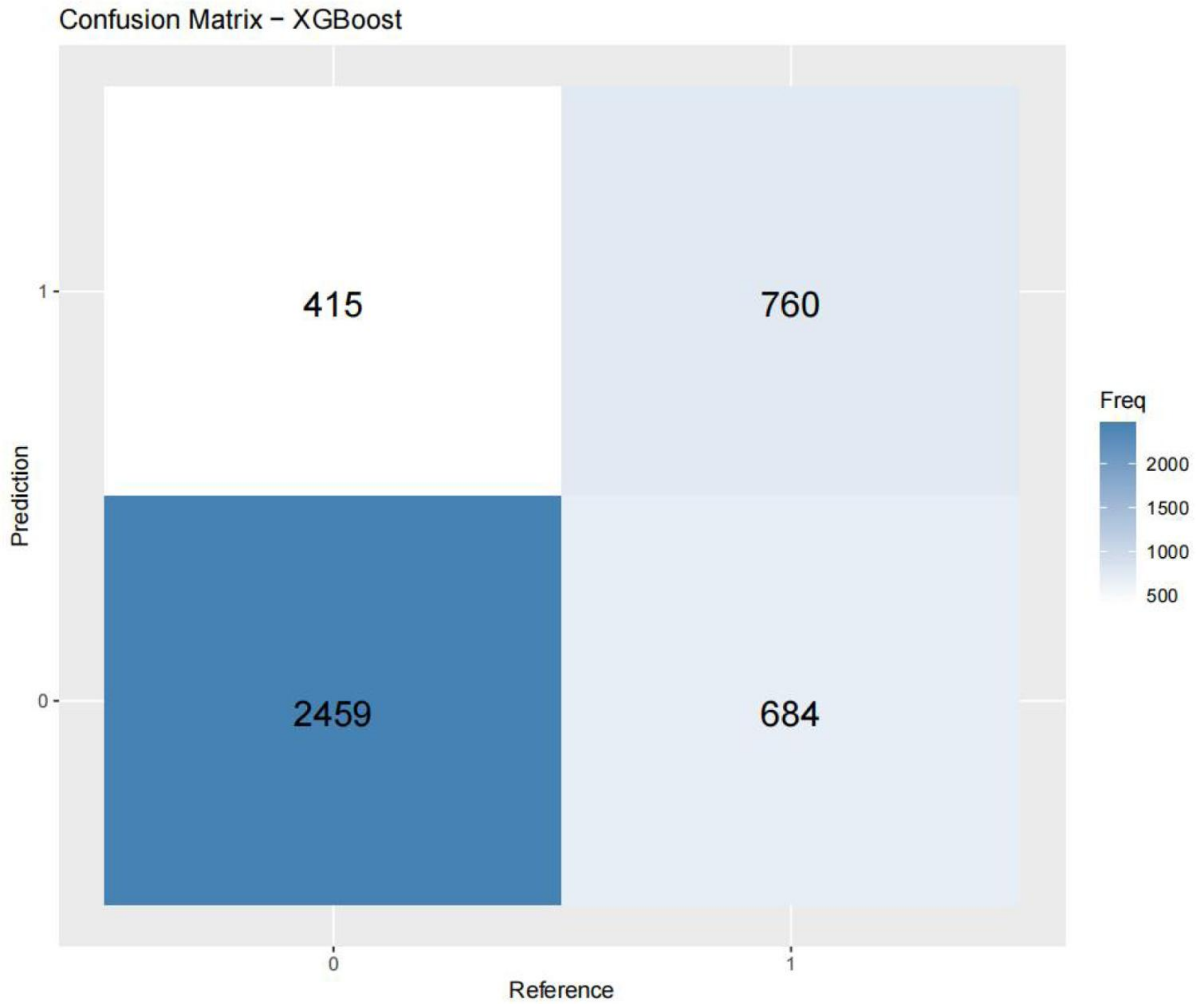
Confusion matrix- XGBoost.

**Figure 4:**
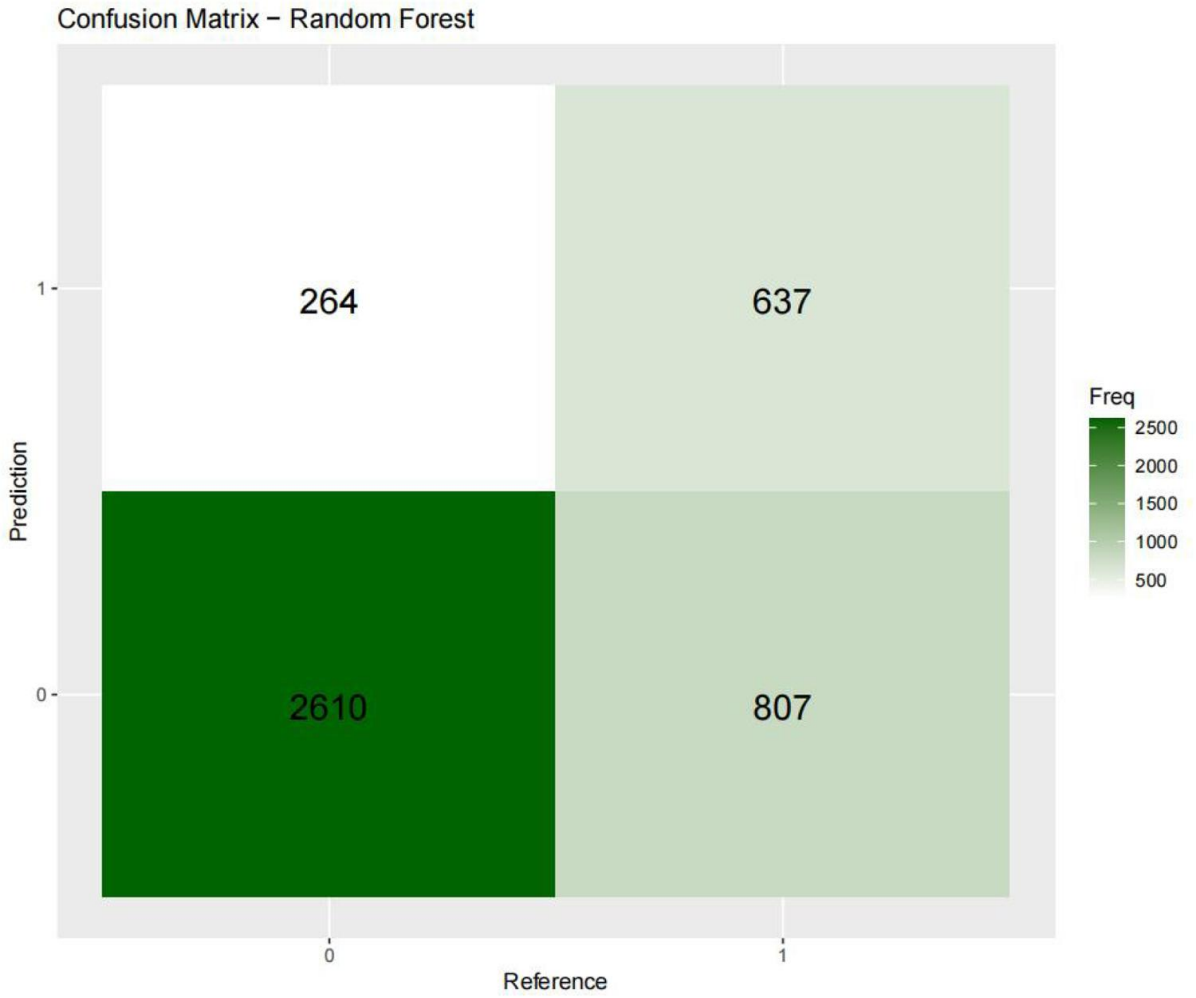
Confusion matrix-random forest.

**Figure 5:**
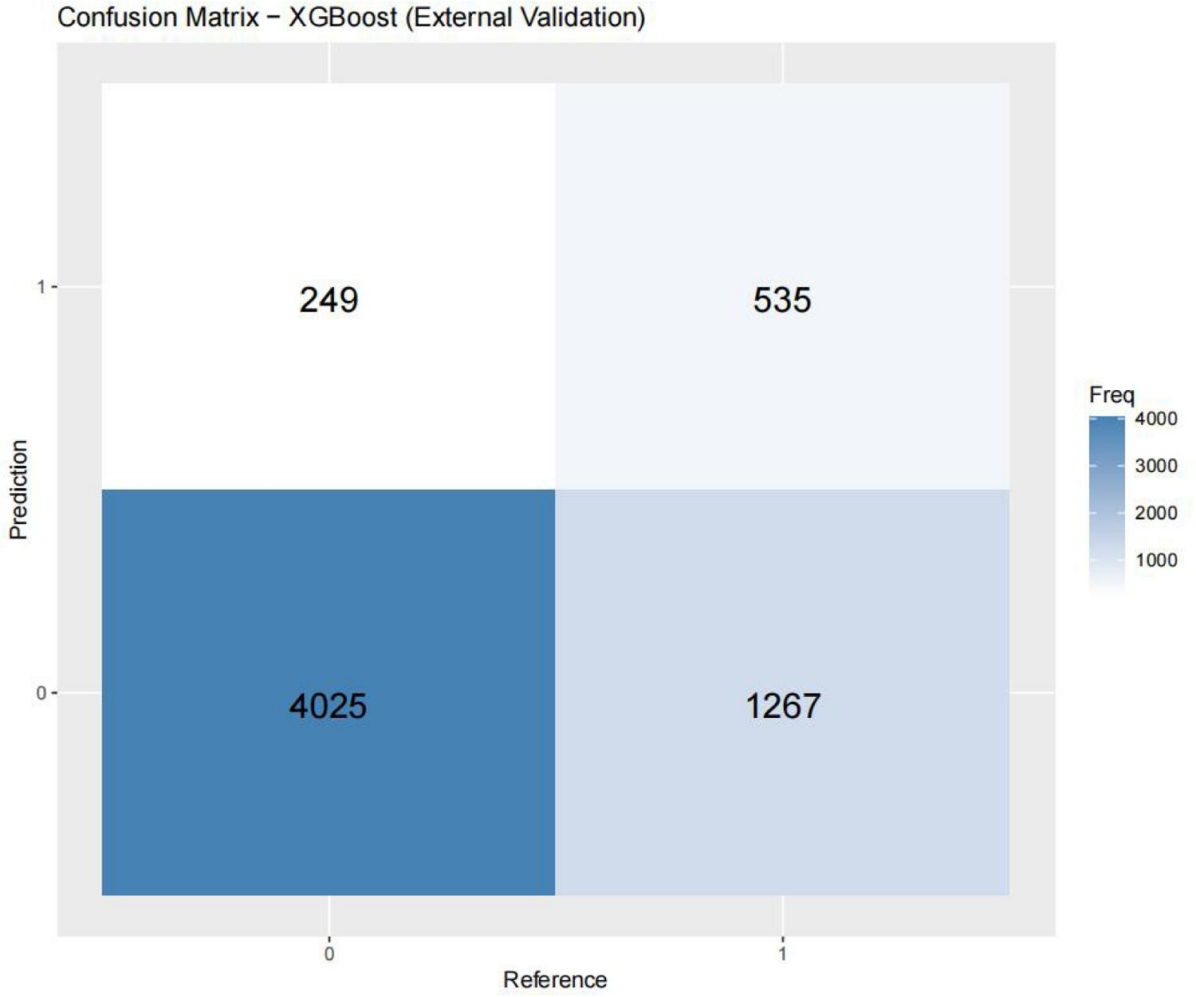
Confusion matrix-XGBoost (external validation).

**Figure 6:**
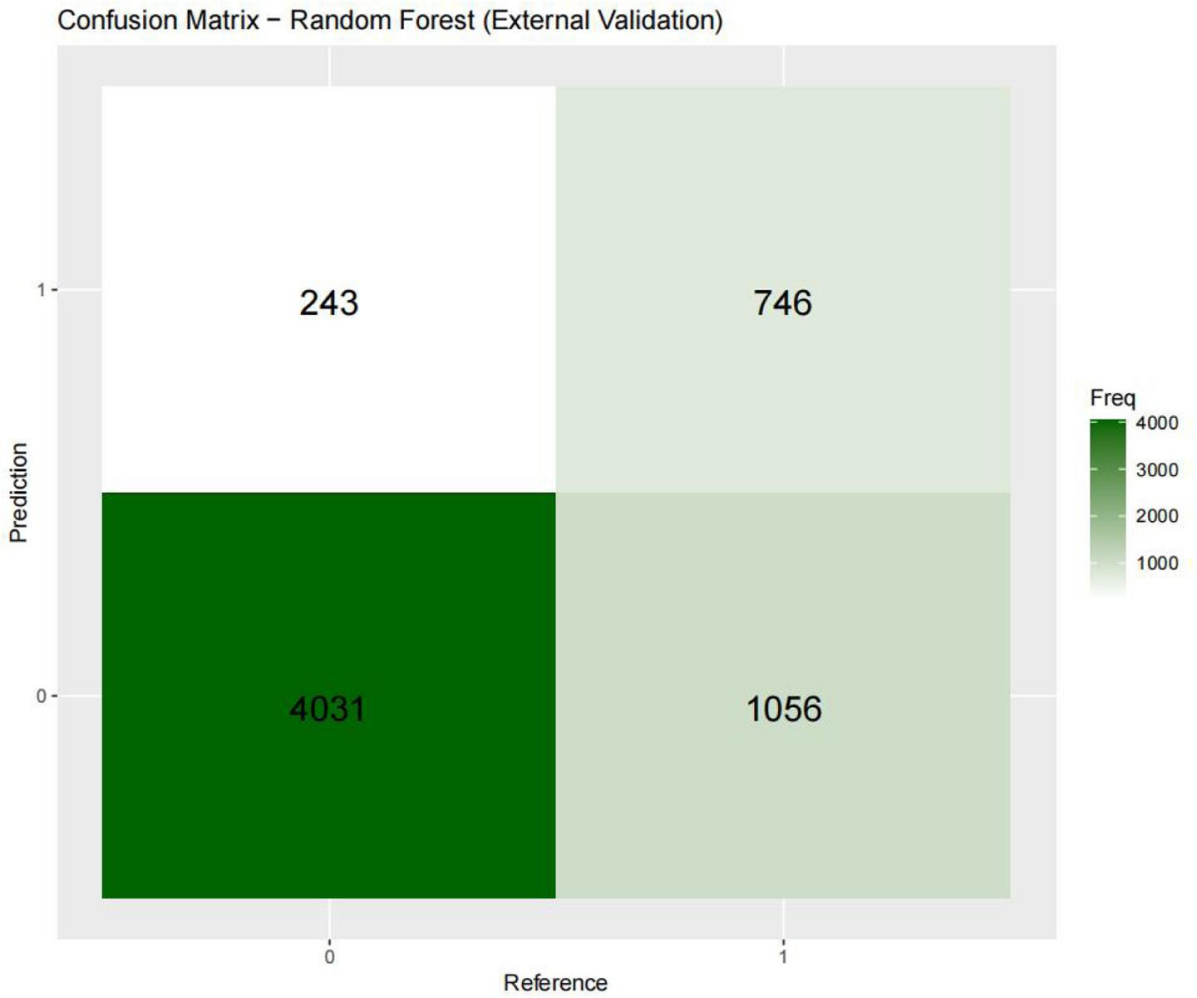
Confusion matrix-random forest (external validation).

Figures 3–6 displays the confusion matrices for the internal validation set and external validation set of the two optimal models (XGBOOST and RF).

For the internal validation set, the RF metric is: Sensitivity (SEN) 0.673[0.648–0.697], specificity (SPE) 0.786[0.771–0.810], positive likelihood ratio (PLR) 3.148[3.040–3.256], negative likelihood ratio (NLR) 0.416[0.378–0.450] The positive predictive value (PPV) was 0.611[0.596–0.646], the negative predictive value (NPV) was 0.828 [0.813–0.842], and the f1 score was 0.633. For the external validation set, the metrics of the RF model are: SEN = 0.748 [0.728–0.766], SPE = 0.741 [0.727–0.754], PLR = 2.889 [2.718–3.057], NLR = 0.341 [0.315–0.368] PPV = 0.549 [0.529–0.569], NPV = 0.874 [0.864–0.884], F1-Score = 0.63.

**Figure 7:**
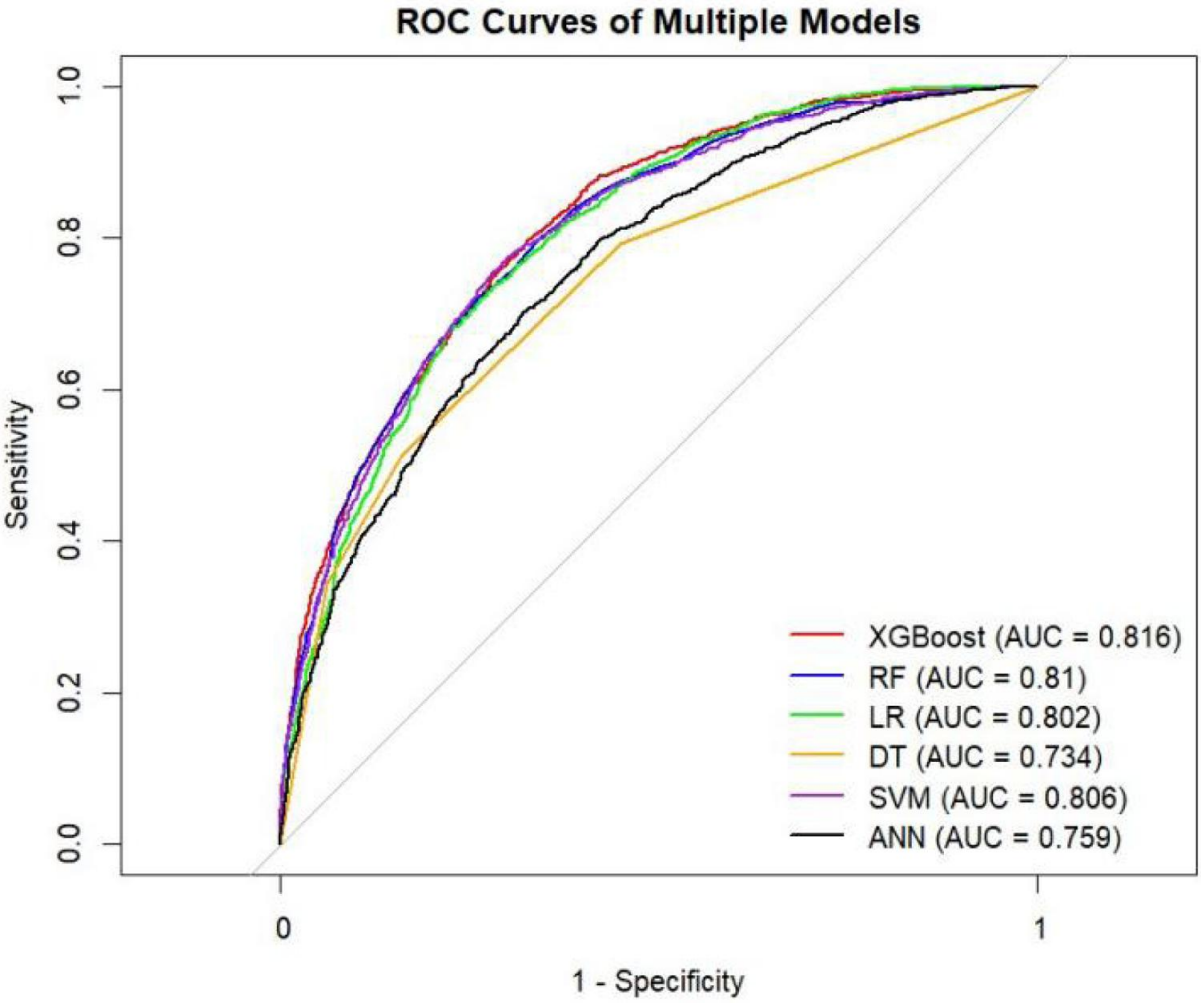
ROC plot.

**Figure 8:**
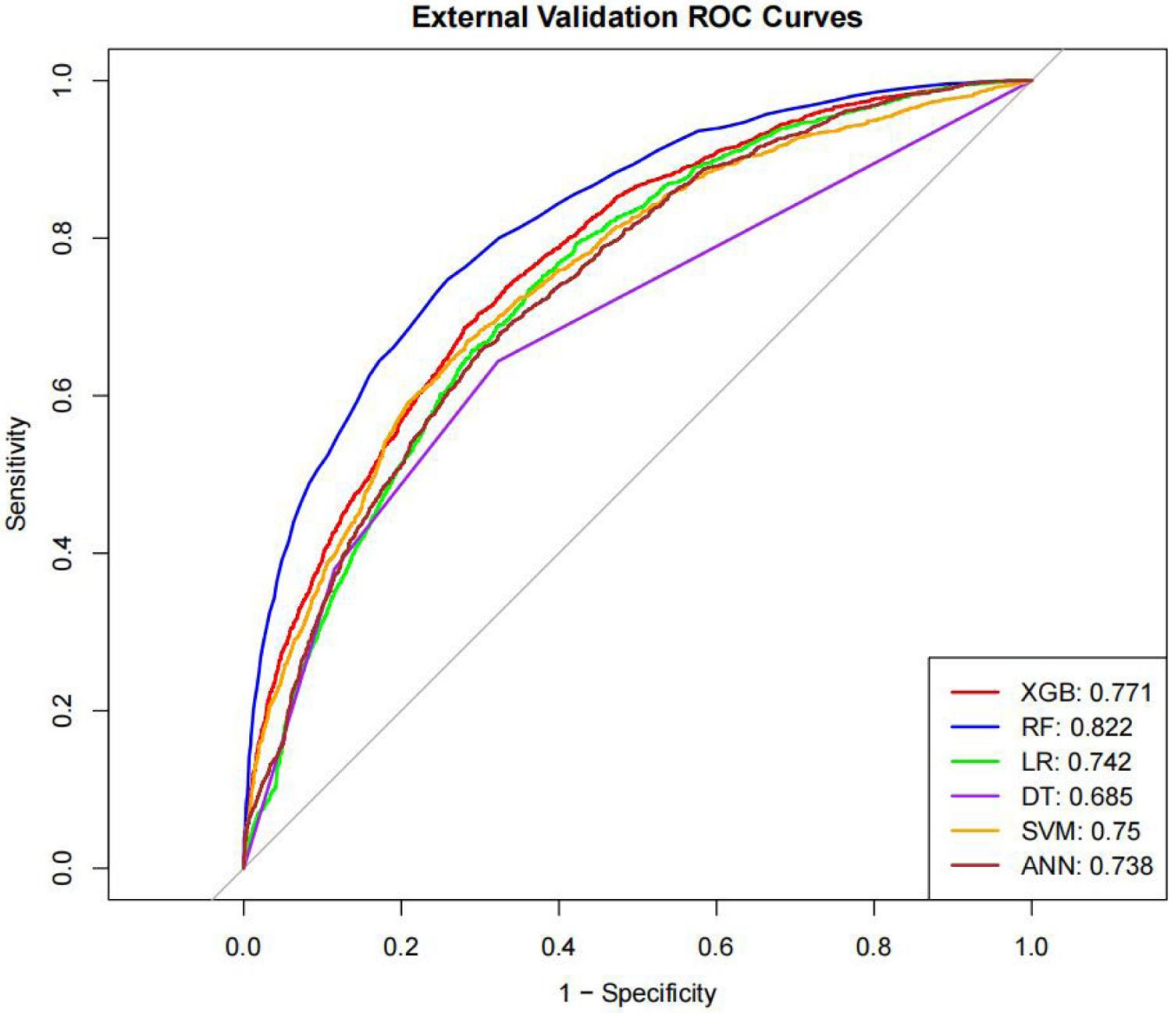
ROC plot.

**Figure 9:**
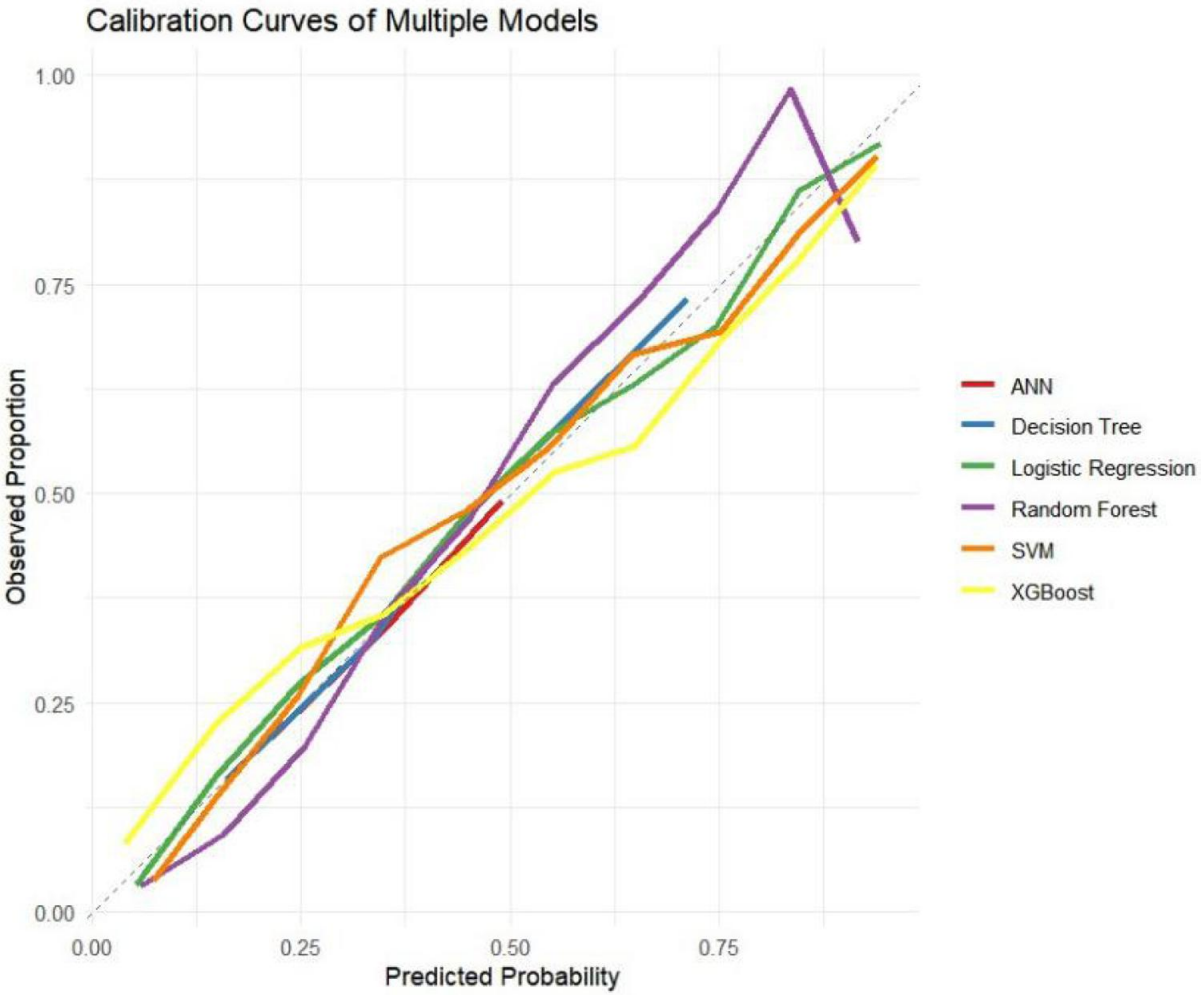
Calibration curves.

**Figure 10:**
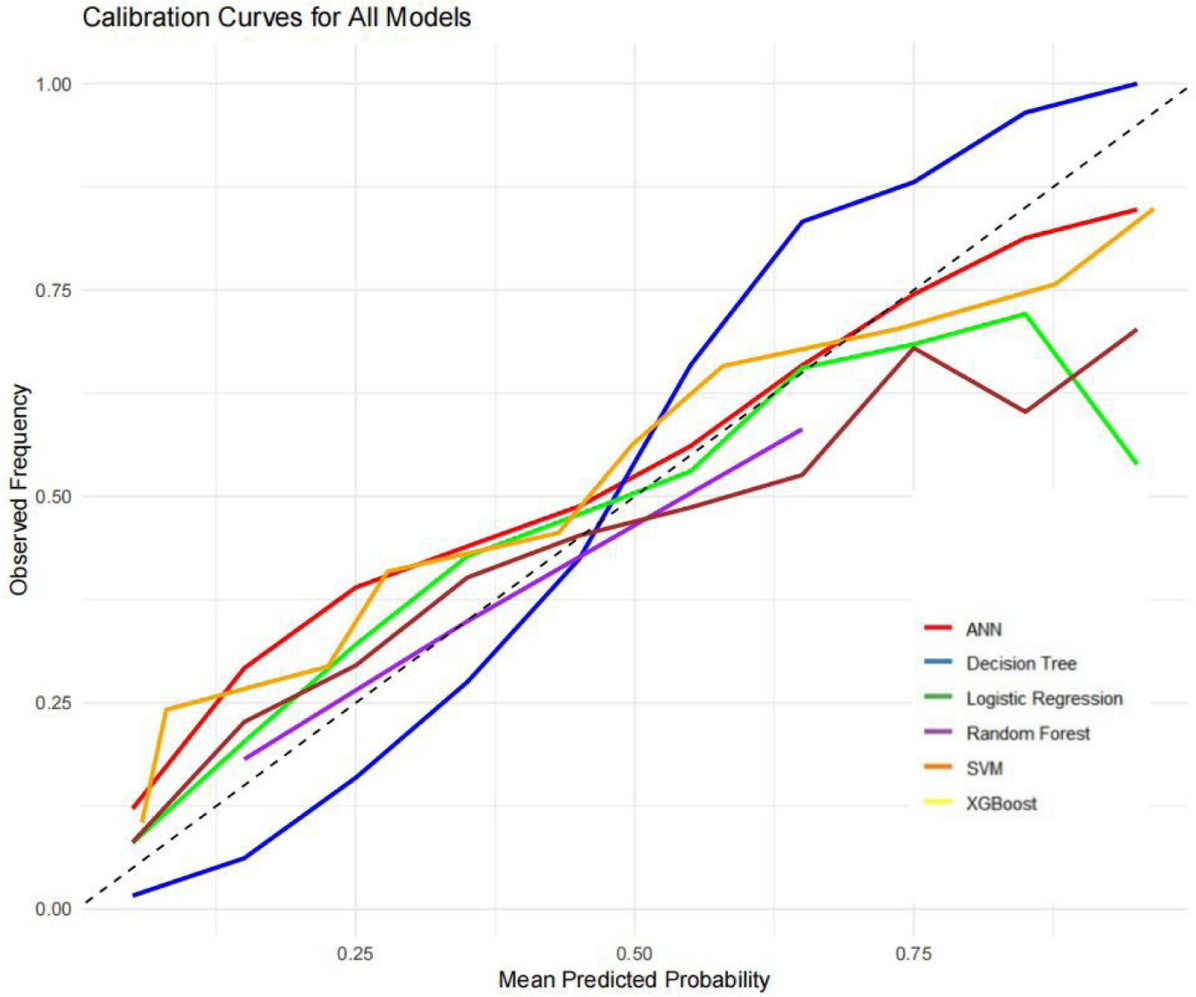
Calibration curves.

**Figure 11:**
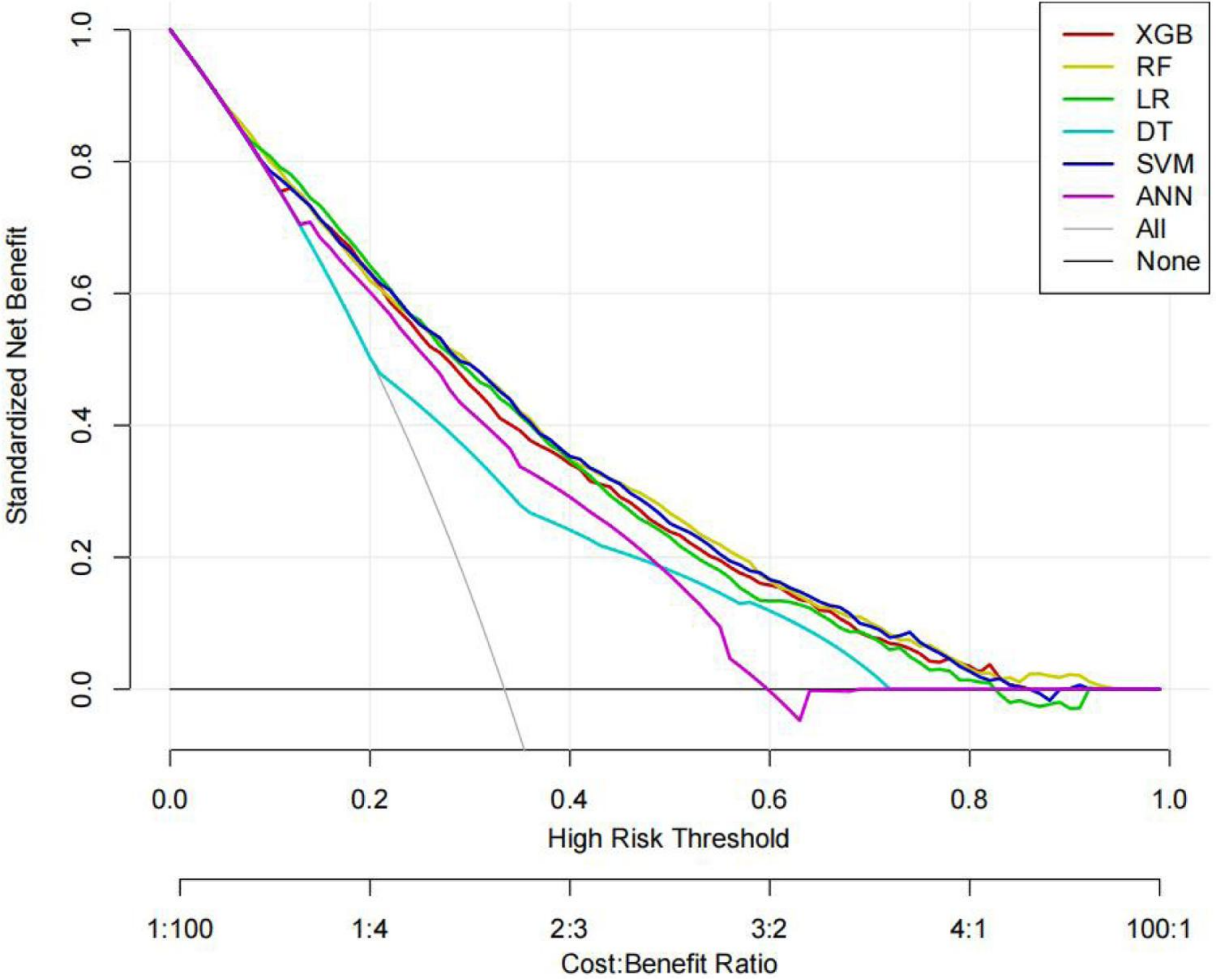
DCA plot.

**Figure 12:**
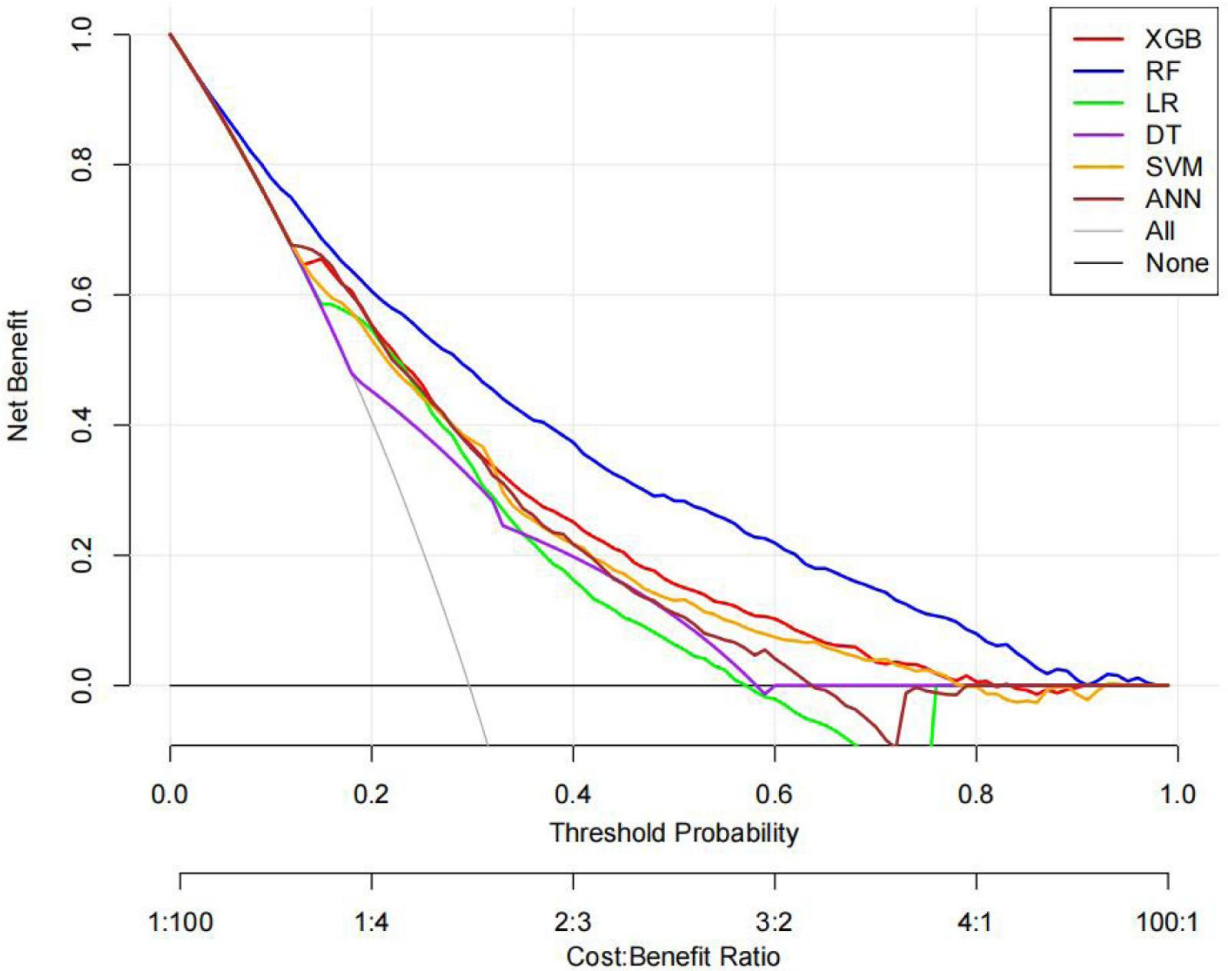
DCA plot.

The ROC curves, correction curves and DCA curves of the six models in the internal validation set and the external validation set are shown in Figures 7–12.

The superior performance of XGBoost and Random Forest (RF) models can be attributed to several factors. Firstly, the AUC values of both models are relatively high (XGBoost: 0.816, RF: 0.831), indicating that the classification capabilities of the two models are strong and they have a good ability to distinguish between atrial fibrillation and non-atrial fibrillation patients. Secondly, their calibration curves are closely aligned with the ideal diagonal, indicating a more accurate probability estimation compared to other models. Finally, the two models demonstrated higher net benefits in the decision curve analysis (DCA) within the threshold probability range, indicating that they have greater clinical utility in guiding decision-making. These advantages highlight the accuracy, reliability and practical value of XGBoost and RF in predicting atrial fibrillation in patients with acute respiratory failure.

### Optimal model interpretability

This study developed two dynamic graphs (available at http://127.0.0.1:7312) to visualize the impact of various variables on disease outcomes and to assist clinicians in quickly determining the likelihood of atrial fibrillation (Supplementary Figures 13–16). Variables with extreme values (representing a small portion of the data) were not excluded because determining the upper limit of clinical trial indicators is challenging.

**Figure 13:**
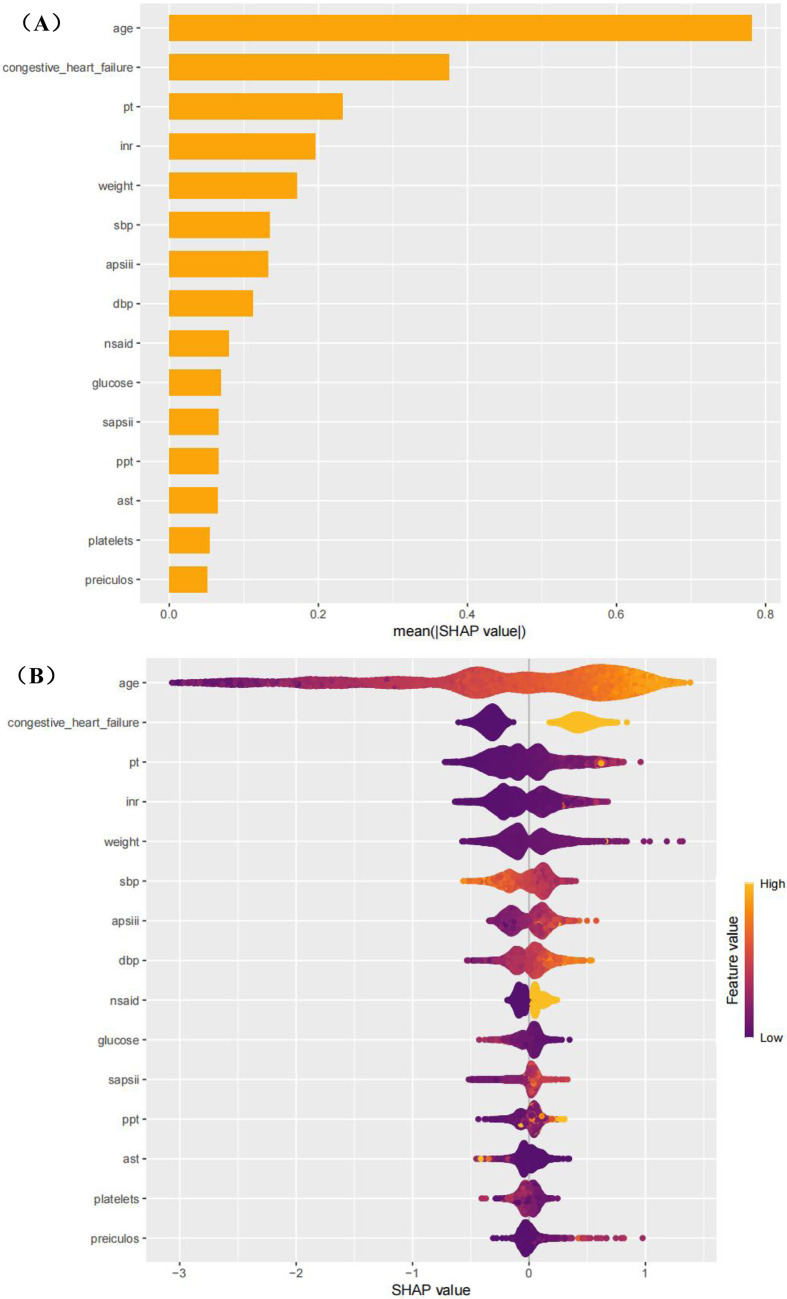
SHAP plot.

To better understand the influence of different variables on atrial fibrillation, we used the SHapley Additive explanation diagram (Figure 13). As shown in Figures 8A,B, age is the most important factor influencing the development of atrial fibrillation. Figure 13B also highlights the intensity and direction of the influence of each variable. For the convenience of local interpretation, Figures 8C,D show how specific variables increase or decrease the risk of atrial fibrillation.

The clinical application of this model (highlighted by the SHAP summary graph) provides valuable insights into how individual characteristics can contribute to prediction and thereby assist in clinical decision-making. Key characteristics, such as age, blood glucose level and platelet count, play a crucial role in the prognosis of patients. The high interpretability of this model enables clinicians to understand the impact of each feature on risk prediction, thereby supporting informed real-time decision-making during patient management.

For instance, clinicians can guide the treatment adjustments of diabetic or metabolic patients based on the blood sugar levels predicted by the model to prevent complications. Systolic blood pressure (SBP) and body weight can be monitored to enable targeted intervention for patients with cardiovascular risk. Age is regarded as a key predictive feature that can assist clinicians in assessing high-risk patients, as it is closely related to chronic diseases such as hypertension, diabetes and cardiovascular diseases. This allows for more personalized care and effective treatment strategies.

For elderly patients, especially those with a history of heart failure, this model provides insights that help clinicians assess the risks and benefits of specific intervention measures. By combining age-related risks with other clinical data such as AST levels and systolic blood pressure, clinicians can make timely and accurate treatment decisions, prevent over-prescripting, and ensure that high-risk situations are addressed.

In addition, the model also provides predictive insights into biomarkers such as AST levels, which are potential signals of liver dysfunction. This enables clinicians to proactively adjust treatment methods and prevent health problems from escalating. Integrating these insights into clinical practice can enhance the accuracy of diagnosis and improve the ability of clinicians to intervene effectively at an early stage.

In conclusion, this model enhances clinical decision-making by providing real-time, data-driven insights. It enhances the accuracy of intervention, helps identify high-risk patients at an early stage, and optimizes treatment plans, all of which contribute to improving the prognosis of patients. Its ability to offer tailor-made proactive care makes it a valuable tool for patient management

## Discussion

This study is the first to propose a predictive model for acute respiratory failure combined with atrial fibrillation, integrating six feature selection methods with real-world vital signs to identify key risk factors. Unlike previous research, no comparison model was included, as no predictive models for this condition currently exist. Instead, the study systematically identifies the most relevant risk factors using multiple methods, ensuring model robustness and reliability.

Schüttler et al. simulated human atrial fibrillation using modeling techniques (9). Roussos et al. highlighted that patients with respiratory failure often experience neural suppression and neuromuscular conduction disorders, which may be indicative of neurotransmitter disturbances associated with AF and myocardial dysfunction (10). However, the mechanisms by which respiratory failure induces AF remain poorly understood.

Interestingly, the predictive performance of our machine learning models was slightly higher in the external validation cohort compared with the internal validation cohort. This finding may reflect differences in the distribution of patient characteristics between datasets, or suggest that the models are robust and generalizable across independent patient populations. Nevertheless, careful interpretation is warranted, and further prospective validation is needed to confirm model stability and clinical applicability. Given the large sample size of this study, the likelihood of model overfitting is relatively low. Large-scale datasets can adequately reflect the variability and representativeness of the underlying population, enabling machine learning algorithms to capture true patterns rather than noise. Nevertheless, external validation remains crucial for confirming the model's robustness and generalization capabilities.

Six machine learning algorithms were applied, with performance evaluated using various metrics. XGBoost and Random Forest (RF) emerged as the top performers. A key innovation of this study is its novel approach to addressing a gap in existing research. The model's interpretability was enhanced through SHAP values and visualizations, making it a valuable tool for healthcare professionals. The study found that age is the primary risk factor for atrial fibrillation, consistent with previous research (21). Age plays a crucial role in the development of atrial fibrillation (AF) in patients with acute respiratory failure (ARF). As age increases, the heart's atrial structure undergoes dilation and fibrosis, leading to instability in atrial electrophysiological properties and an increased risk of AF. Elderly patients often have comorbid chronic conditions such as hypertension, diabetes, and coronary heart disease, with hypertension, in particular, causing left atrial enlargement and electrical remodeling, further promoting the occurrence of AF. Additionally, the aging process is accompanied by a decline in the heart's self-regulation ability, making elderly patients more susceptible to electrophysiological abnormalities during acute pathological states such as hypoxia and acid-base imbalance. The mechanisms through which ARF leads to AF primarily include hypoxemia, acid-base imbalance, and hypercapnia. Hypoxia triggers instability in cardiac electrical activity, acid-base imbalance (e.g., metabolic acidosis) alters myocardial electrical activity, and hypercapnia causes atrial dilation and increased load, all of which contribute to the onset of AF. Furthermore, ARF-induced hemodynamic changes, particularly in patients with concomitant heart failure, increase atrial pressure and stretch the atrial walls, further exacerbating the risk of AF. Pulmonary hypertension and elevated left atrial pressure also play key roles. The interaction of these pathological mechanisms makes ARF a significant trigger for AF, and understanding these mechanisms better helps in the prevention and management of these patients. Additionally, factors such as prothrombin time (PT), international normalized ratio (INR), blood pressure (DBP and SBP), body weight, glucose, hemoglobin, partial thromboplastin time (PPT), aspartate aminotransferase (AST), and bilirubin were identified as reversible risk factors for atrial fibrillation. For patients without other systemic issues, these values should be carefully managed within an appropriate range.

Other studies have shown that when patients develop atrial fibrillation, certain indicators can be used to predict the likelihood of their atrial fibrillation resolving (19). Age, gender, duration of AF, and other factors are important predictive indicators that can be used to assess a patient's recovery from AF.

## Limitations

This study has several limitations. Firstly, the external validation set lacks some predictors, which may affect the performance of the model. Future research should utilize more comprehensive external databases and improve data collection to reduce missing values, possibly through advanced imputation or multi-center data.

Thirdly, The respiratory and circulatory system diseases discussed in this study are both strongly associated with smoking (20). However, due to certain limitations in the MIMIC database, the inability to obtain this important characteristic represents a significant shortcoming of this research.

Secondly, factors not related to acute respiratory failure, such as previous cardiovascular diseases, medications or genetics, may affect the occurrence of atrial fibrillation. Future research should incorporate more covariates and consider multi-factor models or propensity scoring methods to address confusion.

Finally, the samples are from the MIMIC database and may not represent the global population, which limits their universality. More diverse populations need to be studied to validate the model in different regions.

## Conclusion

Six feature selection methods and six machine learning algorithms are adopted to establish the model. XGBoost and Random Forest demonstrated the best performance in external validation. Vital signs and laboratory data can assist in timely clinical decision-making, reduce complications and improve outcomes.

## Data Availability

The original contributions presented in the study are included in the article/Supplementary Material, further inquiries can be directed to the corresponding author.

## References

[1] Fujishima S. Guideline-based management of acute respiratory failure and acute respiratory distress syndrome. J Intensive Care. (2023) 11(1):10. 10.1186/s40560-023-00658-3 36895001 PMC9998250

[2] Bordignon S Chiara Corti M Bilato C. Atrial fibrillation associated with heart failure, stroke and mortality. J Atr Fibrillation. (2012) 5(1):467. 10.4022/jafib.467 28496747 PMC5153082

[3] Mentes O Celik D Yıldız M Özdemir T Ari M Aksoy Güney EN Atrial fibrillation among ICU patients with type 2 respiratory failure: who is at risk and what are the outcomes? Diagnostics (Basel). (2025) 15(13):1612. 10.3390/diagnostics15131612 40647611 PMC12248686

[4] Sagris M Vardas EP Theofilis P Antonopoulos AS Oikonomou E Tousoulis D. Atrial fibrillation: pathogenesis, predisposing factors, and genetics. Int J Mol Sci. (2021) 23(1):6. 10.3390/ijms23010006 35008432 PMC8744894

[5] Bemis CE Serur JR Borkenhagen D Sonnenblick EH Urschel CW. Influence of right ventricular filling pressure on left ventricular pressure and dimension. Circ Res. (1974) 34(4):498–504. 10.1161/01.res.34.4.498 4826926

[6] Choi SE Sagris D Hill A Lip GYH Abdul-Rahim AH. Atrial fibrillation and stroke. Expert Rev Cardiovasc Ther. (2023) 21(1):35–56. 10.1080/14779072.2023.2160319 36537565

[7] Liang F Wang Y. Coronary heart disease and atrial fibrillation: a vicious cycle. Am J Physiol Heart Circ Physiol. (2021) 320(1):H1–12. 10.1152/ajpheart.00702.2020 33185113

[8] Aldrugh S Sardana M Henninger N Saczynski JS McManus DD. Atrial fibrillation, cognition and dementia: a review. J Cardiovasc Electrophysiol. (2017) 28(8):958–65. 10.1111/jce.13261 28569383 PMC5783292

[9] Schüttler D Bapat A Kääb S Lee K Tomsits P Clauss S Animal models of atrial fibrillation. Circ Res. (2020) 127(1):91–110. 10.1161/CIRCRESAHA.120.316366 32716814

[10] Roussos C Koutsoukou A. Respiratory failure. Eur Respir J Suppl. (2003) 47:3s–14. 10.1183/09031936.03.00038503 14621112

[11] Van Gelder IC Rienstra M Bunting KV Casado-Arroyo R Caso V Crijns HJGM 2024 ESC guidelines for the management of atrial fibrillation developed in collaboration with the European Association for Cardio-Thoracic Surgery (EACTS). Eur Heart J. (2024) 45(36):3314–414. 10.1093/eurheartj/ehae176 39210723

[12] Roca O Hernández G Díaz-Lobato S Acute respiratory failure: clinical diagnosis and management recommendations. Intensive Care Med. (2022) 48(6):677–94. 10.1007/s00134-022-06723-x

[13] Ferrer M Antonelli M Roca O Chest imaging in acute respiratory failure: European respiratory society statement. Eur Respir J. (2019) 54(3):1900205. 10.1183/13993003.00205-2019 31248958

[14] van der Velden RMJ Hermans ANL Pluymaekers NAHA Gawalko M Elliott A Hendriks JM Dyspnea in patients with atrial fibrillation: mechanisms, assessment and an interdisciplinary and integrated care approach. Int J Cardiol Heart Vasc. (2022) 42:101086. 10.1016/j.ijcha.2022.101086 35873859 PMC9304702

[15] Swanson K Wu E Zhang A Alizadeh AA Zou J. From patterns to patients: advances in clinical machine learning for cancer diagnosis, prognosis, and treatment. Cell. (2023) 186(8):1772–91. 10.1016/j.cell.2023.01.035 36905928

[16] Guan C Gong A Zhao Y Yin C Geng L Liu L Interpretable machine learning model for new-onset atrial fibrillation prediction in critically ill patients: a multi-center study. Crit Care. (2024) 28(1):349. 10.1186/s13054-024-05138-0 39473013 PMC11523862

[17] Johnson AEW Bulgarelli L Shen L Gayles A Shammout A Horng S MIMIC-IV, a freely accessible electronic health record dataset. Sci Data. (2023) 10(1):1. 10.1038/s41597-022-01899-x 36596836 PMC9810617

[18] Johnson AE Pollard TJ Shen L Lehman LW Feng M Ghassemi M MIMIC-III, a freely accessible critical care database. Sci Data. (2016) 3:160035. 10.1038/sdata.2016.35 27219127 PMC4878278

[19] Mariani MV Pierucci N Trivigno S Cipollone P Piro A Chimenti C Probability score to predict spontaneous conversion to sinus rhythm in patients with symptomatic atrial fibrillation when less could be more? J Clin Med. (2024) 13(5):1470. 10.3390/jcm13051470 38592704 PMC10934271

[20] Wu Z Tang C Wang D. Bidirectional two-sample Mendelian randomization study of association between smoking initiation and atrial fibrillation. Tob Induc Dis. (2024) 22. 10.18332/tid/189380 PMC11186308 38899119

[21] Wang N Yu Y Sun Y Zhang H Wang Y Chen C Acquired risk factors and incident atrial fibrillation according to age and genetic predisposition. Eur Heart J. (2023) 44(47):4982–93. 10.1093/eurheartj/ehad615 37723974 PMC10719493

