# Supplementary material for: Predicting atrial fibrillation in patients with acute respiratory failure using machine learning: application of the MIMIC-III and MIMIC-IV datasets
Source: Front Cardiovasc Med. 2025 Oct 9;12:1696609. doi: 10.3389/fcvm.2025.1696609 (PMC12546187; doi:10.3389/fcvm.2025.1696609)
Supplement: Supplementary file 3 [file Datasheet3.docx]

## Supplementary Figures

### Figure. S1. The correlation heatmap of continuous variables.

Abbreviations:Po2,Partial pressure of oxygen;Pco2,Partial pressure of carbon dioxide;PH.Acidity;BE,Base Excess;Total_co2,Total Carbon Dioxide;SPo2,Peripheral Capillary Oxygen Saturation;Platelets,Platelet count;Wbc,White blood cell count;Bun,Blood Urea Nitrogen;Inr.International Normalized Ratio;Pt,Prothrombin Time;Ppt,Partial Thromboplastin Time;Alt,Alanine Aminotransferase;Alp,Alkaline Phosphatase;Ast,Aspartate Aminotransferase;Sbp,Systolic Blood Pressure;Dbp,Diastolic Blood Pressure;Mbp,Mean Blood Pressure;Resp Rate,Respiratory Rate;Gcs,Glasgow Coma Scale;Gcs Motor,GCS Motor Response;Gcs Verbal,GCS Verbal Response;Gcs Eyes,GCS Eye Opening;Gcs Unable,GCS Unable to Score;Sofa,Sequential Organ Failure Assessment


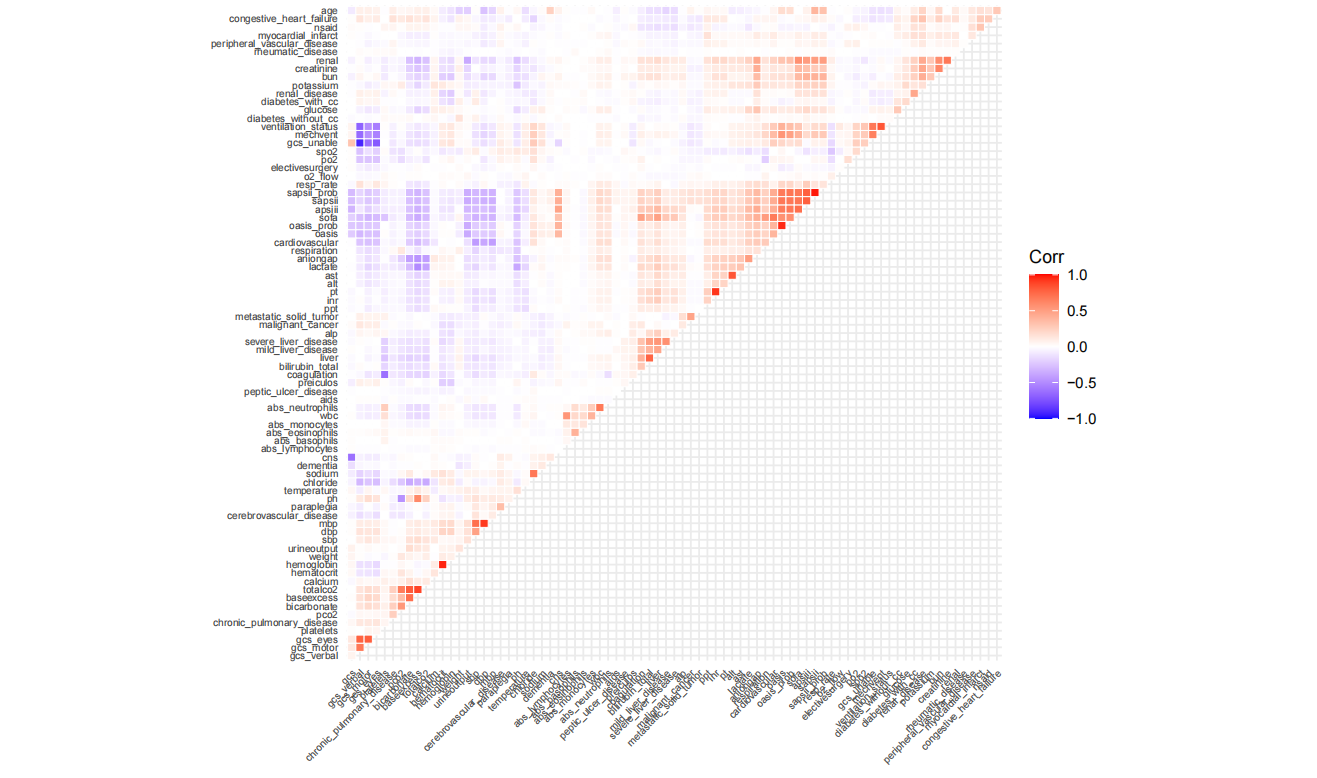


### Figure. S2. LASSO regression analysis plot. (A) Cross-validation plot for penalty parameters (λ). (B) Regression coefficient plot for predictor variables with different penalty parameters (λ).


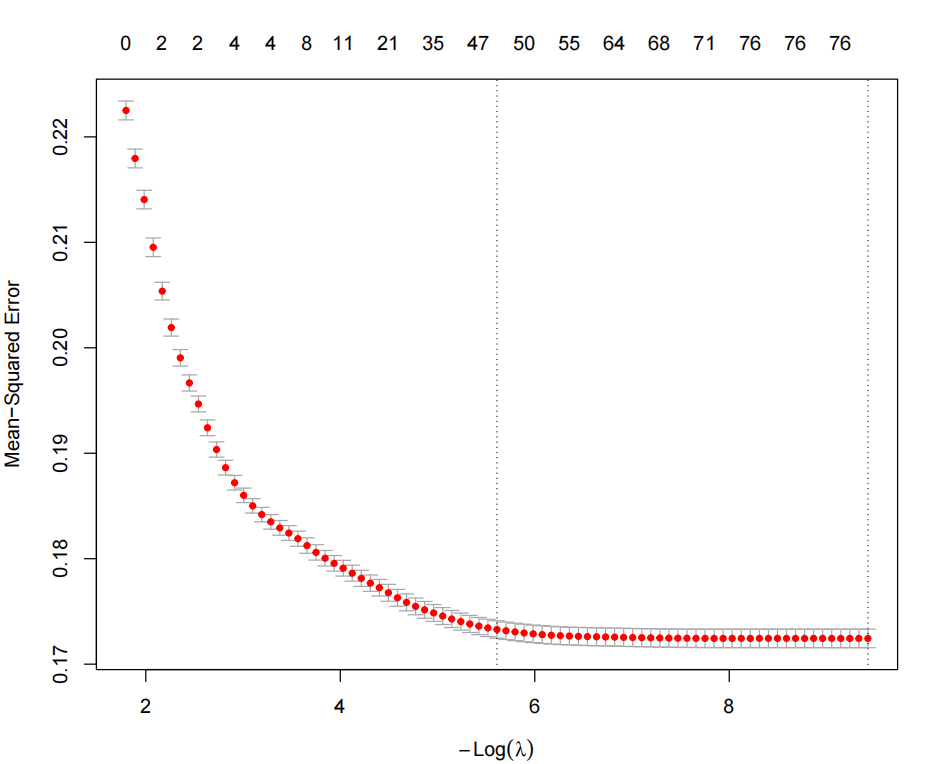


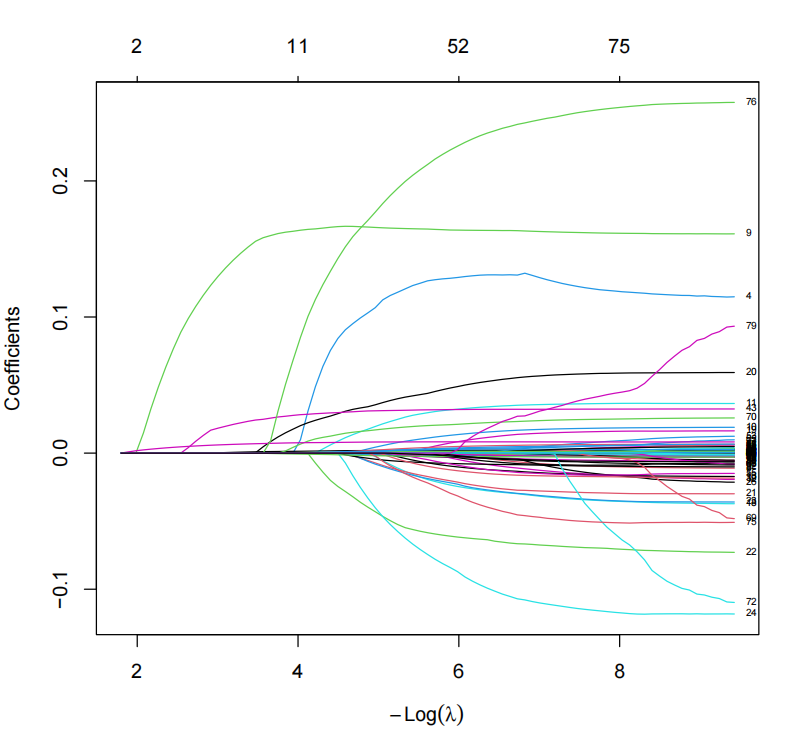


### Figure. S3. XGBoost variable importance plot ( Internal validation model and External validation model ).

Abbreviations:Inr.International Normalized Ratio;Pt,Prothrombin Time;Dbp,Diastolic Blood Pressure;Sbp,Systolic Blood Pressure;Ppt,Partial Thromboplastin Time;SPo2,Peripheral Capillary Oxygen Saturation;Ast,Aspartate Aminotransferase;Apsiii, Acute Physiology and Chronic Health Evaluation;Mbp,Mean Blood Pressure;Po2,Partial pressure of oxygen;Bun,Blood Urea Nitrogen;oasis,Open Source Anonymized Simulator;Gcs,Glasgow Coma Scale;Gcs Eyes,GCS Eye Opening.


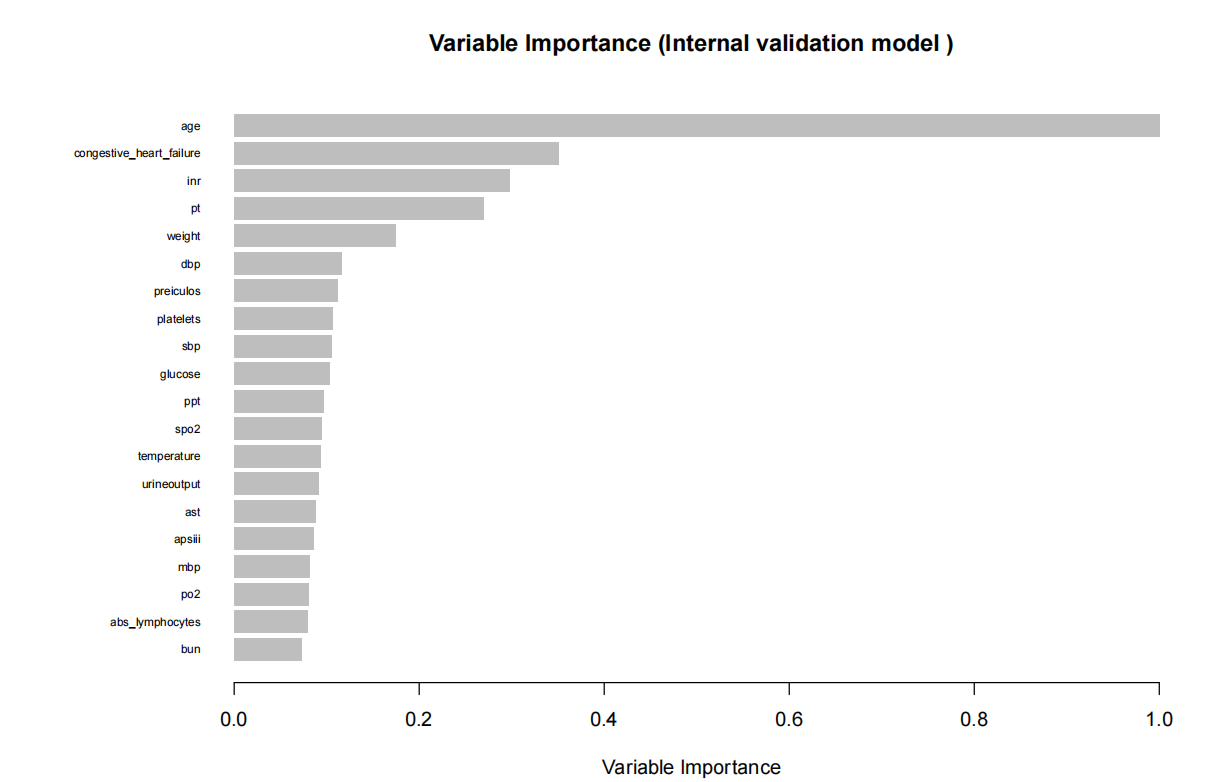


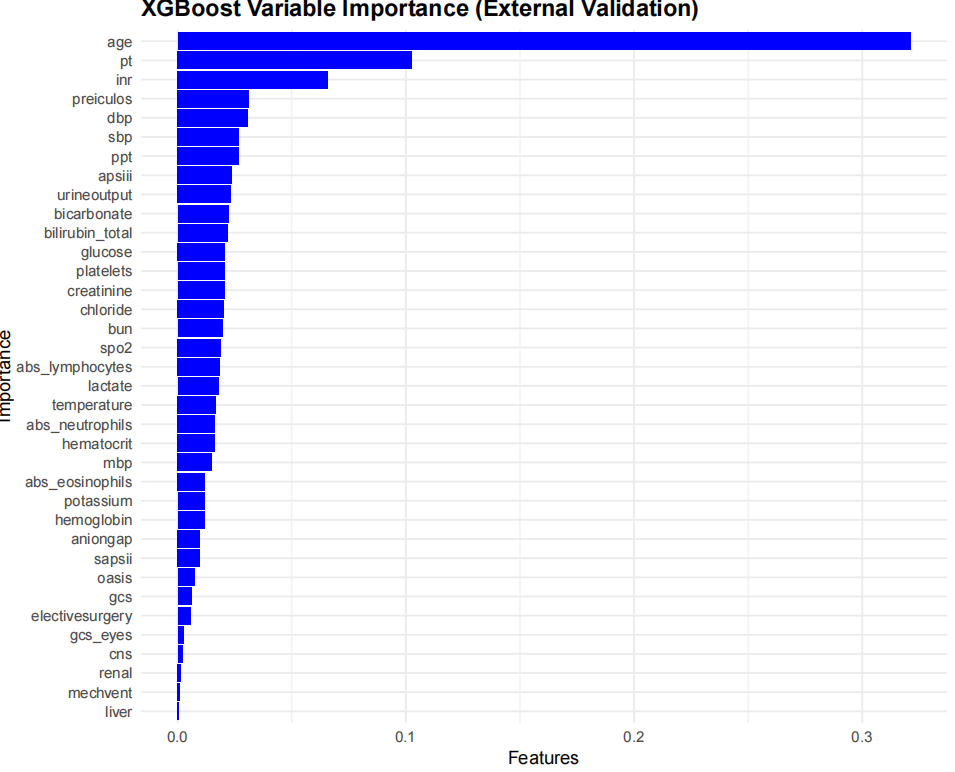


### Figure. S4. Random forest variable importance plot（Internal validation model）

Abbreviations:Pt,Prothrombin Time;Inr.International Normalized Ratio;Sapsii,Simplified Acute Physiology Score II；Apsiii, Acute Physiology and Chronic Health Evaluation;Bun,Blood Urea Nitrogen;Ppt,Partial Thromboplastin Time;Sbp,Systolic Blood Pressure;Dbp,Diastolic Blood Pressure;Mbp,Mean Blood Pressure;PH.Acidity;SPo2,Peripheral Capillary Oxygen Saturation;Gcs,Glasgow Coma Scale;Po2,Partial pressure of oxygen;Gcs Eyes,GCS Eye Opening.


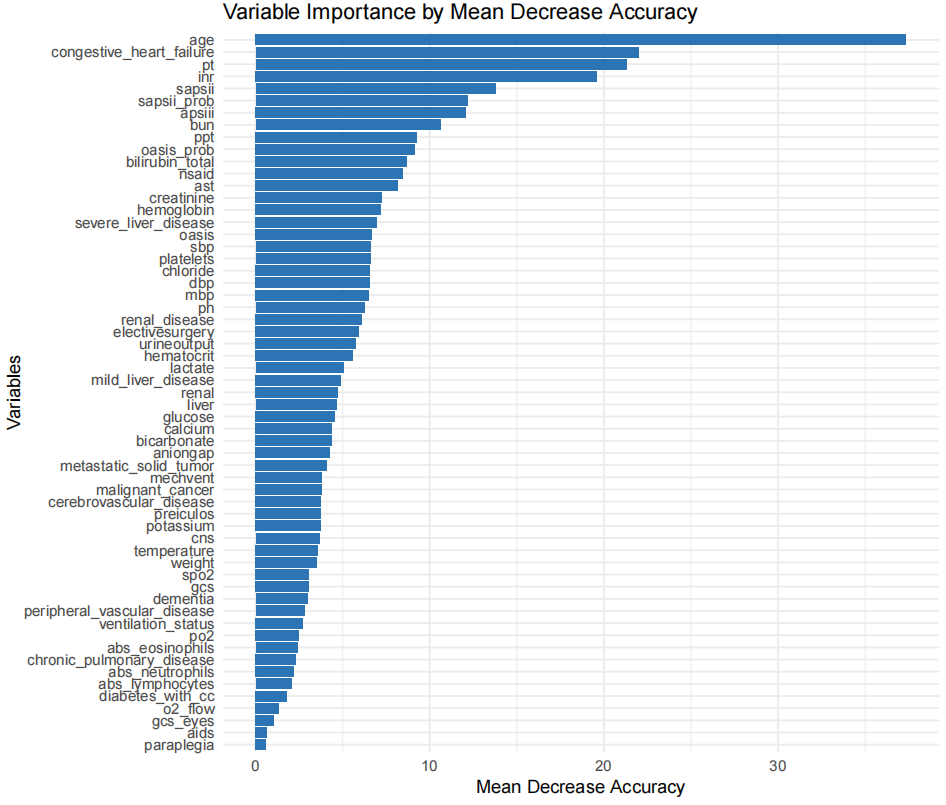


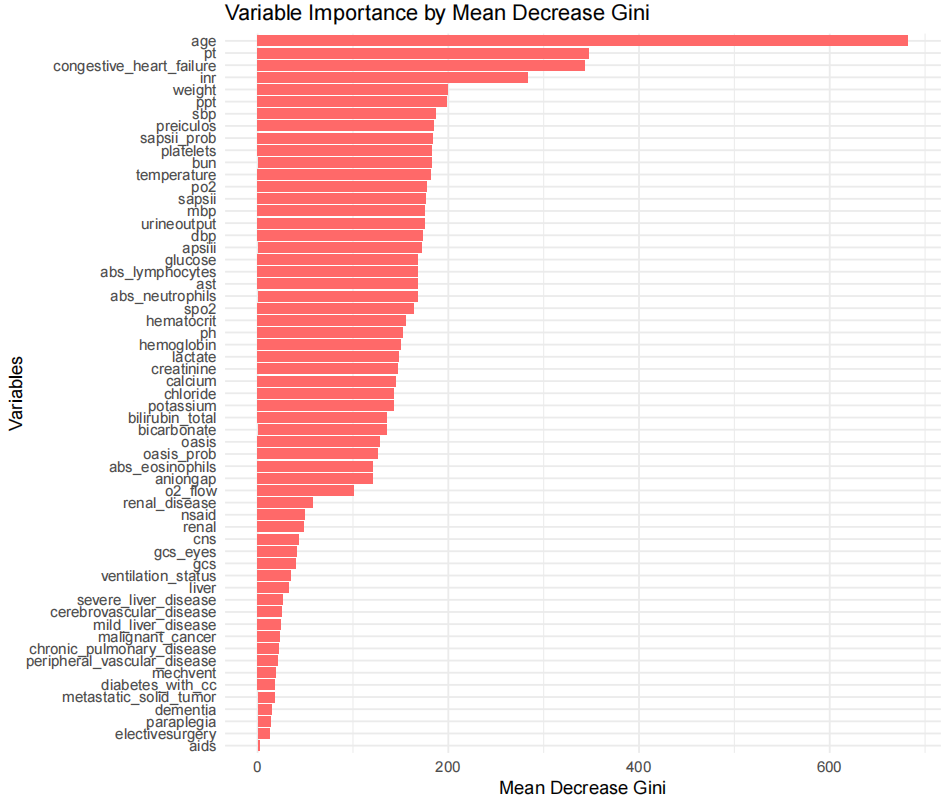


### Figure. S5.Random forest variable importance plot（External validation model）

Abbreviations:Pt,Prothrombin Time;Inr.International Normalized Ratio;Apsiii, Acute Physiology and Chronic Health Evaluation;Sapsii,Simplified Acute Physiology Score II；Dbp,Diastolic Blood Pressure;Bun,Blood Urea Nitrogen;Ppt,Partial Thromboplastin Time;;Mbp,Mean Blood Pressure;oasis,Open Source Anonymized Simulator;Sbp,Systolic Blood Pressure;SPo2,Peripheral Capillary Oxygen Saturation;Gcs,Glasgow Coma Scale;

;Gcs Eyes,GCS Eye Opening.


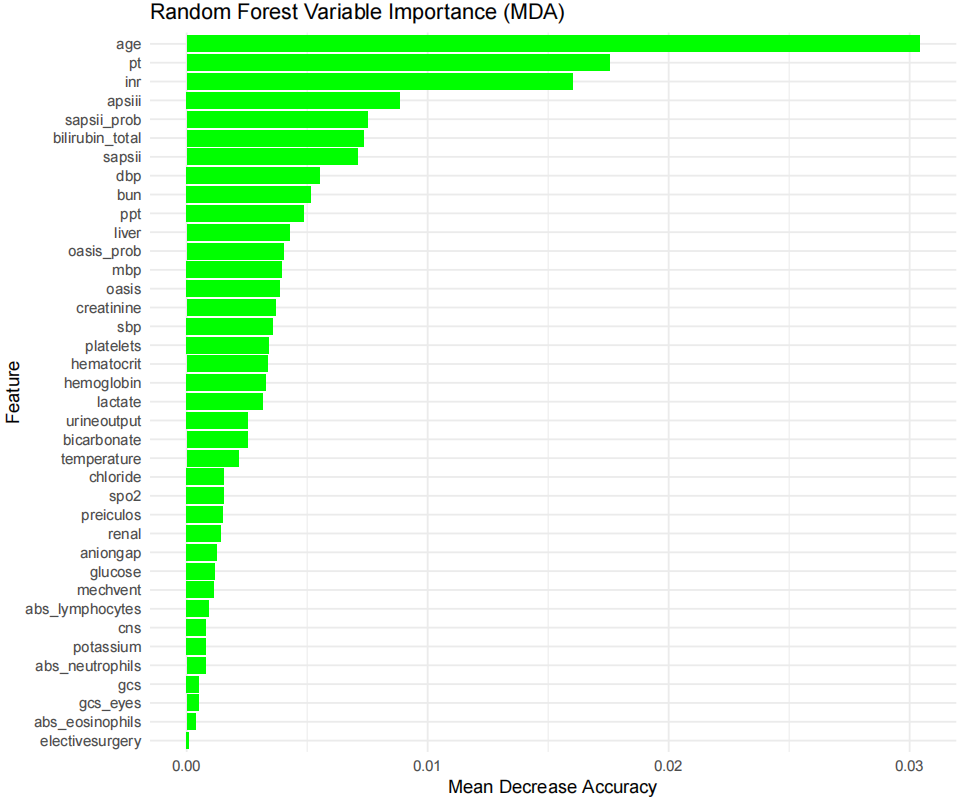


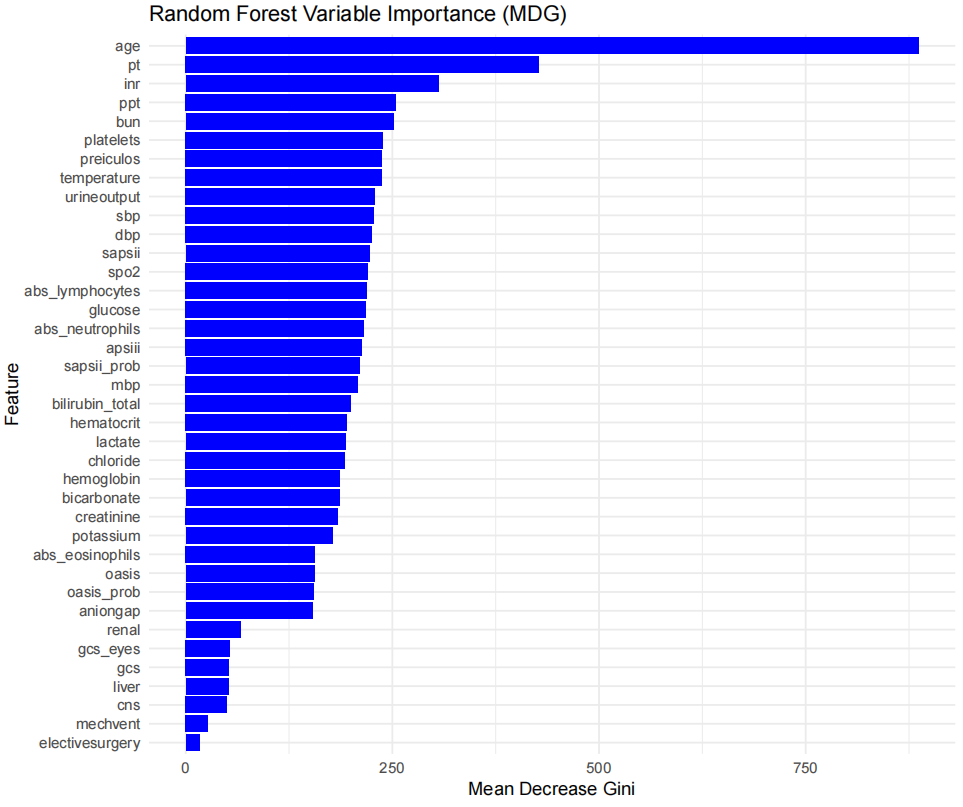


### Figure. S6. Forest plot of the logistic regression model (Internal validation model).

Abbreviations:Apsiii, Acute Physiology and Chronic Health Evaluation;Bun,Blood Urea Nitrogen;Dbp,Diastolic Blood Pressure;Gcs,Glasgow Coma Scale;Gcs Eyes,GCS Eye Opening;Inr.International Normalized Ratio;Mbp,Mean Blood Pressure;oasis,Open Source Anonymized Simulator;Ppt,Partial Thromboplastin Time;Pt,Prothrombin Time;Sapsii,Simplified Acute Physiology Score II；Sbp,Systolic Blood Pressure;SPo2,Peripheral Capillary Oxygen Saturation.


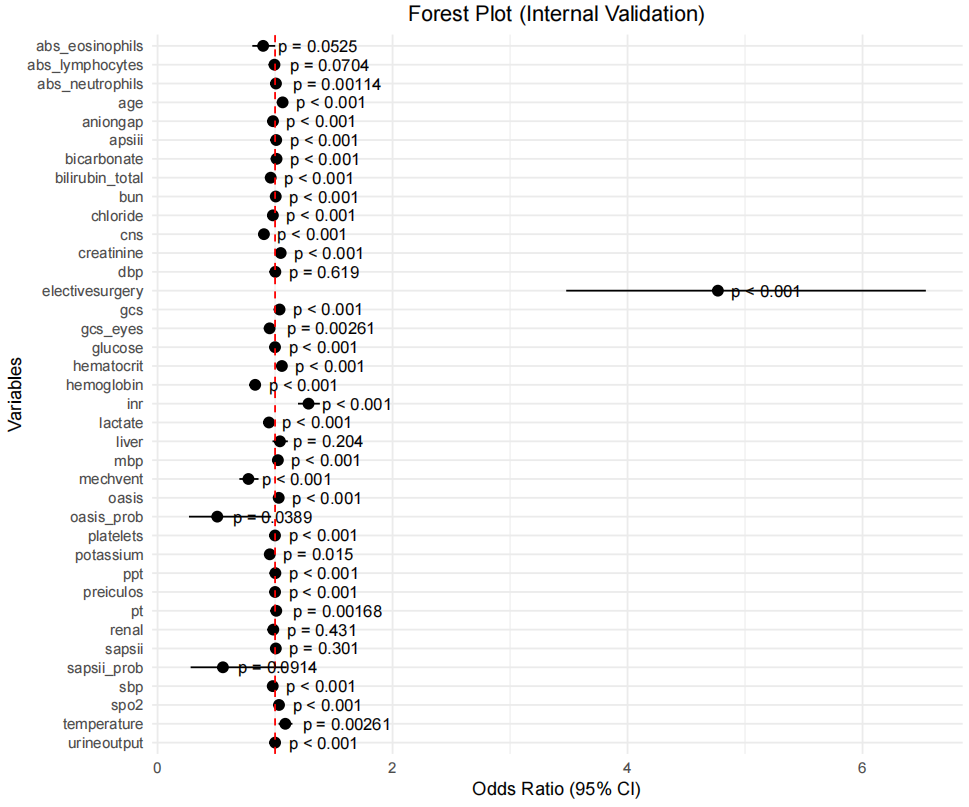


### Figure. S7. Forest plot of the logistic regression model (External validation model).

Abbreviations:Apsiii, Acute Physiology and Chronic Health Evaluation;Bun,Blood Urea Nitrogen;Dbp,Diastolic Blood Pressure;Gcs,Glasgow Coma Scale;Gcs Eyes,GCS Eye Opening;Inr.International Normalized Ratio;Mbp,Mean Blood Pressure;oasis,Open Source Anonymized Simulator;Ppt,Partial Thromboplastin Time;Pt,Prothrombin Time;Sapsii,Simplified Acute Physiology Score II；Sbp,Systolic Blood Pressure;SPo2,Peripheral Capillary Oxygen Saturation.


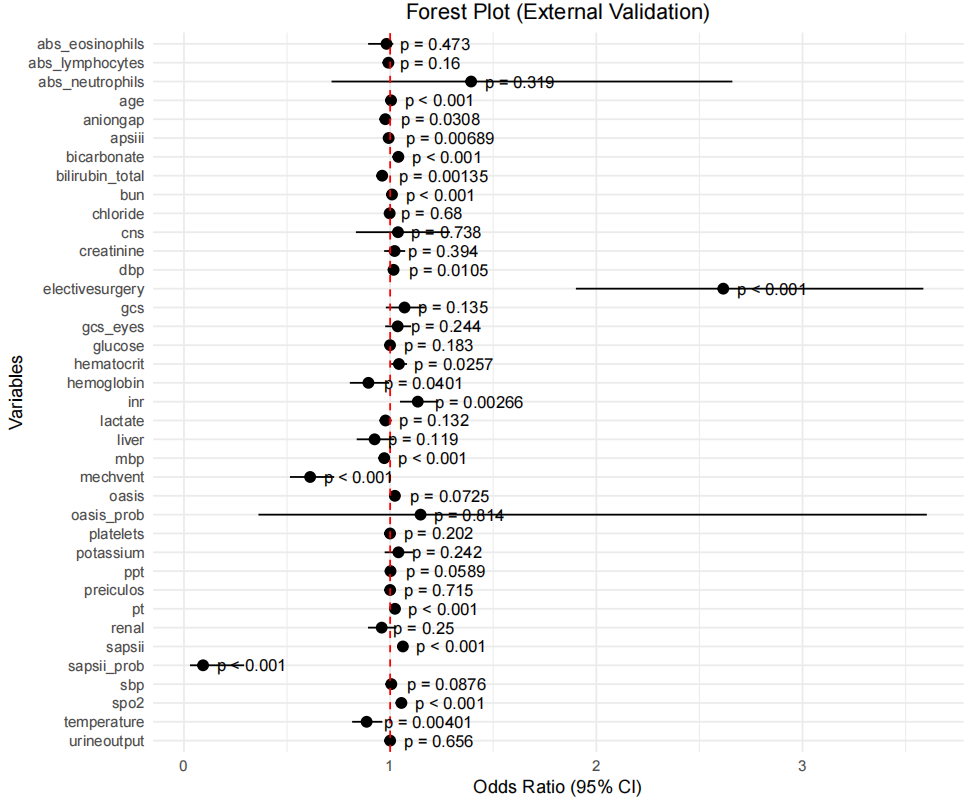


### Figure. S8. Decision tree visualization and variable importance plots (Internal validation model).

Abbreviations:Inr.International Normalized Ratio;Pt,Prothrombin Time;Sapsii,Simplified Acute Physiology Score II；Bun,Blood Urea Nitrogen;Ppt,Partial Thromboplastin Time;Sbp,Systolic Blood Pressure;Dbp,Diastolic Blood Pressure;oasis,Open Source Anonymized Simulator.


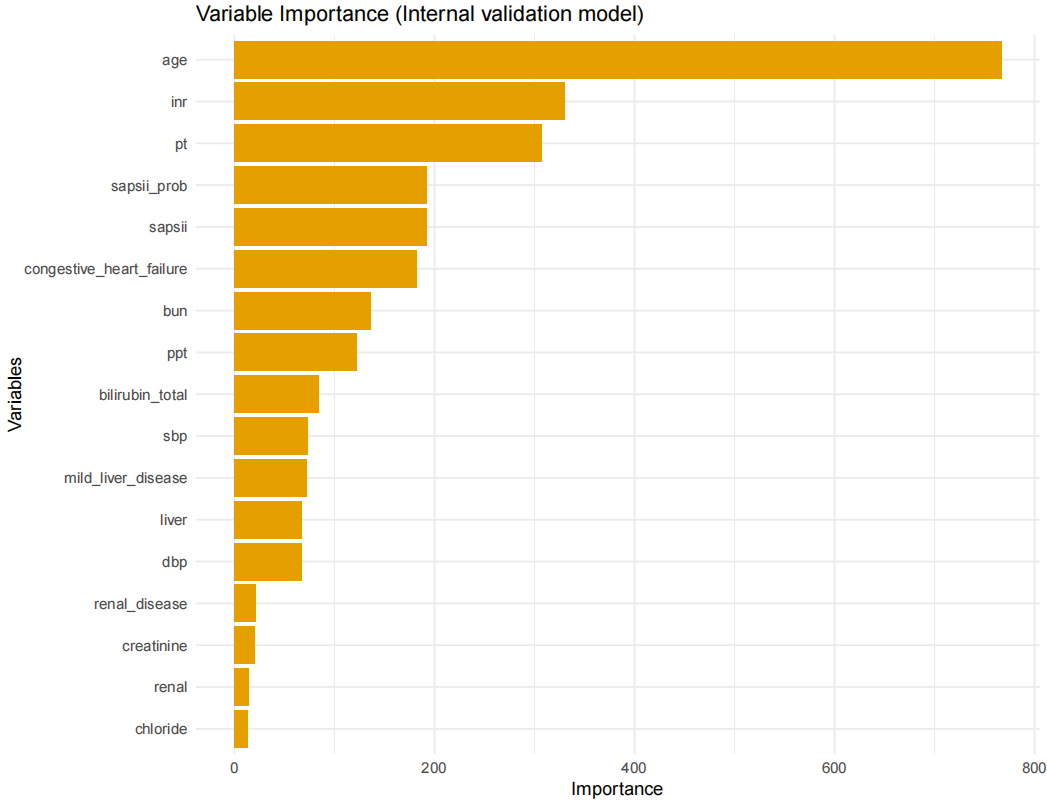


Figure. S9. Decision tree visualization and variable importance plots (External validation model).Abbreviations:Inr.International Normalized Ratio;Pt,Prothrombin Time;Sapsii,Simplified Acute Physiology Score II；Bun,Blood Urea Nitrogen;Ppt,Partial Thromboplastin Time;Sbp,Systolic Blood Pressure;Dbp,Diastolic Blood Pressure;oasis,Open Source Anonymized Simulator.


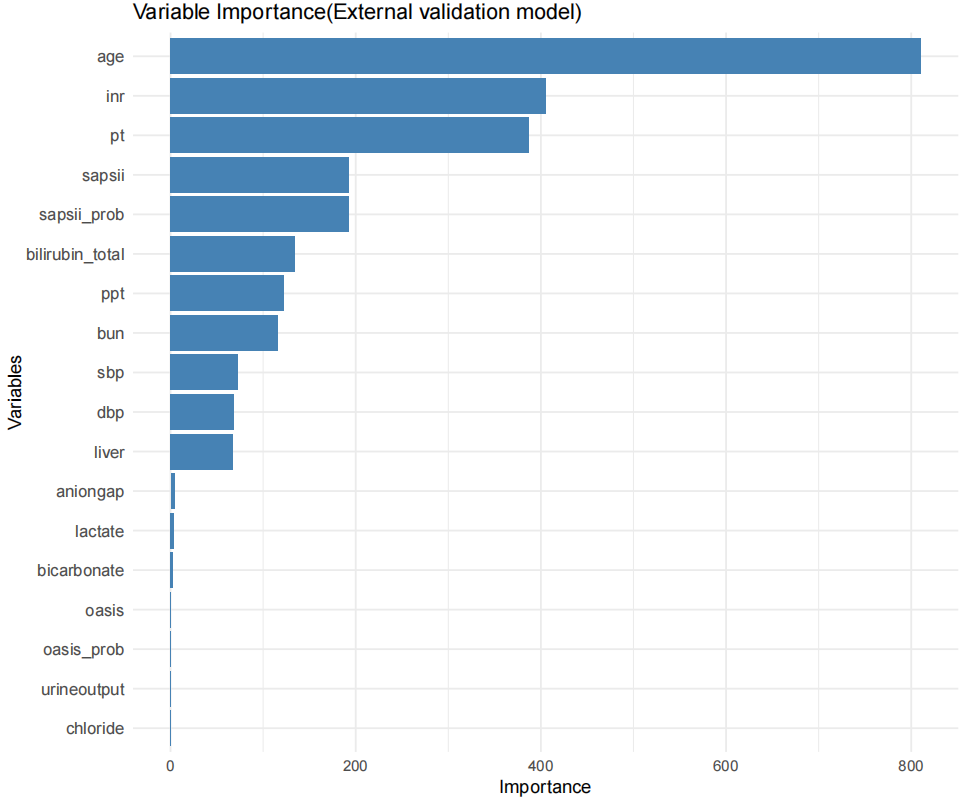


### Figure. S10. Support vector machine variable importance plot (Internal validation model and External validation model).

Abbreviations:Pt,Prothrombin Time;Inr.International Normalized Ratio;Ast,Aspartate Aminotransferase;SPo2,Peripheral Capillary Oxygen Saturation;Sbp,Systolic Blood Pressure;PH.Acidity;Sapsii,Simplified Acute Physiology Score II；Ppt,Partial Thromboplastin Time;oasis,Open Source Anonymized Simulator;Gcs Eyes,GCS Eye Opening;Mbp,Mean Blood Pressure;Bun,Blood Urea Nitrogen;Po2,Partial pressure of oxygen;Dbp,Diastolic Blood Pressure;Apsiii, Acute Physiology and Chronic Health Evaluation;Gcs,Glasgow Coma Scale.


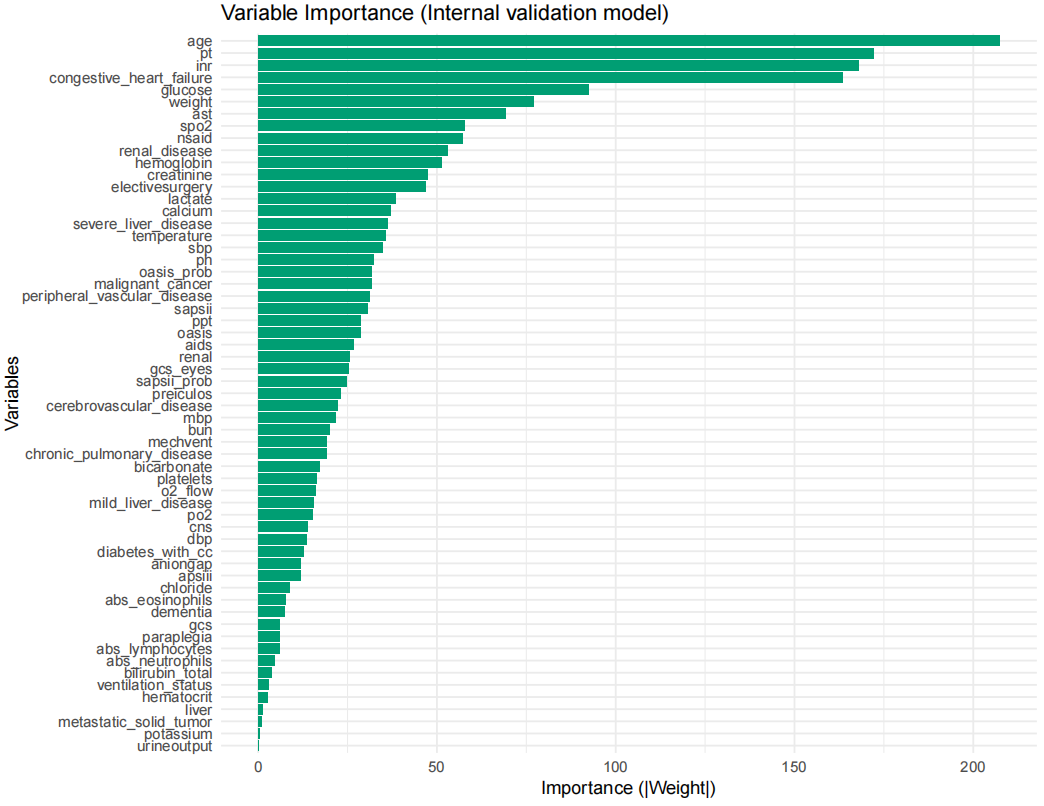


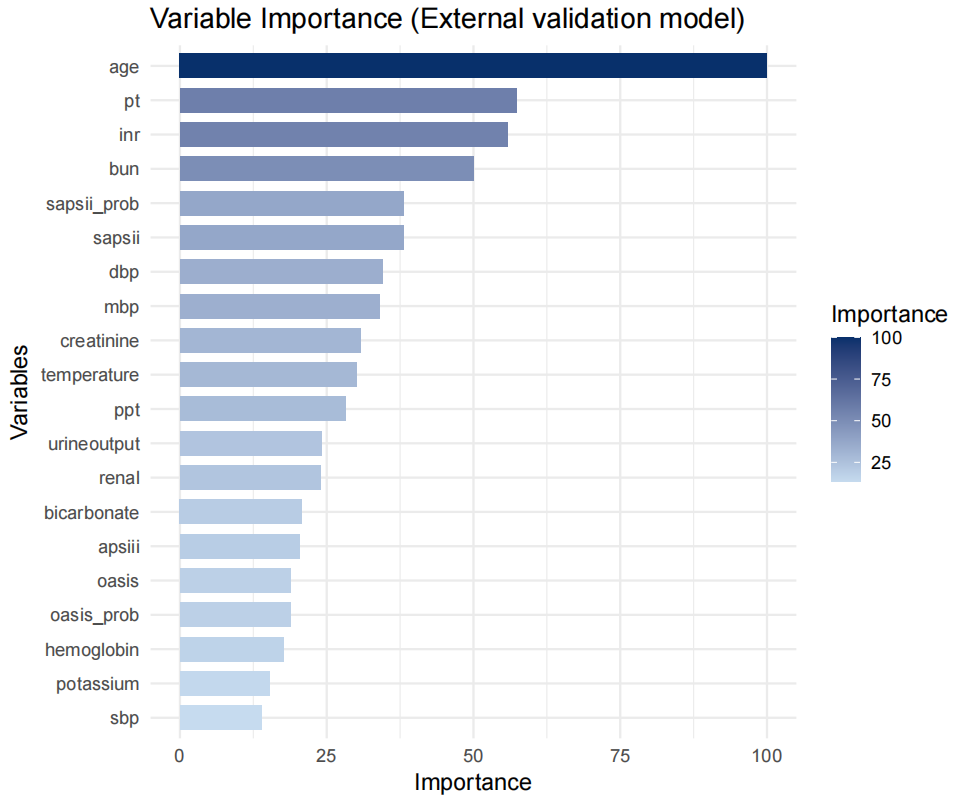


### Figure. S11. Artificial neural network topology and variable importance plots (Internal validation model).

Abbreviations:Ppt,Partial Thromboplastin Time;Sbp,Systolic Blood Pressure;Sapsii,Simplified Acute Physiology Score II；Pt,Prothrombin Time;SPo2,Peripheral Capillary Oxygen Saturation;Bun,Blood Urea Nitrogen;Po2,Partial pressure of oxygen;Gcs,Glasgow Coma Scale;Gcs Eyes,GCS Eye Opening;oasis,Open Source Anonymized Simulator;PH.Acidity;Ast,Aspartate Aminotransferase;Dbp,Diastolic Blood Pressure;Apsiii, Acute Physiology and Chronic Health Evaluation;Mbp,Mean Blood Pressure.


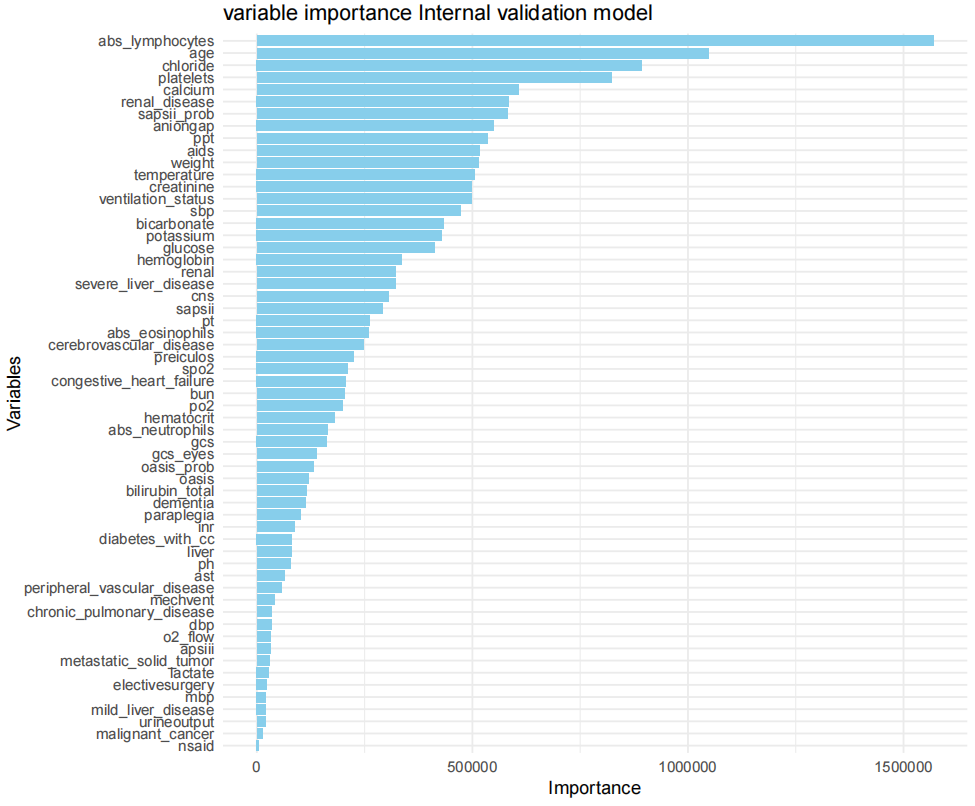


### Figure. S12. Artificial neural network topology and variable importance plots (External validation model).

Abbreviations:Ppt,Partial Thromboplastin Time;Sbp,Systolic Blood Pressure;Sapsii,Simplified Acute Physiology Score II；Pt,Prothrombin Time;SPo2,Peripheral Capillary Oxygen Saturation;Bun,Blood Urea Nitrogen;Po2,Partial pressure of oxygen;Gcs,Glasgow Coma Scale;Gcs Eyes,GCS Eye Opening;oasis,Open Source Anonymized Simulator;PH.Acidity;Ast,Aspartate Aminotransferase;Dbp,Diastolic Blood Pressure;Apsiii, Acute Physiology and Chronic Health Evaluation;Mbp,Mean Blood Pressure.


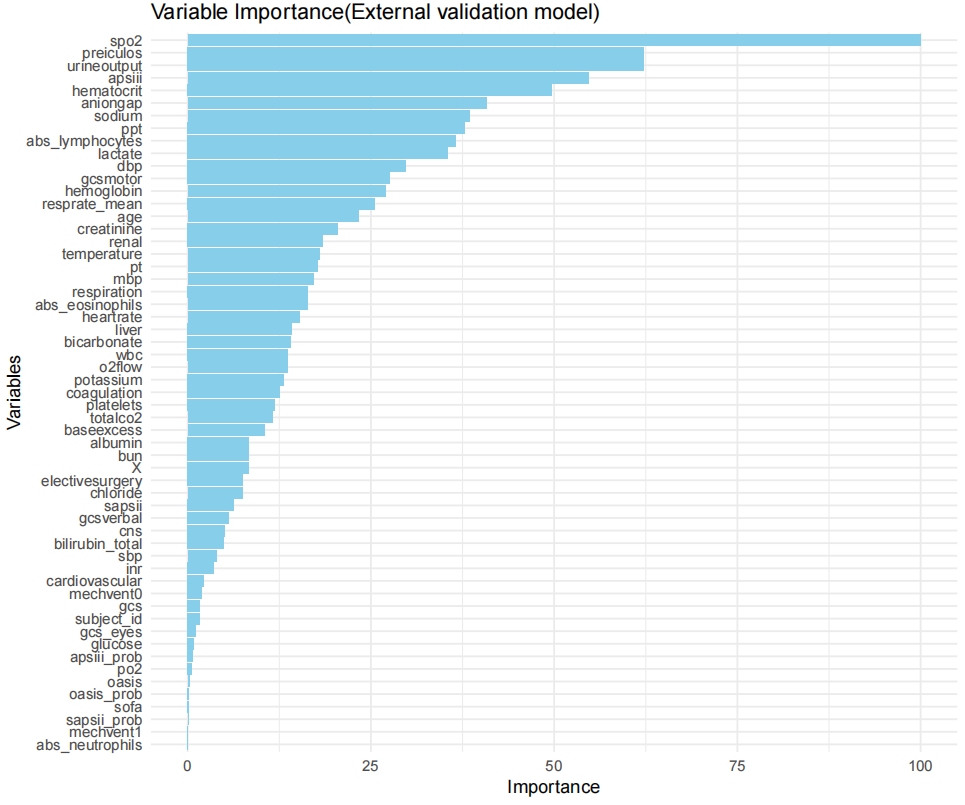


### Figure. S13. The static nomogram of the Internal validation model .

Abbreviations:Apsiii, Acute Physiology and Chronic Health Evaluation;Po2,Partial pressure of oxygen;Pco2,Partial pressure of carbon dioxide;PH.Acidity;Wbc,White blood cell count;Bun,Blood Urea Nitrogen;Inr.International Normalized Ratio;Pt,Prothrombin Time;Ppt,Partial Thromboplastin Time;Alt,Alanine Aminotransferase;Alp,Alkaline Phosphatase;Ast,Aspartate Aminotransferase;Sofa,Sequential Organ Failure Assessment;Sbp,Systolic Blood Pressure;Dbp,Diastolic Blood Pressure;Mbp,Mean Blood Pressure;Resp Rate,Respiratory Rate;SPo2,Peripheral Capillary Oxygen Saturation;Gcs,Glasgow Coma Scale;Gcs Motor,GCS Motor Response;Gcs Verbal,GCS Verbal Response;Gcs Eyes,GCS Eye Opening;Gcs Unable,GCS Unable to Score;oasis,Open Source Anonymized Simulator;Sapsii,Simplified Acute Physiology Score II.


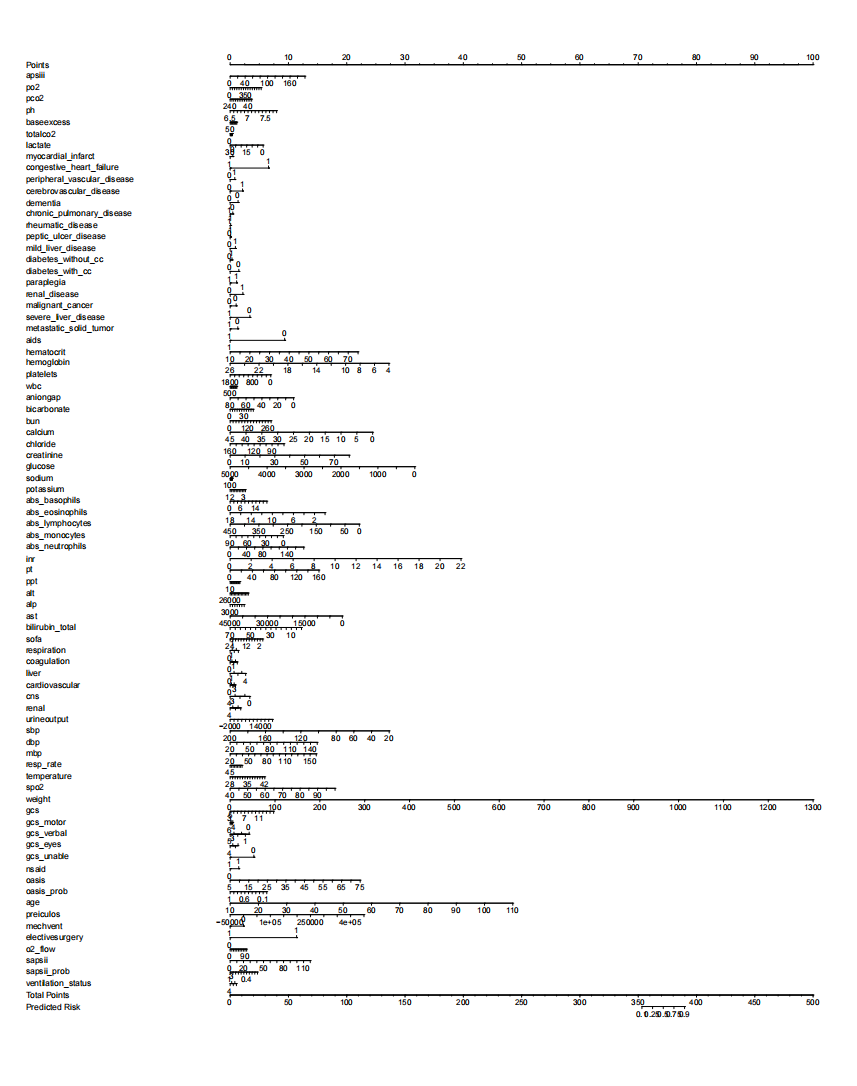


### Figure. S14. The static nomogram of the External validation model.

Abbreviations:Apsiii, Acute Physiology and Chronic Health Evaluation;Po2,Partial pressure of oxygen;Pco2,Partial pressure of carbon dioxide;PH.Acidity;Wbc,White blood cell count;Bun,Blood Urea Nitrogen;Inr.International Normalized Ratio;Pt,Prothrombin Time;Ppt,Partial Thromboplastin Time;Alt,Alanine Aminotransferase;Alp,Alkaline Phosphatase;Ast,Aspartate Aminotransferase;Sofa,Sequential Organ Failure Assessment;Sbp,Systolic Blood Pressure;Dbp,Diastolic Blood Pressure;Mbp,Mean Blood Pressure;Resp Rate,Respiratory Rate;SPo2,Peripheral Capillary Oxygen Saturation;Gcs,Glasgow Coma Scale;Gcs Motor,GCS Motor Response;Gcs Verbal,GCS Verbal Response;Gcs Eyes,GCS Eye Opening;Gcs Unable,GCS Unable to Score;oasis,Open Source Anonymized Simulator;Sapsii,Simplified Acute Physiology Score II.


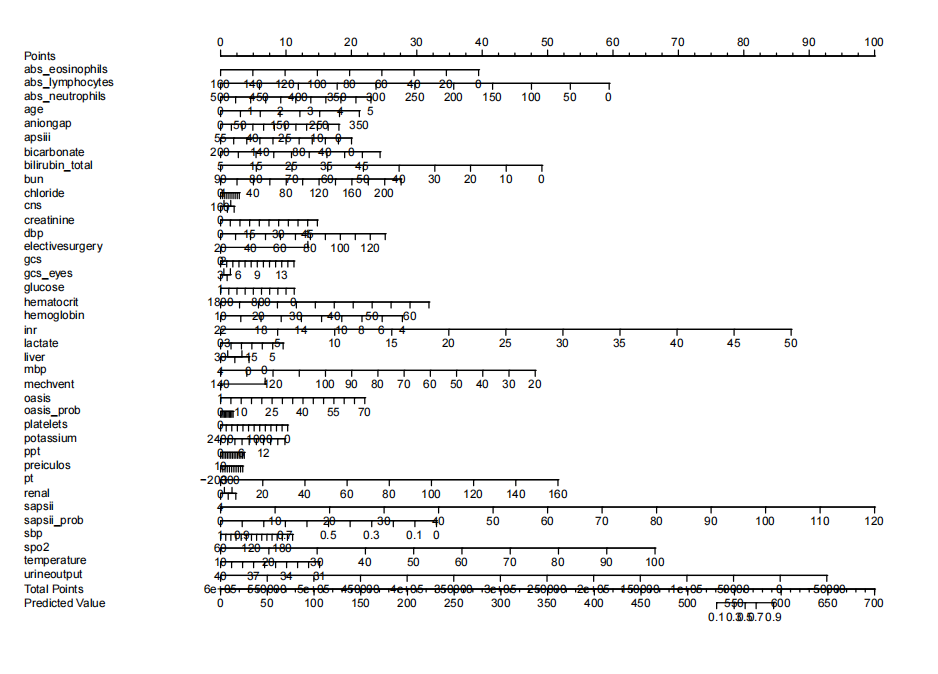


**Figure. S15. The online dynamic nomogram for the Internal validation model**

### For example, one woman was predicted to occur PPD in 87.2% probability (95%CI, 73.20%-94.40%).

**
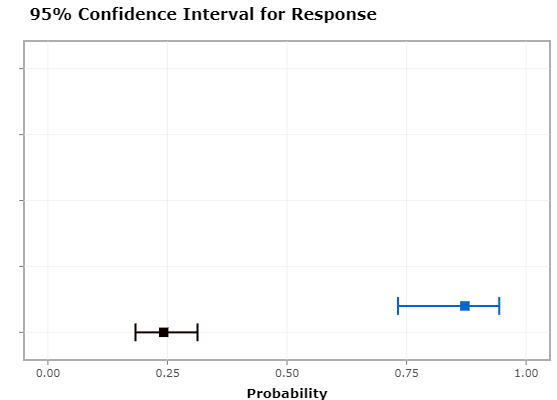
**

**Figure. S16. The online dynamic nomogram for the External validation model**

### For example, one woman was predicted to occur PPD in 85.8% probability (95%CI, 41.5%-98.1%).

**
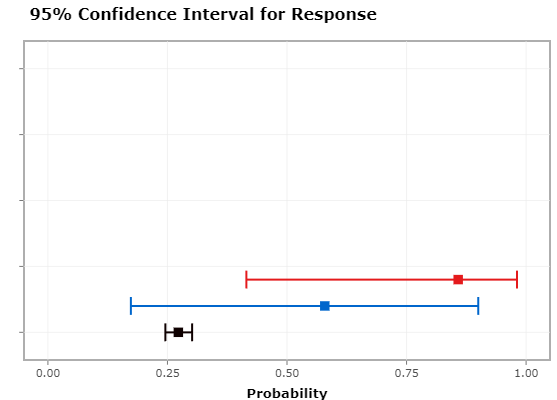
**

## Supplementary Hyperparameter Tuning Details

Six machine learning algorithms—logistic regression (LR), decision tree (DT), random forest (RF), extreme gradient boosting (XGBoost), support vector machine (SVM), and artificial neural network (ANN)—were trained on the training set. Hyperparameter optimization was performed via a systematic grid search with fivefold cross-validation to ensure generalizability. We used the RSNNS package for the ANN model, the caret package for the LR model, the rpart package for the DT model, the randomForest package for the RF model, the e1071 package for the SVM model, the xgboost package for the XGBoost model. Below are the parameter search spaces and final configurations.

The Internal validation model hyperparameters were the following:

1. XGBoost: objective="binary:logistic", validate_parameters = T
2. RF: ntree =100, mtry=6.
3. LR: family = binomial (), link function = logit
4. DT: minsplit = 20,minbucket = 7,cp = 0.01,maxcompete = 4,maxsurrogate = 5,usesurrogate = 2,surrogatestyle = 0,maxdepth = 30,xval = 10.
5. SVM: ernel ="radial", cost = 1, gamma=0.02631579.
6. ANN: size = 5, maxit = 200, trace = F.

The External validation model hyperparameters were the following:

1. XGBoost: objective="binary:logistic", validate_parameters = T
2. RF: ntree =100, mtry=6.
3. LR: family = binomial (), link function = logit
4. DT: minsplit = 20,minbucket = 7,cp = 0.01,maxcompete = 4,maxsurrogate = 5,usesurrogate = 2,surrogatestyle = 0,maxdepth = 30,xval = 10.
5. SVM: ernel ="radial", cost = 1, gamma=0.02631579.
6. ANN: size = 5, maxit = 200, trace = F.
