# Supplementary material for: Predicting atrial fibrillation in patients with acute respiratory failure using machine learning: application of the MIMIC-III and MIMIC-IV datasets
Source: Front Cardiovasc Med. 2025 Oct 9;12:1696609. doi: 10.3389/fcvm.2025.1696609 (PMC12546187; doi:10.3389/fcvm.2025.1696609)
Supplement: Supplementary file 4 [file Datasheet4.docx]

## Supplementary Tables

**Table 1**

Internal set

| Characteristic | Non-AF(n = 14372) | AF(n = 7222) | Statistic | *P* |
| --- | --- | --- | --- | --- |
|  |  |  |  |  |
| Age, M (Q₁, Q₃) | 64.30 (52.98, 75.09) | 76.17 (67.58, 84.06) | Z=-Inf | <.001 |
| ABG |  |  |  |  |
| Po2, M (Q₁, Q₃) | 79.00 (50.00, 140.00) | 77.00 (47.00, 142.00) | Z=-2.15 | 0.031 |
| Pco2, M (Q₁, Q₃) | 43.00 (36.00, 52.00) | 44.00 (37.00, 53.00) | Z=-3.87 | <.001 |
| Ph, M (Q₁, Q₃) | 7.36 (7.29, 7.42) | 7.36 (7.29, 7.42) | Z=-3.31 | <.001 |
| Baseexcess, M (Q₁, Q₃) | 0.00 (-4.00, 2.00) | 0.00 (-4.00, 2.00) | Z=-6.49 | <.001 |
| Totalco2, M (Q₁, Q₃) | 25.00 (21.00, 29.00) | 26.00 (22.00, 30.00) | Z=-6.52 | <.001 |
| Lactate, M (Q₁, Q₃) | 1.70 (1.20, 2.70) | 1.70 (1.20, 2.60) | Z=-0.60 | 0.549 |
| Spo2, M (Q₁, Q₃) | 96.78 (95.00, 98.42) | 96.64 (94.95, 98.21) | Z=-4.17 | <.001 |
| Lab |  |  |  |  |
| Hematocrit, M (Q₁, Q₃) | 34.50 (29.60, 39.70) | 33.50 (29.10, 38.40) | Z=-8.59 | <.001 |
| Hemoglobin, M (Q₁, Q₃) | 11.10 (9.40, 12.90) | 10.60 (9.10, 12.30) | Z=-12.74 | <.001 |
| Platelets, M (Q₁, Q₃) | 223.00 (158.00, 300.00) | 211.00 (152.00, 286.00) | Z=-5.89 | <.001 |
| Wbc, M (Q₁, Q₃) | 13.10 (9.30, 18.40) | 13.30 (9.50, 18.60) | Z=-2.55 | 0.011 |
| Aniongap, M (Q₁, Q₃) | 16.00 (13.00, 19.00) | 16.00 (14.00, 19.00) | Z=-2.12 | 0.034 |
| Bicarbonate, M (Q₁, Q₃) | 24.00 (21.00, 27.00) | 25.00 (22.00, 28.00) | Z=-5.65 | <.001 |
| Bun, M (Q₁, Q₃) | 23.00 (15.00, 39.00) | 32.00 (21.00, 51.00) | Z=-29.39 | <.001 |
| Calcium, M (Q₁, Q₃) | 8.70 (8.20, 9.10) | 8.60 (8.20, 9.10) | Z=-0.77 | 0.440 |
| Chloride, M (Q₁, Q₃) | 105.00 (100.00, 109.00) | 104.00 (99.00, 108.00) | Z=-8.69 | <.001 |
| Creatinine, M (Q₁, Q₃) | 1.10 (0.80, 1.90) | 1.40 (1.00, 2.30) | Z=-21.10 | <.001 |
| Glucose, M (Q₁, Q₃) | 154.00 (122.00, 209.00) | 156.00 (125.00, 209.00) | Z=-1.90 | 0.058 |
| Sodium, M (Q₁, Q₃) | 140.00 (137.00, 143.00) | 140.00 (137.00, 143.00) | Z=-1.09 | 0.276 |
| Potassium, M (Q₁, Q₃) | 4.50 (4.10, 5.10) | 4.60 (4.20, 5.20) | Z=-5.81 | <.001 |
| Abs Basophils, M (Q₁, Q₃) | 0.02 (0.00, 0.05) | 0.02 (0.00, 0.04) | Z=-2.31 | 0.021 |
| Abs Eosinophils, M (Q₁, Q₃) | 0.03 (0.00, 0.12) | 0.03 (0.00, 0.11) | Z=-2.92 | 0.004 |
| Abs Lymphocytes, M (Q₁, Q₃) | 1.09 (0.66, 1.72) | 0.98 (0.59, 1.56) | Z=-9.05 | <.001 |
| Abs Monocytes, M (Q₁, Q₃) | 0.64 (0.37, 0.99) | 0.67 (0.40, 1.03) | Z=-4.51 | <.001 |
| Abs Neutrophils, M (Q₁, Q₃) | 9.73 (6.37, 14.27) | 9.95 (6.71, 14.45) | Z=-3.34 | <.001 |
| Inr, M (Q₁, Q₃) | 1.30 (1.10, 1.60) | 1.50 (1.20, 2.10) | Z=-Inf | <.001 |
| Pt, M (Q₁, Q₃) | 14.00 (12.40, 17.10) | 16.40 (13.70, 23.20) | Z=-Inf | <.001 |
| Ppt, M (Q₁, Q₃) | 32.00 (28.00, 42.70) | 35.70 (30.00, 52.90) | Z=-21.88 | <.001 |
| Alt, M (Q₁, Q₃) | 28.00 (17.00, 62.00) | 26.00 (16.00, 60.00) | Z=-4.29 | <.001 |
| Alp, M (Q₁, Q₃) | 91.00 (67.00, 133.00) | 93.00 (68.00, 134.00) | Z=-2.65 | 0.008 |
| Ast, M (Q₁, Q₃) | 42.00 (24.00, 92.00) | 39.00 (24.00, 88.00) | Z=-2.78 | 0.006 |
| Bilirubin Total, M (Q₁, Q₃) | 0.60 (0.40, 1.10) | 0.70 (0.40, 1.10) | Z=-9.63 | <.001 |
|  |  |  |  |  |
|  |  |  |  |  |
| Vital Signs |  |  |  |  |
| Sbp, M (Q₁, Q₃) | 114.90 (105.35, 127.13) | 111.70 (103.67, 122.61) | Z=-12.63 | <.001 |
| Dbp, M (Q₁, Q₃) | 63.12 (56.55, 70.88) | 60.95 (54.77, 68.16) | Z=-14.04 | <.001 |
| Mbp, M (Q₁, Q₃) | 77.52 (71.19, 85.46) | 75.37 (69.68, 82.37) | Z=-14.11 | <.001 |
| Resp Rate, M (Q₁, Q₃) | 20.17 (17.56, 23.30) | 20.40 (17.91, 23.36) | Z=-3.98 | <.001 |
| Temperature, M (Q₁, Q₃) | 36.89 (36.64, 37.22) | 36.80 (36.56, 37.09) | Z=-14.66 | <.001 |
| Weight, M (Q₁, Q₃) | 78.00 (64.60, 95.30) | 79.80 (65.60, 96.80) | Z=-4.64 | <.001 |
| Scores |  |  |  |  |
| Gcs, M (Q₁, Q₃) | 15.00 (15.00, 15.00) | 15.00 (15.00, 15.00) | Z=-7.41 | <.001 |
| Gcs Motor, M (Q₁, Q₃) | 6.00 (4.00, 6.00) | 6.00 (4.00, 6.00) | Z=-3.94 | <.001 |
| Gcs Verbal, M (Q₁, Q₃) | 1.00 (0.00, 5.00) | 4.00 (0.00, 5.00) | Z=-5.52 | <.001 |
| Gcs Eyes, n(%) |  |  | χ²=19.40 | <.001 |
| 1 | 4190 (29.15) | 1969 (27.26) |  |  |
| 2 | 1238 (8.61) | 554 (7.67) |  |  |
| 3 | 1907 (13.27) | 952 (13.18) |  |  |
| 4 | 7037 (48.96) | 3747 (51.88) |  |  |
| Gcs Unable, n(%) |  |  | χ²=58.20 | <.001 |
| 0 | 7778 (54.12) | 4303 (59.58) |  |  |
| 1 | 6594 (45.88) | 2919 (40.42) |  |  |
| Oasis, M (Q₁, Q₃) | 34.00 (28.00, 40.00) | 36.00 (30.00, 43.00) | Z=-15.32 | <.001 |
| Oasis Prob, M (Q₁, Q₃) | 0.14 (0.07, 0.25) | 0.17 (0.09, 0.33) | Z=-15.32 | <.001 |
| Sofa, M (Q₁, Q₃) | 5.00 (3.00, 8.00) | 6.00 (3.25, 9.00) | Z=-10.55 | <.001 |
| Urineoutput, M (Q₁, Q₃) | 1380.00 (770.00, 2215.00) | 1185.00 (645.00, 2000.00) | Z=-11.29 | <.001 |
| Apsiii, M (Q₁, Q₃) | 46.00 (34.00, 63.00) | 52.00 (40.00, 68.00) | Z=-19.16 | <.001 |
| Sapsii, M (Q₁, Q₃) | 37.00 (28.00, 48.00) | 43.00 (35.00, 53.00) | Z=-28.92 | <.001 |
| Sapsii Prob, M (Q₁, Q₃) | 0.20 (0.09, 0.41) | 0.31 (0.17, 0.53) | Z=-28.92 | <.001 |
| Respiration, n(%) |  |  | χ²=53.43 | <.001 |
| 0 | 2514 (17.49) | 1047 (14.50) |  |  |
| 1 | 372 (2.59) | 168 (2.33) |  |  |
| 2 | 6281 (43.70) | 3462 (47.94) |  |  |
| 3 | 2661 (18.52) | 1228 (17.00) |  |  |
| 4 | 2544 (17.70) | 1317 (18.24) |  |  |
| Coagulation, n(%) |  |  | χ²=75.76 | <.001 |
| 0 | 9388 (65.32) | 4501 (62.32) |  |  |
| 1 | 2536 (17.65) | 1514 (20.96) |  |  |
| 2 | 1511 (10.51) | 873 (12.09) |  |  |
| 3 | 716 (4.98) | 247 (3.42) |  |  |
| 4 | 221 (1.54) | 87 (1.20) |  |  |
| Liver, n(%) |  |  | χ²=115.41 | <.001 |
| 0 | 11158 (77.64) | 5466 (75.69) |  |  |
| 1 | 1311 (9.12) | 919 (12.73) |  |  |
| 2 | 1195 (8.31) | 626 (8.67) |  |  |
| 3 | 374 (2.60) | 135 (1.87) |  |  |
| 4 | 334 (2.32) | 76 (1.05) |  |  |
| Cardiovascular, n(%) |  |  | χ²=232.27 | <.001 |
| 0 | 2661 (18.52) | 825 (11.42) |  |  |
| 1 | 7857 (54.67) | 4065 (56.29) |  |  |
| 2 | 43 (0.30) | 55 (0.76) |  |  |
| 3 | 837 (5.82) | 596 (8.25) |  |  |
| 4 | 2974 (20.69) | 1681 (23.28) |  |  |
| Cns, n(%) |  |  | χ²=49.40 | <.001 |
| 0 | 8900 (61.93) | 4161 (57.62) |  |  |
| 1 | 2738 (19.05) | 1626 (22.51) |  |  |
| 2 | 1016 (7.07) | 572 (7.92) |  |  |
| 3 | 1024 (7.12) | 535 (7.41) |  |  |
| 4 | 694 (4.83) | 328 (4.54) |  |  |
| Renal, n(%) |  |  | χ²=440.12 | <.001 |
| 0 | 6981 (48.57) | 2449 (33.91) |  |  |
| 1 | 3060 (21.29) | 1933 (26.77) |  |  |
| 2 | 1418 (9.87) | 1049 (14.53) |  |  |
| 3 | 1456 (10.13) | 951 (13.17) |  |  |
| 4 | 1457 (10.14) | 840 (11.63) |  |  |
| Charlson |  |  |  |  |
| Myocardial Infarct, n(%) |  |  | χ²=231.67 | <.001 |
| 0 | 12069 (83.98) | 5444 (75.38) |  |  |
| 1 | 2303 (16.02) | 1778 (24.62) |  |  |
| Congestive Heart Failure, n(%) |  |  | χ²=1995.62 | <.001 |
| 0 | 10088 (70.19) | 2786 (38.58) |  |  |
| 1 | 4284 (29.81) | 4436 (61.42) |  |  |
| Peripheral Vascular Disease, n(%) |  |  | χ²=175.17 | <.001 |
| 0 | 13041 (90.74) | 6117 (84.70) |  |  |
| 1 | 1331 (9.26) | 1105 (15.30) |  |  |
| Cerebrovascular Disease, n(%) |  |  | χ²=29.09 | <.001 |
| 0 | 12244 (85.19) | 5948 (82.36) |  |  |
| 1 | 2128 (14.81) | 1274 (17.64) |  |  |
| Dementia, n(%) |  |  | χ²=73.03 | <.001 |
| 0 | 13660 (95.05) | 6654 (92.14) |  |  |
| 1 | 712 (4.95) | 568 (7.86) |  |  |
| Chronic Pulmonary Disease, n(%) |  |  | χ²=20.45 | <.001 |
| 0 | 9365 (65.16) | 4480 (62.03) |  |  |
| 1 | 5007 (34.84) | 2742 (37.97) |  |  |
| Rheumatic Disease, n(%) |  |  | χ²=4.08 | 0.043 |
| 0 | 13879 (96.57) | 6935 (96.03) |  |  |
| 1 | 493 (3.43) | 287 (3.97) |  |  |
| Peptic Ulcer Disease, n(%) |  |  | χ²=1.16 | 0.282 |
| 0 | 13981 (97.28) | 7007 (97.02) |  |  |
| 1 | 391 (2.72) | 215 (2.98) |  |  |
| Mild Liver Disease, n(%) |  |  | χ²=79.02 | <.001 |
| 0 | 12117 (84.31) | 6412 (88.78) |  |  |
| 1 | 2255 (15.69) | 810 (11.22) |  |  |
| Diabetes Without Cc, n(%) |  |  | χ²=14.64 | <.001 |
| 0 | 10935 (76.09) | 5323 (73.71) |  |  |
| 1 | 3437 (23.91) | 1899 (26.29) |  |  |
| Diabetes With Cc, n(%) |  |  | χ²=90.90 | <.001 |
| 0 | 12512 (87.06) | 5937 (82.21) |  |  |
| 1 | 1860 (12.94) | 1285 (17.79) |  |  |
| Paraplegia, n(%) |  |  | χ²=3.19 | 0.074 |
| 0 | 13453 (93.61) | 6805 (94.23) |  |  |
| 1 | 919 (6.39) | 417 (5.77) |  |  |
| Renal Disease, n(%) |  |  | χ²=653.80 | <.001 |
| 0 | 11174 (77.75) | 4422 (61.23) |  |  |
| 1 | 3198 (22.25) | 2800 (38.77) |  |  |
| Malignant Cancer, n(%) |  |  | χ²=11.66 | <.001 |
| 0 | 11983 (83.38) | 6152 (85.18) |  |  |
| 1 | 2389 (16.62) | 1070 (14.82) |  |  |
| Severe Liver Disease, n(%) |  |  | χ²=117.44 | <.001 |
| 0 | 13187 (91.75) | 6913 (95.72) |  |  |
| 1 | 1185 (8.25) | 309 (4.28) |  |  |
| Metastatic Solid Tumor, n(%) |  |  | χ²=33.20 | <.001 |
| 0 | 13183 (91.73) | 6783 (93.92) |  |  |
| 1 | 1189 (8.27) | 439 (6.08) |  |  |
| Aids, n(%) |  |  | χ²=57.59 | <.001 |
| 0 | 14198 (98.79) | 7208 (99.81) |  |  |
| 1 | 174 (1.21) | 14 (0.19) |  |  |
| Intervention/Status |  |  |  |  |
| Preiculos, M (Q₁, Q₃) | 106.00 (47.00, 1218.51) | 125.00 (51.00, 2338.21) | Z=-6.56 | <.001 |
| O2 Flow, M (Q₁, Q₃) | 8.00 (4.00, 10.00) | 6.00 (3.00, 10.00) | Z=-2.51 | 0.012 |
| Nsaid, n(%) |  |  | χ²=340.97 | <.001 |
| 0 | 8331 (57.97) | 3227 (44.68) |  |  |
| 1 | 6041 (42.03) | 3995 (55.32) |  |  |
| Mechvent, n(%) |  |  | χ²=47.43 | <.001 |
| 0 | 6105 (42.48) | 3424 (47.41) |  |  |
| 1 | 8267 (57.52) | 3798 (52.59) |  |  |
| Electivesurgery, n(%) |  |  | χ²=76.92 | <.001 |
| 0 | 14294 (99.46) | 7094 (98.23) |  |  |
| 1 | 78 (0.54) | 128 (1.77) |  |  |
| Ventilation Status, n(%) |  |  | χ²=88.31 | <.001 |
| 0 | 745 (5.18) | 325 (4.50) |  |  |
| 1 | 4880 (33.95) | 2872 (39.77) |  |  |
| 2 | 480 (3.34) | 271 (3.75) |  |  |
| 3 | 406 (2.82) | 237 (3.28) |  |  |
| 4 | 7861 (54.70) | 3517 (48.70) |  |  |

**Table 2**

**External set**

| Variables | Non-AF (n = 4274) | AF (n = 1802) | Statistic | *P* |
| --- | --- | --- | --- | --- |
|  |  |  |  |  |
| Age, M (Q₁, Q₃) | 61.37 (49.39, 74.51) | 75.55 (65.44, 83.06) | Z=-26.87 | **<.001** |
| **ABG** |  |  |  |  |
| Po2, M (Q₁, Q₃) | 100.00 (69.00, 189.00) | 101.00 (68.00, 182.00) | Z=-0.33 | 0.740 |
| Lactate, M (Q₁, Q₃) | 2.10 (1.40, 3.70) | 2.10 (1.40, 3.40) | Z=-1.75 | 0.081 |
|  |  |  |  |  |
| Totalco2, M (Q₁, Q₃) | 25.00 (21.00, 29.00) | 26.00 (22.00, 31.00) | Z=-6.35 | **<.001** |
| Spo2, M (Q₁, Q₃) | 97.38 (95.67, 98.74) | 97.33 (95.74, 98.65) | Z=-0.63 | 0.528 |
| **LAB** |  |  |  |  |
| Hematocrit, M (Q₁, Q₃) | 34.70 (30.60, 39.40) | 34.00 (30.33, 38.30) | Z=-3.12 | **0.002** |
| Hemoglobin, M (Q₁, Q₃) | 11.50 (10.10, 13.20) | 11.20 (9.90, 12.70) | Z=-4.87 | **<.001** |
| Platelets, M (Q₁, Q₃) | 235.00 (159.25, 321.00) | 232.00 (170.00, 313.00) | Z=-0.83 | 0.406 |
| Wbc, M (Q₁, Q₃) | 13.00 (9.00, 18.20) | 13.20 (9.30, 18.20) | Z=-1.13 | 0.260 |
| Aniongap, M (Q₁, Q₃) | 16.00 (14.00, 19.00) | 16.00 (14.00, 19.00) | Z=-0.93 | 0.352 |
| Bicarbonate,M (Q₁, Q₃) | 22.00 (18.00, 25.00) | 23.00 (19.00, 26.00) | Z=-6.19 | **<.001** |
| Bun, M (Q₁, Q₃) | 24.00 (16.00, 41.00) | 32.00 (21.00, 50.00) | Z=-13.44 | **<.001** |
| Chloride, M (Q₁, Q₃) | 107.00 (103.00, 111.00) | 106.00 (102.00, 111.00) | Z=-3.78 | **<.001** |
| Creatinine, M (Q₁, Q₃) | 1.10 (0.80, 2.00) | 1.40 (0.90, 2.20) | Z=-8.34 | **<.001** |
| Glucose, M (Q₁, Q₃) | 159.00 (126.00, 214.00) | 162.00 (128.00, 211.00) | Z=-0.94 | 0.346 |
| Sodium, M (Q₁, Q₃) | 141.00 (138.00, 143.00) | 141.00 (138.00, 143.75) | Z=-0.76 | 0.450 |
| Potassium, M (Q₁, Q₃) | 4.50 (4.10, 5.10) | 4.60 (4.10, 5.20) | Z=-4.59 | **<.001** |
| Abs Eosinophils, M (Q₁, Q₃) | 0.08 (0.01, 0.23) | 0.08 (0.01, 0.24) | Z=-1.14 | 0.256 |
| Abs Lymphocytes, M (Q₁, Q₃) | 1.31 (0.71, 2.27) | 1.23 (0.68, 2.07) | Z=-2.75 | **0.006** |
| Abs Neutrophils, M (Q₁, Q₃) | 0.13 (0.09, 0.18) | 0.13 (0.09, 0.18) | Z=-1.13 | 0.260 |
|  |  |  |  |  |
| Inr, M (Q₁, Q₃) | 1.30 (1.10, 1.60) | 1.50 (1.20, 2.20) | Z=-15.36 | **<.001** |
| Pt, M (Q₁, Q₃) | 14.40 (13.20, 16.90) | 15.85 (13.90, 21.30) | Z=-15.52 | **<.001** |
| Ppt, M (Q₁, Q₃) | 32.60 (27.50, 44.48) | 35.40 (29.20, 49.30) | Z=-7.72 | **<.001** |
| Bilirubin Total, M (Q₁, Q₃) | 0.60 (0.40, 1.20) | 0.70 (0.40, 1.20) | Z=-1.95 | 0.051 |
| **Vital signs** |  |  |  |  |
| Sbp, M (Q₁, Q₃) | 115.22 (105.07, 128.58) | 113.00 (104.51, 124.99) | Z=-3.68 | **<.001** |
| Dbp, M (Q₁, Q₃) | 60.38 (53.68, 67.88) | 57.76 (51.33, 64.75) | Z=-9.13 | **<.001** |
| Mbp, M (Q₁, Q₃) | 76.83 (69.85, 85.03) | 73.78 (67.83, 81.28) | Z=-9.13 | **<.001** |
| Resp Rate, M (Q₁, Q₃) | 19.74 (16.87, 23.33) | 19.90 (17.25, 22.85) | Z=-0.85 | 0.398 |
| Temperature, M (Q₁, Q₃) | 36.90 (36.45, 37.39) | 36.73 (36.32, 37.20) | Z=-8.05 | **<.001** |
| **Scores** |  |  |  |  |
| Gcs, M (Q₁, Q₃) | 15.00 (14.00, 15.00) | 15.00 (13.00, 15.00) | Z=-1.65 | 0.099 |
| Gcsmotor, M (Q₁, Q₃) | 6.00 (4.00, 6.00) | 6.00 (5.00, 6.00) | Z=-1.90 | 0.057 |
| Gcsverbal, M (Q₁, Q₃) | 1.00 (0.00, 5.00) | 1.00 (0.00, 5.00) | Z=-3.06 | **0.002** |
| Gcs Eyes, n(%) |  |  | χ²=6.58 | 0.087 |
| 1 | 1076 (25.18) | 409 (22.70) |  |  |
| 2 | 472 (11.04) | 188 (10.43) |  |  |
| 3 | 1111 (25.99) | 470 (26.08) |  |  |
| 4 | 1615 (37.79) | 735 (40.79) |  |  |
|  |  |  |  |  |
| Oasis, M (Q₁, Q₃) | 36.00 (30.00, 43.00) | 38.00 (32.00, 44.00) | Z=-5.47 | **<.001** |
| Oasis Prob, M (Q₁, Q₃) | 0.17 (0.09, 0.33) | 0.21 (0.11, 0.36) | Z=-5.47 | **<.001** |
| Sofa, M (Q₁, Q₃) | 5.00 (3.00, 8.00) | 5.00 (3.00, 8.00) | Z=-0.76 | 0.449 |
| Urineoutput, M (Q₁, Q₃) | 1530.00 (870.00, 2425.00) | 1310.00 (740.00, 2058.75) | Z=-6.84 | **<.001** |
| Apsiii, M (Q₁, Q₃) | 49.00 (35.00, 66.00) | 53.00 (40.00, 68.00) | Z=-5.42 | **<.001** |
| Apsiii Prob, M (Q₁, Q₃) | 0.11 (0.06, 0.21) | 0.13 (0.07, 0.23) | Z=-5.42 | **<.001** |
| Sapsii, M (Q₁, Q₃) | 40.00 (29.00, 51.00) | 44.00 (36.00, 54.00) | Z=-10.06 | **<.001** |
| Sapsii Prob, M (Q₁, Q₃) | 0.25 (0.10, 0.48) | 0.33 (0.18, 0.55) | Z=-10.06 | **<.001** |
| Respiration, n(%) |  |  | χ²=25.38 | **<.001** |
| 0 | 1511 (35.35) | 681 (37.79) |  |  |
| 1 | 181 (4.23) | 75 (4.16) |  |  |
| 2 | 933 (21.83) | 429 (23.81) |  |  |
| 3 | 1090 (25.50) | 461 (25.58) |  |  |
| 4 | 559 (13.08) | 156 (8.66) |  |  |
| Coagulation, n (%) |  |  | χ²=48.07 | **<.001** |
| 0 | 2881 (67.41) | 1222 (67.81) |  |  |
| 1 | 599 (14.01) | 345 (19.15) |  |  |
| 2 | 487 (11.39) | 161 (8.93) |  |  |
| 3 | 231 (5.40) | 58 (3.22) |  |  |
| 4 | 76 (1.78) | 16 (0.89) |  |  |
| Liver, n(%) |  |  | χ²=43.33 | **<.001** |
| 0 | 3207 (75.04) | 1394 (77.36) |  |  |
| 1 | 324 (7.58) | 195 (10.82) |  |  |
| 2 | 462 (10.81) | 144 (7.99) |  |  |
| 3 | 138 (3.23) | 36 (2.00) |  |  |
| 4 | 143 (3.35) | 33 (1.83) |  |  |
| Cardiovascular, n(%) |  |  | χ²=47.00 | **<.001** |
| 0 | 683 (15.98) | 174 (9.66) |  |  |
| 1 | 2431 (56.88) | 1078 (59.82) |  |  |
| 2 | 68 (1.59) | 31 (1.72) |  |  |
| 3 | 314 (7.35) | 174 (9.66) |  |  |
| 4 | 778 (18.20) | 345 (19.15) |  |  |
| Cns, n(%) |  |  | χ²=12.59 | **0.013** |
| 0 | 2700 (63.17) | 1087 (60.32) |  |  |
| 1 | 730 (17.08) | 344 (19.09) |  |  |
| 2 | 289 (6.76) | 147 (8.16) |  |  |
| 3 | 345 (8.07) | 157 (8.71) |  |  |
| 4 | 210 (4.91) | 67 (3.72) |  |  |
| Renal, n(%) |  |  | χ²=69.58 | **<.001** |
| 0 | 2071 (48.46) | 671 (37.24) |  |  |
| 1 | 956 (22.37) | 495 (27.47) |  |  |
| 2 | 459 (10.74) | 267 (14.82) |  |  |
| 3 | 399 (9.34) | 187 (10.38) |  |  |
| 4 | 389 (9.10) | 182 (10.10) |  |  |
| **Other** |  |  |  |  |
| Preiculos, M (Q₁, Q₃) | 1.86 (1.22, 1032.95) | 1.89 (1.27, 1817.11) | Z=-2.20 | **0.028** |
| Baseexcess, M (Q₁, Q₃) | 0.00 (-5.00, 2.00) | 0.00 (-4.00, 3.00) | Z=-6.76 | **<.001** |
| O2flow, M (Q₁, Q₃) | 4.00 (2.00, 6.00) | 4.00 (2.00, 9.00) | Z=-1.26 | 0.207 |
| Electivesurgery, n(%) |  |  | χ²=7.13 | **0.008** |
| 0 | 4137 (96.79) | 1719 (95.39) |  |  |
| 1 | 137 (3.21) | 83 (4.61) |  |  |
| Mechvent, n(%) |  |  | χ²=22.92 | **<.001** |
| 0 | 994 (23.26) | 524 (29.08) |  |  |
| 1 | 3280 (76.74) | 1278 (70.92) |  |  |

**Table 3**

Predictor variables for the internal set obtained using six selection methods

| **Methods** | **Predictor variables** |
| --- | --- |
| LASSO | apsiii, po2, pco2, ph, totalco2, lactate, myocardial_infarct, congestive_heart_failure,  peripheral_vascular_disease, cerebrovascular_disease, dementia, chronic_pulmonary_disease, rheumatic_disease,  mild_liver_disease, diabetes_without_cc, diabetes_with_cc, paraplegia, renal_disease, malignant_cancer,  severe_liver_disease, metastatic_solid_tumor, aids, hematocrit, hemoglobin, platelets, wbc, aniongap,  bicarbonate, bun, calcium, chloride, creatinine, glucose, sodium, potassium, abs_basophils,  abs_eosinophils, abs_lymphocytes, abs_monocytes, abs_neutrophils, inr, pt, ppt, alt, alp,  ast, bilirubin_total, sofa, respiration, coagulation, liver, cardiovascular, cns, renal,  urineoutput, sbp, dbp, mbp, resp_rate, temperature, spo2, weight, gcs, gcs_motor,  gcs_verbal, gcs_eyes, gcs_unable, nsaid, oasis, oasis_prob, age, preiculos, mechvent,  electivesurgery, o2_flow, sapsii, sapsii_prob, ventilation_status |
| RF-MDA | age, congestive_heart_failure, pt, inr, sapsii_prob, sapsii, bun, ppt, apsiii, bilirubin_total, creatinine, sofa, oasis, nsaid, mbp, sbp, oasis_prob, ast, totalco2, dbp, renal_disease, baseexcess, severe_liver_disease, platelets, myocardial_infarct, bicarbonate, hemoglobin, lactate, liver, hematocrit |
| RF-MDG | age, congestive_heart_failure, pt, inr, sapsii_prob, sapsii, bun, ppt, apsiii, bilirubin_total, creatinine, sofa, oasis, nsaid, mbp, sbp, oasis_prob, ast, totalco2, dbp, renal_disease, baseexcess, severe_liver_disease, platelets, myocardial_infarct, bicarbonate, hemoglobin, lactate, liver, hematocrit |
| SR-FS | age, congestive_heart_failure, inr, weight, sbp, mbp, electivesurgery, ph, renal_disease, oasis, nsaid, hemoglobin, cerebrovascular_disease, glucose, preiculos, severe_liver_disease, spo2, mechvent, gcs, po2, bicarbonate, bun, calcium, aids, ast, apsiii, cns, renal, pt, diabetes_with_cc, malignant_cancer, platelets, hematocrit, sodium, abs_neutrophils, aniongap, ppt, dbp, abs_eosinophils, creatinine, dementia, oasis_prob, urineoutput, abs_lymphocytes, gcs_eyes, temperature, chloride, peripheral_vascular_disease, mild_liver_disease, bilirubin_total, lactate, paraplegia, liver, metastatic_solid_tumor, o2_flow, ventilation_status, chronic_pulmonary_disease, sapsii, potassium, sapsii_prob |
| SR-BS | apsiii, po2, ph, lactate, congestive_heart_failure, peripheral_vascular_disease, cerebrovascular_disease, dementia, chronic_pulmonary_disease, mild_liver_disease, diabetes_with_cc, paraplegia, renal_disease, malignant_cancer, severe_liver_disease, metastatic_solid_tumor, aids, hematocrit, hemoglobin, platelets, aniongap, bicarbonate, bun, calcium, chloride, creatinine, glucose, potassium, abs_eosinophils, abs_lymphocytes, abs_neutrophils, inr, pt, ppt, ast, bilirubin_total, liver, cns, renal, urineoutput, sbp, dbp, mbp, temperature, spo2, weight, gcs, gcs_verbal, gcs_eyes, gcs_unable, nsaid, oasis, oasis_prob, age, preiculos, mechvent, electivesurgery, o2_flow, sapsii, sapsii_prob, ventilation_status |
| SR-BE | abs_eosinophils, abs_lymphocytes, abs_neutrophils, age, aids, aniongap, apsiii, ast, bicarbonate, bilirubin_total, bun, calcium, cerebrovascular_disease, chloride, chronic_pulmonary_disease, cns, congestive_heart_failure, creatinine, dbp, dementia, diabetes_with_cc, electivesurgery, gcs, gcs_eyes, glucose, hematocrit, hemoglobin, inr, lactate, liver, malignant_cancer, mbp, mechvent, metastatic_solid_tumor, mild_liver_disease, nsaid, o2_flow, oasis, oasis_prob, paraplegia, peripheral_vascular_disease, ph, platelets, po2, potassium, ppt, preiculos, pt, renal, renal_disease, sapsii, sapsii_prob, sbp, severe_liver_disease, spo2, temperature, urineoutput, ventilation_status, weight |

**Table 4**

Predictive performance metrics of different machine learning algorithms of the validation set

| **Models** | **XGBOOST** | **RF** | **LR** | **DT** | **SVM** | **ANN** |
| --- | --- | --- | --- | --- | --- | --- |
| Internal validation set | | | | | | |
| AUC (95%CI) | 0.816 [ 0.804 - 0.829 ] | 0.81 [ 0.796 - 0.823 ] | 0.802 [0.789 - 0.816 ] | 0.734 [ 0.719 - 0.75 ] | 0.806 [ 0.793 - 0.819 ] | 0.759 [ 0.744 - 0.774 ]0.779 |
| Cutoff value | 0.27 | 0.32 | 0.33 | 0.17 | 0.01 | 0.01 |
| SEN (95%CI) | 0.626 (0.601 - 0.651) | 0.673 ( 0.648 - 0.697) | 0.642 (0.618 - 0.667) | 0.512 (0.486 - 0.538) | 0.597 (0.572 - 0.622) | 0.649 (95% CI: 0.624 - 0.673) |
| SPE (95%CI) | 0.800 (0.785 - 0.815) | 0.786 (0.771 - 0.810) | 0.787 0.772 - 0.801) | 0.840 (0.826 - 0.853) | 0.827 ( 0.813 - 0.841) | 0.666 (0.649 - 0.684) |
| PLR (95%CI) | 3.133 ( 2.882 - 3.405) | 3.148 (3.040 - 3.256) | 3.009 (2.778 - 3.260) | 3.194 (2.897 - 3.521) | 3.447 (3.149 - 3.773) | 1.944 (1.824 - 2.073) |
| NLR (95%CI) | 0.467 (0.436 - 0.501) | 0.416 (0.378 - 0.450) | 0.455 (0.423 - 0.489) | 0.581 (0.550 - 0.614) | 0.487 (0.457 - 0.520) | 0.527 (0.489 - 0.568) |
| PPV (95%CI) | 0.610 (0.585 - 0.635) | 0.611 (0.596 - 0.646) | 0.600 (0.587 - 0.635) | 0.614 (0.587 - 0.642) | 0.632 ( 0.607 - 0.658) | 0.492 ( 0.470 - 0.515) |
| NPV (95%CI) | 0.811 ( 0.797 - 0.825) | 0.828(0.813-0.842) | 0.815 (0.813 - 0.842) | 0.775 (0.761 - 0.790) | 0.804 (0.790 - 0.819) | 0.792 (0.776 - 0.808) |
| F1 score | 0.6267 | 0.6332 | 0.627 | 0.4685 | 0.5571 | 0.6811 |
| External validation set | | | | | | |
| AUC (95%CI) | 0.771:0.758 - 0.784) | 0.822（0.811 - 0.834） | 0.742（0.729- 0.755） | 0.685（0.671 - 0.697） | 0.750(0.737- 0.764） | 0.739（0.725-0.752） |
| Cutoff value | 0.17 | 0.36 | 0.26 | 0.28 | 0.66 | 0.229 |
| SEN (95%CI) | 0.704(0.684-0.725) | 0.748(0.  728-0.766) | 0.752(0.734-0.771) | 0.644(0.621-0.667) | 0.691(0.651-0.729) | 0.658(0.636-0.677) |
| SPE (95%CI) | 0.702(0.689-0.716) | 0.741(0.727-0.754) | 0.620(0.606-0.634) | 0.677(0.663-0.690) | 0.744(0.732--0.755) | 0.700(0.687-0.713) |
| PLR (95%CI) | 2.366(2.247-2.501) | 2.889(2.718-3.057) | 1.981(1.900-2.074) | 1.993(1.889-2.110) | 2.696(2.657-2.718) | 2.191(2.071-2.313) |
| NLR (95%CI) | 0.421(0.390-0.452) | 0.341(0.315-0.368) | 0.400(0.367-0.431) | 0.526(0.491-0.561) | 0.416(0.359-0.477) | 0.489(0.458-0.521) |
| PPV (95%CI) | 0.499(0.478-0.520) | 0.549(0.529-0.569) | 0.455(0.439-0.472) | 0.457(0.438-0.475) | 0.216(0.197-0.236) | 0.480(0.460-0.500) |
| NPV (95%CI) | 0.849(0.838-0.860) | 0.874(0.864-0.884) | 0.856(0.844-0.867) | 0.818(0.806-0.831) | 0.959(0.953-0.965) | 0.829(0.817-0.840) |
| F1 score | 0.58 | 0.63 | 0.57 | 0.53 | 0.22 | 0.56 |

### Table S1. Summary of missing data.

| Variables | Number | (%) |
| --- | --- | --- |
| Po2 | 1839 | 8.5 |
| Pco2 | 1890 | 8.7 |
| PH | 1847 | 8.5 |
| BE | 1892 | 8.7 |
| Total_co2 | 1891 | 8.7 |
| Lactate | 3710 | 17.1 |
| SPo2 | 66 | 0.3 |
| Hematocrit | 220 | 1 |
| Hemoglobin | 226 | 1 |
| Platelets | 226 | 1 |
| Wbc | 227 | 1 |
| Aniongap | 236 | 1 |
| Bicarbonate | 233 | 1 |
| Bun | 205 | 0.9 |
| Calcium | 647 | 2.9 |
| Chloride | 230 | 1 |
| Creatinine | 210 | 0.9 |
| Glucose | 248 | 1.1 |
| Sodium | 229 | 1 |
| Potassium | 232 | 1 |
| Abs Basophils | 3477 | 16 |
| Abs Eosinophils | 3477 | 16 |
| Abs Lymphocytes | 3473 | 16 |
| Abs Monocytes | 3477 | 16 |
| Abs Neutrophils | 3477 | 16 |
| Inr | 2063 | 9.4 |
| Pt | 2059 | 9.4 |
| Ppt | 2185 | 10 |
| Alt | 3325 | 15.3 |
| Alp | 3287 | 15.1 |
| Ast | 3247 | 14.9 |
| Bilirubin Tota | 3362 | 15.5 |
| Sbp | 60 | 0.2 |
| Dbp | 60 | 0.2 |
| Mbp | 57 | 0.2 |
| Resp Rate | 42 | 0.1 |
| Temperature | 608 | 2.7 |
| Weight | 567 | 2.6 |
| Gcs | 95 | 0.4 |
| Gcs Motor | 412 | 1.8 |
| Gcs Verbal | 243 | 1.1 |
| Gcs Eyes | 149 | 0.6 |
| Gcs Unable | 95 | 0.4 |
| Sofa | 2 | <0.1 |
| Urineoutput | 1113 | 5.1 |
| Respiration | 3912 | 18 |
| Coagulation | 227 | 1 |
| Liver | 3362 | 15.2 |
| Cardiovascular | 56 | 0.2 |
| Cns, | 120 | 0.5 |
| Renal | 42 | 0.1 |

Abbreviations:Po2,Partial pressure of oxygen;Pco2,Partial pressure of carbon dioxide;PH.Acidity;BE,Base Excess;Total_co2,Total Carbon Dioxide;SPo2,Peripheral Capillary Oxygen Saturation;Platelets,Platelet count;Wbc,White blood cell count;Bun,Blood Urea Nitrogen;Inr.International Normalized Ratio;Pt,Prothrombin Time;Ppt,Partial Thromboplastin Time;Alt,Alanine Aminotransferase;Alp,Alkaline Phosphatase;Ast,Aspartate Aminotransferase;Sbp,Systolic Blood Pressure;Dbp,Diastolic Blood Pressure;Mbp,Mean Blood Pressure;Resp Rate,Respiratory Rate;Gcs,Glasgow Coma Scale;Gcs Motor,GCS Motor Response;Gcs Verbal,GCS Verbal Response;Gcs Eyes,GCS Eye Opening;Gcs Unable,GCS Unable to Score;Sofa,Sequential Organ Failure Assessment

### Table S2. Multicollinearity test of all variables.

| Variable | VIF | Tolerance |
| --- | --- | --- |
| Age | 2.55 | 0.392 |
| ABG |  |  |
| Po2 | 1.32 | 0.759 |
| Pco2 | 7.61 | 0.131 |
| Ph | 8.56 | 0.117 |
| Baseexcess | 17.34 | 0.058 |
| Totalco2 | 15.30 | 0.065 |
| Lactate | 1.90 | 0.527 |
| Spo2 | 1.34 | 0.744 |
| Charlson |  |  |
| Myocardial_infarct | 1.21 | 0.826 |
| Congestive_heart_failure | 1.45 | 0.690 |
| Peripheral_vascular_disease | 1.08 | 0.926 |
| Cerebrovascular_disease | 1.24 | 0.809 |
| Dementia | 1.14 | 0.881 |
| Chronic_pulmonary_disease | 1.16 | 0.862 |
| Rheumatic_disease | 1.01 | 0.987 |
| Mild_liver_disease | 1.63 | 0.614 |
| Diabetes_without_cc | 1.16 | 0.860 |
| Diabetes_with_cc | 1.39 | 0.719 |
| Paraplegia | 1.17 | 0.853 |
| Renal_disease | 1.74 | 0.575 |
| Malignant_cancer | 1.45 | 0.689 |
| Severe_liver_disease | 1.85 | 0.540 |
| Metastatic_solid_tumor | 1.45 | 0.688 |
| Aids | 1.07 | 0.932 |
| Lab |  |  |
| Hematocrit | 17.21 | 0.058 |
| Hemoglobin | 16.83 | 0.059 |
| Platelets | 1.86 | 0.539 |
| Wbc | 4.01 | 0.250 |
| Aniongap | 2.67 | 0.375 |
| Bicarbonate | 4.19 | 0.239 |
| Bun | 2.24 | 0.447 |
| Calcium | 1.18 | 0.847 |
| Chloride | 5.31 | 0.188 |
| Creatinine | 2.57 | 0.390 |
| Glucose | 1.32 | 0.759 |
| Sodium | 4.14 | 0.242 |
| Potassium | 1.32 | 0.756 |
| Abs_basophils | 1.23 | 0.814 |
| Abs_eosinophils | 1.24 | 0.809 |
| Abs_lymphocytes | 1.95 | 0.512 |
| Abs_monocytes | 1.23 | 0.811 |
| Abs_neutrophils | 2.61 | 0.384 |
| Inr | 6.59 | 0.152 |
| Pt | 6.70 | 0.149 |
| Ppt | 1.17 | 0.857 |
| Alt | 3.10 | 0.323 |
| Alp | 1.16 | 0.859 |
| Ast | 3.25 | 0.308 |
| Bilirubin_total | 2.53 | 0.395 |
| Score |  |  |
| Sofa | 24.22 | 0.041 |
| Respiration | 3.06 | 0.327 |
| Coagulation | 3.54 | 0.283 |
| Liver | 3.95 | 0.253 |
| Cardiovascular | 4.92 | 0.203 |
| Cns | 4.32 | 0.232 |
| Renal | 5.63 | 0.178 |
| Urineoutput | 1.52 | 0.659 |
| Gcs | 8.19 | 0.122 |
| Gcs_motor | 3.01 | 0.332 |
| Gcs_verbal | 40.59 | 0.025 |
| Gcs_eyes | 4.04 | 0.247 |
| Gcs_unable | 50.98 | 0.020 |
| Oasis | 13.98 | 0.072 |
| Oasis_prob | 12.09 | 0.083 |
| Sapsii | 29.73 | 0.034 |
| Sapsii_prob | 24.60 | 0.041 |
| Apsiii | 5.50 | 0.182 |
| Vital Signs |  |  |
| Sbp | 4.24 | 0.236 |
| Dbp | 11.20 | 0.089 |
| Mbp | 17.06 | 0.059 |
| Resp_rate | 1.40 | 0.713 |
| Temperature | 1.21 | 0.825 |
| Weight | 1.20 | 0.834 |
| Intervention/Status |  |  |
| Nsaid | 1.26 | 0.796 |
| Preiculos | 1.12 | 0.892 |
| Mechvent | 5.25 | 0.191 |
| Electivesurgery | 1.09 | 0.918 |
| O2_flow | 1.08 | 0.922 |
| Ventilation_status | 3.83 | 0.261 |

Abbreviations:Po2,Partial pressure of oxygen;Pco2,Partial pressure of carbon dioxide;PH.Acidity;BE,Base Excess;Total_co2,Total Carbon Dioxide;SPo2,Peripheral Capillary Oxygen Saturation;Platelets,Platelet count;Wbc,White blood cell count;Bun,Blood Urea Nitrogen;Inr.International Normalized Ratio;Pt,Prothrombin Time;Ppt,Partial Thromboplastin Time;Alt,Alanine Aminotransferase;Alp,Alkaline Phosphatase;Ast,Aspartate Aminotransferase;Sbp,Systolic Blood Pressure;Dbp,Diastolic Blood Pressure;Mbp,Mean Blood Pressure;Resp Rate,Respiratory Rate;Gcs,Glasgow Coma Scale;Gcs Motor,GCS Motor Response;Gcs Verbal,GCS Verbal Response;Gcs Eyes,GCS Eye Opening;Gcs Unable,GCS Unable to Score;Sofa,Sequential Organ Failure Assessment

### Table S3. Multivariate stepwise regression analysis of variables.（SR-FS）

| Variable | OR | 95% CI | P value |
| --- | --- | --- | --- |
| age | 1.0598336 | [1.06, 1.06] | <0.001 |
| congestive_heart_failure | 2.1926617 | [2.04, 2.36] | <0.001 |
| inr | 1.2440195 | [1.16, 1.34] | <0.001 |
| weight | 1.0093609 | [1.01, 1.01] | <0.001 |
| sbp | 0.9820663 | [0.98, 0.99] | <0.001 |
| mbp | 1.0123363 | [1, 1.02] | 0.044 |
| electivesurgery | 3.9758644 | [2.85, 5.54] | <0.001 |
| ph | 2.3831271 | [1.66, 3.42] | <0.001 |
| renal_disease | 1.3001527 | [1.19, 1.42] | <0.001 |
| oasis | 1.0375818 | [1.02, 1.05] | <0.001 |
| nsaid | 1.1859408 | [1.11, 1.27] | <0.001 |
| hemoglobin | 0.8673154 | [0.82, 0.92] | <0.001 |
| cerebrovascular_disease | 1.2944544 | [1.18, 1.43] | <0.001 |
| glucose | 0.9992805 | [1, 1] | <0.001 |
| preiculos | 1.0000057 | [1, 1] | <0.001 |
| severe_liver_disease | 0.6741877 | [0.56, 0.81] | <0.001 |
| spo2 | 1.0364593 | [1.02, 1.05] | <0.001 |
| mechvent | 0.7512555 | [0.65, 0.86] | <0.001 |
| gcs | 1.0327311 | [1.01, 1.05] | 0.002 |
| po2 | 1.0009092 | [1, 1] | <0.001 |
| bicarbonate | 1.0072663 | [1, 1.02] | 0.073 |
| bun | 1.0030545 | [1, 1] | <0.001 |
| calcium | 0.9379675 | [0.9, 0.98] | 0.001 |
| aids | 0.3236976 | [0.18, 0.58] | <0.001 |
| ast | 0.9999407 | [1, 1] | 0.001 |
| apsiii | 1.0074468 | [1, 1.01] | <0.001 |
| cns | 0.8867114 | [0.85, 0.93] | <0.001 |
| renal | 0.9234689 | [0.89, 0.96] | <0.001 |
| pt | 1.0114168 | [1, 1.02] | <0.001 |
| diabetes_with_cc | 0.8373021 | [0.76, 0.93] | <0.001 |
| malignant_cancer | 0.8795467 | [0.79, 0.98] | 0.016 |
| platelets | 0.9995099 | [1, 1] | 0.001 |
| hematocrit | 1.0391851 | [1.02, 1.06] | <0.001 |
| abs_neutrophils | 1.0072749 | [1, 1.01] | 0.003 |
| aniongap | 0.9837266 | [0.97, 0.99] | <0.001 |
| ppt | 1.0014014 | [1, 1] | 0.006 |
| dbp | 1.0140993 | [1, 1.02] | 0.004 |
| abs_eosinophils | 0.8911876 | [0.79, 1] | 0.053 |
| creatinine | 1.0318885 | [1, 1.06] | 0.022 |
| dementia | 0.8589273 | [0.75, 0.98] | 0.026 |
| oasis_prob | 0.4782583 | [0.25, 0.93] | 0.029 |
| urineoutput | 1.0000368 | [1, 1] | 0.028 |
| abs_lymphocytes | 0.9936667 | [0.99, 1] | 0.068 |
| gcs_eyes | 0.9610541 | [0.93, 0.99] | 0.02 |
| temperature | 1.0457305 | [0.99, 1.11] | 0.121 |
| chloride | 0.9887793 | [0.98, 0.99] | <0.001 |
| peripheral_vascular_disease | 1.1023012 | [1, 1.22] | 0.055 |
| mild_liver_disease | 1.1183718 | [0.99, 1.26] | 0.071 |
| bilirubin_total | 0.9783669 | [0.96, 1] | 0.011 |
| lactate | 0.9803413 | [0.96, 1] | 0.041 |
| paraplegia | 1.1431664 | [0.99, 1.32] | 0.074 |
| liver | 1.0584691 | [0.99, 1.13] | 0.091 |
| metastatic_solid_tumor | 0.8552389 | [0.74, 0.99] | 0.037 |
| o2_flow | 1.0031705 | [1, 1.01] | 0.057 |
| ventilation_status | 0.9609670 | [0.92, 1] | 0.053 |
| chronic_pulmonary_disease | 0.9452592 | [0.88, 1.02] | 0.127 |
| sapsii | 1.0139683 | [1, 1.03] | 0.033 |
| potassium | 0.9681432 | [0.93, 1.01] | 0.105 |
| sapsii_prob | 0.5639032 | [0.28, 1.14] | 0.112 |

Abbreviations:Inr,International Normalized;Sbp,Systolic Blood Pressure;Mbp,Mean Blood Pressure;PH.Acidity;SPo2,Peripheral Capillary Oxygen Saturation;Gcs,Glasgow Coma Scale;Po2,Partial pressure of oxygen;Bun,Blood Urea Nitrogen;Ast,Aspartate Aminotransferase;Apsiii, Acute Physiology and Chronic Health Evaluation**;**Pt,Prothrombin Time;Ppt,Partial Thromboplastin Time;Dbp,Diastolic Blood Pressure;GCS Eye Opening;Sapsii,Simplified Acute Physiology Score II

**Table S4. Multivariate stepwise regression analysis of variables.（SR-BS）**

| Variable | OR | 95% CI | P value |
| --- | --- | --- | --- |
| apsiii | 1.0075546 | [1, 1.01] | <0.001 |
| po2 | 1.0009186 | [1, 1] | <0.001 |
| ph | 2.3912236 | [1.67, 3.43] | <0.001 |
| lactate | 0.9804288 | [0.96, 1] | 0.042 |
| congestive_heart_failure | 2.1918747 | [2.03, 2.36] | <0.001 |
| peripheral_vascular_disease | 1.1011779 | [1, 1.22] | 0.058 |
| cerebrovascular_disease | 1.2920019 | [1.17, 1.42] | <0.001 |
| dementia | 0.8551195 | [0.75, 0.98] | 0.023 |
| chronic_pulmonary_disease | 0.9449874 | [0.88, 1.02] | 0.125 |
| mild_liver_disease | 1.1176718 | [0.99, 1.26] | 0.073 |
| diabetes_with_cc | 0.8370896 | [0.76, 0.93] | <0.001 |
| paraplegia | 1.1422376 | [0.99, 1.32] | 0.076 |
| renal_disease | 1.2989031 | [1.19, 1.42] | <0.001 |
| malignant_cancer | 0.8782368 | [0.79, 0.97] | 0.015 |
| severe_liver_disease | 0.6720951 | [0.56, 0.81] | <0.001 |
| metastatic_solid_tumor | 0.8528705 | [0.74, 0.99] | 0.034 |
| aids | 0.3220347 | [0.18, 0.58] | <0.001 |
| hematocrit | 1.0397186 | [1.02, 1.06] | <0.001 |
| hemoglobin | 0.8658370 | [0.82, 0.92] | <0.001 |
| platelets | 0.9995084 | [1, 1] | 0.001 |
| aniongap | 0.9835100 | [0.97, 0.99] | <0.001 |
| bicarbonate | 1.0071747 | [1, 1.02] | 0.077 |
| bun | 1.0030461 | [1, 1] | <0.001 |
| calcium | 0.9372666 | [0.9, 0.98] | 0.001 |
| chloride | 0.9887607 | [0.98, 0.99] | <0.001 |
| creatinine | 1.0315666 | [1, 1.06] | 0.023 |
| glucose | 0.9992777 | [1, 1] | <0.001 |
| potassium | 0.9679567 | [0.93, 1.01] | 0.103 |
| abs_eosinophils | 0.8913797 | [0.79, 1] | 0.053 |
| abs_lymphocytes | 0.9936465 | [0.99, 1] | 0.066 |
| abs_neutrophils | 1.0072681 | [1, 1.01] | 0.003 |
| inr | 1.2433301 | [1.16, 1.34] | <0.001 |
| pt | 1.0113606 | [1, 1.02] | <0.001 |
| ppt | 1.0013980 | [1, 1] | 0.007 |
| ast | 0.9999407 | [1, 1] | 0.001 |
| bilirubin_total | 0.9782572 | [0.96, 0.99] | 0.011 |
| liver | 1.0576540 | [0.99, 1.13] | 0.096 |
| cns | 0.8822283 | [0.84, 0.92] | <0.001 |
| renal | 0.9230944 | [0.89, 0.96] | <0.001 |
| urineoutput | 1.0000364 | [1, 1] | 0.03 |
| sbp | 0.9820957 | [0.98, 0.99] | <0.001 |
| dbp | 1.0141137 | [1, 1.02] | 0.004 |
| mbp | 1.0121892 | [1, 1.02] | 0.046 |
| temperature | 1.0461878 | [0.99, 1.11] | 0.118 |
| spo2 | 1.0369359 | [1.02, 1.05] | <0.001 |
| weight | 1.0093647 | [1.01, 1.01] | <0.001 |
| gcs | 1.0748397 | [1.03, 1.12] | 0.002 |
| gcs_verbal | 0.9278308 | [0.85, 1.01] | 0.092 |
| gcs_eyes | 0.9382349 | [0.9, 0.98] | 0.005 |
| gcs_unable | 0.6284628 | [0.4, 1] | 0.049 |
| nsaid | 1.1850413 | [1.1, 1.27] | <0.001 |
| oasis | 1.0368325 | [1.02, 1.05] | <0.001 |
| oasis_prob | 0.4887732 | [0.25, 0.95] | 0.035 |
| age | 1.0597339 | [1.06, 1.06] | <0.001 |
| preiculos | 1.0000056 | [1, 1] | <0.001 |
| mechvent | 0.7582168 | [0.66, 0.87] | <0.001 |
| electivesurgery | 3.9623453 | [2.84, 5.53] | <0.001 |
| o2_flow | 1.0030766 | [1, 1.01] | 0.065 |
| sapsii | 1.0143697 | [1, 1.03] | 0.028 |
| sapsii_prob | 0.5553714 | [0.27, 1.13] | 0.103 |
| ventilation_status | 0.9679127 | [0.93, 1.01] | 0.128 |

Po2,Partial pressure of oxygen；PH.Acidity；Bun,Blood Urea Nitrogen;Inr.International Normalized Ratio;Pt,Prothrombin Time;Ppt,Partial Thromboplastin Time;Ast,Aspartate Aminotransferase;Sbp,Systolic Blood Pressure;Dbp,Diastolic Blood Pressure;Mbp,Mean Blood Pressure;SPo2,Peripheral Capillary Oxygen Saturation;Gcs,Glasgow Coma Scale;GCS Verbal Response;Gcs Eyes,GCS Eye Opening;Gcs Unable,GCS Unable to Score;Sapsii,Simplified Acute Physiology Score II

**Table S5. Multivariate stepwise regression analysis of variables.（SR-BE）**

| **Variable** | **OR** | **95% CI** | **P value** |
| --- | --- | --- | --- |
| age | 1.0598336 | [1.06, 1.06] | <0.001 |
| congestive_heart_failure | 2.1926617 | [2.04, 2.36] | <0.001 |
| inr | 1.2440195 | [1.16, 1.34] | <0.001 |
| weight | 1.0093609 | [1.01, 1.01] | <0.001 |
| sbp | 0.9820663 | [0.98, 0.99] | <0.001 |
| mbp | 1.0123363 | [1, 1.02] | 0.044 |
| electivesurgery | 3.9758644 | [2.85, 5.54] | <0.001 |
| ph | 2.3831271 | [1.66, 3.42] | <0.001 |
| renal_disease | 1.3001527 | [1.19, 1.42] | <0.001 |
| oasis | 1.0375818 | [1.02, 1.05] | <0.001 |
| nsaid | 1.1859408 | [1.11, 1.27] | <0.001 |
| hemoglobin | 0.8673154 | [0.82, 0.92] | <0.001 |
| cerebrovascular_disease | 1.2944544 | [1.18, 1.43] | <0.001 |
| glucose | 0.9992805 | [1, 1] | <0.001 |
| preiculos | 1.0000057 | [1, 1] | <0.001 |
| severe_liver_disease | 0.6741877 | [0.56, 0.81] | <0.001 |
| spo2 | 1.0364593 | [1.02, 1.05] | <0.001 |
| mechvent | 0.7512555 | [0.65, 0.86] | <0.001 |
| gcs | 1.0327311 | [1.01, 1.05] | 0.002 |
| po2 | 1.0009092 | [1, 1] | <0.001 |
| bicarbonate | 1.0072663 | [1, 1.02] | 0.073 |
| bun | 1.0030545 | [1, 1] | <0.001 |
| calcium | 0.9379675 | [0.9, 0.98] | 0.001 |
| aids | 0.3236976 | [0.18, 0.58] | <0.001 |
| ast | 0.9999407 | [1, 1] | 0.001 |
| apsiii | 1.0074468 | [1, 1.01] | <0.001 |
| cns | 0.8867114 | [0.85, 0.93] | <0.001 |
| renal | 0.9234689 | [0.89, 0.96] | <0.001 |
| pt | 1.0114168 | [1, 1.02] | <0.001 |
| diabetes_with_cc | 0.8373021 | [0.76, 0.93] | <0.001 |
| malignant_cancer | 0.8795467 | [0.79, 0.98] | 0.016 |
| platelets | 0.9995099 | [1, 1] | 0.001 |
| hematocrit | 1.0391851 | [1.02, 1.06] | <0.001 |
| abs_neutrophils | 1.0072749 | [1, 1.01] | 0.003 |
| aniongap | 0.9837266 | [0.97, 0.99] | <0.001 |
| ppt | 1.0014014 | [1, 1] | 0.006 |
| dbp | 1.0140993 | [1, 1.02] | 0.004 |
| abs_eosinophils | 0.8911876 | [0.79, 1] | 0.053 |
| creatinine | 1.0318885 | [1, 1.06] | 0.022 |
| dementia | 0.8589273 | [0.75, 0.98] | 0.026 |
| oasis_prob | 0.4782583 | [0.25, 0.93] | 0.029 |
| urineoutput | 1.0000368 | [1, 1] | 0.028 |
| abs_lymphocytes | 0.9936667 | [0.99, 1] | 0.068 |
| gcs_eyes | 0.9610541 | [0.93, 0.99] | 0.02 |
| temperature | 1.0457305 | [0.99, 1.11] | 0.121 |
| chloride | 0.9887793 | [0.98, 0.99] | <0.001 |
| peripheral_vascular_disease | 1.1023012 | [1, 1.22] | 0.055 |
| mild_liver_disease | 1.1183718 | [0.99, 1.26] | 0.071 |
| bilirubin_total | 0.9783669 | [0.96, 1] | 0.011 |
| lactate | 0.9803413 | [0.96, 1] | 0.041 |
| paraplegia | 1.1431664 | [0.99, 1.32] | 0.074 |
| liver | 1.0584691 | [0.99, 1.13] | 0.091 |
| metastatic_solid_tumor | 0.8552389 | [0.74, 0.99] | 0.037 |
| o2_flow | 1.0031705 | [1, 1.01] | 0.057 |
| ventilation_status | 0.9609670 | [0.92, 1] | 0.053 |
| chronic_pulmonary_disease | 0.9452592 | [0.88, 1.02] | 0.127 |
| sapsii | 1.0139683 | [1, 1.03] | 0.033 |
| potassium | 0.9681432 | [0.93, 1.01] | 0.105 |
| sapsii_prob | 0.5639032 | [0.28, 1.14] | 0.112 |

Abbreviations:Inr.International Normalized Ratio;Sbp,Systolic Blood Pressure;Dbp,Diastolic Blood Pressure;PH.Acidity;SPo2,Peripheral Capillary Oxygen Saturation;Bun,Blood Urea Nitrogen;Ast,Aspartate Aminotransferase;Apsiii, Acute Physiology and Chronic Health Evaluation;Pt,Prothrombin Time;Ppt,Partial Thromboplastin Time;Dbp,Diastolic Blood Pressure;oasis,Open Source Anonymized Simulator;GCS Eye Opening;Gcs Unable;Sapsii,Simplified Acute Physiology Score II；

### Table S6. Baseline characteristics of the training and validation sets.

| **Variable** |  | **Train** | **Test** | **p** |
| --- | --- | --- | --- | --- |
| n |  | 17275(80%) | 4319(20%) |  |
| age (median [IQR]) |  | 68.77 [57.51, 79.15] | 68.27 [56.83, 79.13] | 0.274 |
| ABG |  |  |  |  |
| po2 (median [IQR]) |  | 78.00 [49.00, 139.00] | 80.00 [50.00, 146.00] | 0.034 |
| pco2 (median [IQR]) |  | 43.00 [36.00, 53.00] | 43.00 [36.00, 52.00] | 0.153 |
| ph (median [IQR]) |  | 7.36 [7.29, 7.42] | 7.36 [7.29, 7.42] | 0.738 |
| baseexcess (median [IQR]) |  | 0.00 [-4.00, 2.00] | 0.00 [-4.00, 2.00] | 0.233 |
| totalco2 (median [IQR]) |  | 25.00 [22.00, 29.00] | 25.00 [21.00, 29.00] | 0.128 |
| lactate (median [IQR]) |  | 1.70 [1.20, 2.60] | 1.70 [1.20, 2.60] | 0.203 |
| spo2 (median [IQR]) |  | 96.71 [94.96, 98.32] | 96.83 [95.05, 98.43] | 0.025 |
| **Charlson** |  |  |  |  |
| myocardial_infarct (median [IQR]) |  | 0.00 [0.00, 0.00] | 0.00 [0.00, 0.00] | 0.390 |
| congestive_heart_failure (median [IQR]) |  | 0.00 [0.00, 1.00] | 0.00 [0.00, 1.00] | 0.577 |
| peripheral_vascular_disease (median [IQR]) |  | 0.00 [0.00, 0.00] | 0.00 [0.00, 0.00] | 0.254 |
| cerebrovascular disease (median [IQR]) |  | 0.00 [0.00, 0.00] | 0.00 [0.00, 0.00] | 0.389 |
| dementia (median [IQR]) |  | 0.00 [0.00, 0.00] | 0.00 [0.00, 0.00] | 0.615 |
| chronic_pulmonary_disease (median [IQR]) |  | 0.00 [0.00, 1.00] | 0.00 [0.00, 1.00] | 0.341 |
| rheumatic_disease (median [IQR]) |  | 0.00 [0.00, 0.00] | 0.00 [0.00, 0.00] | 0.274 |
| peptic_ulcer_disease (median [IQR]) |  | 0.00 [0.00, 0.00] | 0.00 [0.00, 0.00] | 0.313 |
| mild_liver_disease (median [IQR]) |  | 0.00 [0.00, 0.00] | 0.00 [0.00, 0.00] | 0.962 |
| diabetes_without_cc (median [IQR]) |  | 0.00 [0.00, 0.00] | 0.00 [0.00, 1.00] | 0.349 |
| diabetes_with_cc (median [IQR]) |  | 0.00 [0.00, 0.00] | 0.00 [0.00, 0.00] | 0.699 |
| paraplegia (median [IQR]) |  | 0.00 [0.00, 0.00] | 0.00 [0.00, 0.00] | 0.154 |
| renal_disease (median [IQR]) |  | 0.00 [0.00, 1.00] | 0.00 [0.00, 1.00] | 0.686 |
| malignant_cancer (median [IQR]) |  | 0.00 [0.00, 0.00] | 0.00 [0.00, 0.00] | 0.552 |
| severe_liver_disease (median [IQR]) |  | 0.00 [0.00, 0.00] | 0.00 [0.00, 0.00] | 0.850 |
| metastatic_solidtumor (median [IQR]) |  | 0.00 [0.00, 0.00] | 0.00 [0.00, 0.00] | 0.263 |
| aids (median [IQR]) |  | 0.00 [0.00, 0.00] | 0.00 [0.00, 0.00] | 0.798 |
| **Lab** |  |  |  |  |
| hematocrit (median [IQR]) |  | 34.10 [29.50, 39.40] | 34.20 [29.40, 39.40] | 0.808 |
| hemoglobin (median [IQR]) |  | 10.90 [9.30, 12.70] | 11.00 [9.30, 12.70] | 0.594 |
| platelets (median [IQR]) |  | 220.00 [156.00, 295.00] | 218.00 [156.00, 296.00] | 0.757 |
| wbc (median [IQR]) |  | 13.20 [9.40, 18.40] | 13.10 [9.30, 18.60] | 0.679 |
| aniongap (median [IQR]) |  | 16.00 [13.00, 19.00] | 16.00 [13.00, 19.00] | 0.404 |
| bicarbonate (median [IQR]) |  | 24.00 [21.00, 28.00] | 24.00 [21.00, 28.00] | 0.317 |
| bun (median [IQR]) |  | 26.00 [17.00, 44.00] | 26.00 [17.00, 43.00] | 0.459 |
| calcium (median [IQR]) |  | 8.70 [8.20, 9.10] | 8.60 [8.20, 9.20] | 0.892 |
| chloride (median [IQR]) |  | 104.00 [100.00, 109.00] | 104.00 [100.00, 109.00] | 0.504 |
| creatinine (median [IQR]) |  | 1.20 [0.80, 2.10] | 1.20 [0.80, 2.10] | 0.210 |
| glucose (median [IQR]) |  | 154.00 [123.00, 209.00] | 156.00 [124.00, 209.00] | 0.614 |
| sodium (median [IQR]) |  | 140.00 [137.00, 143.00] | 140.00 [137.00, 143.00] | 0.354 |
| potassium (median [IQR]) |  | 4.50 [4.10, 5.10] | 4.50 [4.10, 5.10] | 0.242 |
| abs_basophils (median [IQR]) |  | 0.02 [0.00, 0.05] | 0.02 [0.00, 0.04] | 0.615 |
| abs_eosinophils (median [IQR]) |  | 0.03 [0.00, 0.12] | 0.03 [0.00, 0.12] | 0.610 |
| abs_lymphocytes (median [IQR]) |  | 1.05 [0.64, 1.66] | 1.06 [0.62, 1.68] | 0.720 |
| abs_monocytes (median [IQR]) |  | 0.65 [0.38, 1.01] | 0.64 [0.39, 1.00] | 0.754 |
| abs_neutrophils (median [IQR]) |  | 9.85 [6.48, 14.37] | 9.69 [6.46, 14.23] | 0.276 |
| inr (median [IQR]) |  | 1.30 [1.20, 1.70] | 1.30 [1.20, 1.70] | 0.733 |
| pt (median [IQR]) |  | 14.60 [12.80, 18.80] | 14.70 [12.80, 18.80] | 0.804 |
| ppt (median [IQR]) |  | 33.10 [28.50, 46.00] | 32.80 [28.40, 46.30] | 0.335 |
| alt (median [IQR]) |  | 28.00 [16.00, 61.00] | 28.00 [17.00, 62.00] | 0.575 |
| alp (median [IQR]) |  | 92.00 [67.00, 134.00] | 92.00 [67.00, 134.00] | 0.826 |
| ast (median [IQR]) |  | 41.00 [24.00, 91.00] | 41.00 [24.50, 89.00] | 0.375 |
| bilirubin_total (median [IQR]) |  | 0.60 [0.40, 1.10] | 0.60 [0.40, 1.10] | 0.688 |
| **Score** |  |  |  |  |
| sofa (median [IQR]) |  | 5.00 [3.00, 8.00] | 5.00 [3.00, 9.00] | 0.756 |
| respiration (median [IQR]) |  | 2.00 [2.00, 3.00] | 2.00 [2.00, 3.00] | 0.587 |
| coagulation (median [IQR]) |  | 0.00 [0.00, 1.00] | 0.00 [0.00, 1.00] | 0.655 |
| liver (median [IQR]) |  | 0.00 [0.00, 0.00] | 0.00 [0.00, 0.00] | 0.609 |
| cardiovascular (median [IQR]) |  | 1.00 [1.00, 3.00] | 1.00 [1.00, 3.00] | 0.854 |
| cns (median [IQR]) |  | 0.00 [0.00, 1.00] | 0.00 [0.00, 1.00] | 0.926 |
| renal (median [IQR]) |  | 1.00 [0.00, 2.00] | 1.00 [0.00, 2.00] | 0.228 |
| urineoutput (median [IQR]) |  | 1302.00 [725.00, 2145.00] | 1335.00 [733.50, 2193.50] | 0.226 |
| gcs (median [IQR]) |  | 15.00 [15.00, 15.00] | 15.00 [15.00, 15.00] | 0.539 |
| gcs_motor (median [IQR]) |  | 6.00 [4.00, 6.00] | 6.00 [4.00, 6.00] | 0.038 |
| gcs_verbal (median [IQR]) |  | 2.00 [0.00, 5.00] | 1.00 [0.00, 5.00] | 0.379 |
| gcs_eyes (median [IQR]) |  | 4.00 [1.00, 4.00] | 3.00 [1.00, 4.00] | 0.107 |
| gcs_unable (median [IQR]) |  | 0.00 [0.00, 1.00] | 0.00 [0.00, 1.00] | 0.624 |
| oasis (median [IQR]) |  | 35.00 [29.00, 41.00] | 35.00 [29.00, 41.00] | 0.457 |
| oasis_prob (median [IQR]) |  | 0.15 [0.08, 0.28] | 0.15 [0.08, 0.28] | 0.457 |
| sapsii (median [IQR]) |  | 40.00 [31.00, 50.00] | 39.00 [30.00, 51.00] | 0.664 |
| sapsii_prob (median [IQR]) |  | 0.25 [0.12, 0.46] | 0.23 [0.11, 0.48] | 0.664 |
| apsiii (median [IQR]) |  | 48.00 [35.00, 64.00] | 48.00 [35.00, 65.50] | 0.392 |
| **Vital signs** |  |  |  |  |
| sbp (median [IQR]) |  | 113.74 [104.75, 125.84] | 113.92 [104.69, 125.94] | 0.954 |
| dbp (median [IQR]) |  | 62.35 [55.88, 69.98] | 62.55 [55.87, 69.93] | 0.595 |
| mbp (median [IQR]) |  | 76.79 [70.65, 84.38] | 76.70 [70.60, 84.51] | 0.900 |
| resp_rate (median [IQR]) |  | 20.26 [17.66, 23.33] | 20.22 [17.73, 23.23] | 0.914 |
| temperature (median [IQR]) |  | 36.86 [36.61, 37.18] | 36.84 [36.60, 37.17] | 0.082 |
| weight (median [IQR]) |  | 78.30 [64.90, 95.66] | 79.00 [65.00, 96.10] | 0.334 |
| **Intervention/Status** |  |  |  |  |
| nsaid (median [IQR]) |  | 0.00 [0.00, 1.00] | 0.00 [0.00, 1.00] | 0.055 |
| preiculos (median [IQR]) |  | 112.00 [48.05, 1543.98] | 110.00 [45.00, 1318.21] | 0.262 |
| mechvent (median [IQR]) |  | 1.00 [0.00, 1.00] | 1.00 [0.00, 1.00] | 0.563 |
| electivesurgery (median [IQR]) |  | 0.00 [0.00, 0.00] | 0.00 [0.00, 0.00] | 0.753 |
| o2_flow (median [IQR]) |  | 8.00 [3.00, 10.00] | 8.00 [4.00, 10.00] | 0.489 |
| ventilation_status (median [IQR]) |  | 4.00 [1.00, 4.00] | 4.00 [1.00, 4.00] | 0.365 |

Abbreviations:Po2,Partial pressure of oxygen;Pco2,Partial pressure of carbon dioxide;PH.Acidity;BE,Base Excess;Total_co2,Total Carbon Dioxide;SPo2,Peripheral Capillary Oxygen Saturation;Platelets,Platelet count;Wbc,White blood cell count;Bun,Blood Urea Nitrogen;Inr.International Normalized Ratio;Pt,Prothrombin Time;Ppt,Partial Thromboplastin Time;Alt,Alanine Aminotransferase;Alp,Alkaline Phosphatase;Ast,Aspartate Aminotransferase;Sbp,Systolic Blood Pressure;Dbp,Diastolic Blood Pressure;Mbp,Mean Blood Pressure;Resp Rate,Respiratory Rate;Gcs,Glasgow Coma Scale;Gcs Motor,GCS Motor Response;Gcs Verbal,GCS Verbal Response;Gcs Eyes,GCS Eye Opening;Gcs Unable,GCS Unable to Score;Sofa,Sequential Organ Failure Assessment;Apsiii, Acute Physiology and Chronic Health Evaluation;Sapsii,Simplified Acute Physiology Score II；oasis,Open Source Anonymized Simulator

### Table S7. Results of the multivariable logistic regressio model(Internal validation model).

| Variables | β | S.E | Z | *P* | OR (95%CI) |
| --- | --- | --- | --- | --- | --- |
|  |  |  |  |  |  |
| Intercept | -18.23 | 1.81 | -10.06 | **<.001** | 0.00 (0.00 ~ 0.00) |
| Abs Eosinophils | -0.11 | 0.06 | -1.87 | 0.062 | 0.90 (0.80 ~ 1.01) |
| Abs Lymphocytes | -0.01 | 0.00 | -1.85 | 0.064 | 0.99 (0.99 ~ 1.00) |
| Abs Neutrophils | 0.01 | 0.00 | 2.77 | **0.006** | 1.01 (1.01 ~ 1.01) |
| Aids |  |  |  |  |  |
| 0 |  |  |  |  | 1.00 (Reference) |
| 1 | -1.16 | 0.30 | -3.87 | **<.001** | 0.31 (0.17 ~ 0.56) |
| Age | 0.06 | 0.00 | 30.51 | **<.001** | 1.06 (1.05 ~ 1.06) |
| Aniongap | -0.02 | 0.00 | -3.80 | **<.001** | 0.98 (0.97 ~ 0.99) |
| Apsiii | 0.01 | 0.00 | 4.91 | **<.001** | 1.01 (1.01 ~ 1.01) |
| Ast | -0.01 | 0.00 | -3.34 | **<.001** | 0.99 (0.99 ~ 0.99) |
| Bicarbonate | 0.01 | 0.00 | 1.87 | 0.061 | 1.01 (1.00 ~ 1.02) |
| Bilirubin Total | -0.00 | 0.01 | -0.17 | 0.867 | 1.00 (0.97 ~ 1.02) |
| Bun | 0.01 | 0.00 | 2.56 | **0.010** | 1.01 (1.01 ~ 1.01) |
| Calcium | -0.06 | 0.02 | -3.24 | **0.001** | 0.94 (0.90 ~ 0.97) |
| Cerebrovascular Disease |  |  |  |  |  |
| 0 |  |  |  |  | 1.00 (Reference) |
| 1 | 0.26 | 0.05 | 5.29 | **<.001** | 1.30 (1.18 ~ 1.43) |
| Chloride | -0.01 | 0.00 | -4.00 | **<.001** | 0.99 (0.98 ~ 0.99) |
| Chronic Pulmonary Disease |  |  |  |  |  |
| 0 |  |  |  |  | 1.00 (Reference) |
| 1 | -0.06 | 0.04 | -1.56 | 0.119 | 0.94 (0.88 ~ 1.01) |
| Cns |  |  |  |  |  |
| 0 |  |  |  |  | 1.00 (Reference) |
| 1 | -0.03 | 0.05 | -0.55 | 0.580 | 0.97 (0.89 ~ 1.07) |
| 2 | -0.15 | 0.07 | -2.09 | **0.036** | 0.86 (0.75 ~ 0.99) |
| 3 | -0.41 | 0.08 | -4.90 | **<.001** | 0.66 (0.56 ~ 0.78) |
| 4 | -0.62 | 0.12 | -5.18 | **<.001** | 0.54 (0.42 ~ 0.68) |
| Congestive Heart Failure |  |  |  |  |  |
| 0 |  |  |  |  | 1.00 (Reference) |
| 1 | 0.77 | 0.04 | 20.21 | **<.001** | 2.15 (2.00 ~ 2.32) |
| Creatinine | 0.04 | 0.01 | 2.59 | **0.010** | 1.04 (1.01 ~ 1.07) |
| Dbp | 0.01 | 0.00 | 2.60 | **0.009** | 1.01 (1.01 ~ 1.02) |
| Dementia |  |  |  |  |  |
| 0 |  |  |  |  | 1.00 (Reference) |
| 1 | -0.18 | 0.07 | -2.66 | **0.008** | 0.83 (0.73 ~ 0.95) |
| Diabetes With Cc |  |  |  |  |  |
| 0 |  |  |  |  | 1.00 (Reference) |
| 1 | -0.18 | 0.05 | -3.41 | **<.001** | 0.84 (0.75 ~ 0.93) |
| Electivesurgery |  |  |  |  |  |
| 0 |  |  |  |  | 1.00 (Reference) |
| 1 | 1.45 | 0.17 | 8.64 | **<.001** | 4.27 (3.07 ~ 5.94) |
| Gcs | 0.03 | 0.01 | 2.48 | **0.013** | 1.03 (1.01 ~ 1.05) |
| Hemoglobin | -0.13 | 0.03 | -4.57 | **<.001** | 0.88 (0.83 ~ 0.93) |
| Gcs Eyes |  |  |  |  |  |
| 1 |  |  |  |  | 1.00 (Reference) |
| 2 | -0.13 | 0.07 | -1.90 | 0.057 | 0.88 (0.77 ~ 1.00) |
| 3 | -0.17 | 0.06 | -2.70 | **0.007** | 0.85 (0.75 ~ 0.96) |
| 4 | -0.18 | 0.05 | -3.35 | **<.001** | 0.84 (0.75 ~ 0.93) |
| Glucose | -0.01 | 0.00 | -4.64 | **<.001** | 0.99 (0.99 ~ 0.99) |
| Hematocrit | 0.03 | 0.01 | 3.53 | **<.001** | 1.04 (1.02 ~ 1.06) |
| Inr | 0.22 | 0.04 | 5.78 | **<.001** | 1.24 (1.15 ~ 1.33) |
| Liver |  |  |  |  |  |
| 0 |  |  |  |  | 1.00 (Reference) |
| 1 | 0.22 | 0.06 | 3.87 | **<.001** | 1.25 (1.12 ~ 1.39) |
| 2 | 0.02 | 0.08 | 0.27 | 0.785 | 1.02 (0.88 ~ 1.18) |
| 3 | -0.13 | 0.16 | -0.78 | 0.437 | 0.88 (0.64 ~ 1.21) |
| 4 | -0.30 | 0.33 | -0.91 | 0.364 | 0.74 (0.39 ~ 1.41) |
| Lactate | -0.02 | 0.01 | -1.90 | 0.058 | 0.98 (0.96 ~ 1.00) |
| Malignant Cancer |  |  |  |  |  |
| 0 |  |  |  |  | 1.00 (Reference) |
| 1 | -0.15 | 0.05 | -2.72 | **0.007** | 0.86 (0.78 ~ 0.96) |
| Mbp | 0.01 | 0.01 | 2.16 | **0.031** | 1.01 (1.01 ~ 1.03) |
| Metastatic Solid Tumor |  |  |  |  |  |
| 0 |  |  |  |  | 1.00 (Reference) |
| 1 | -0.16 | 0.08 | -2.19 | **0.028** | 0.85 (0.73 ~ 0.98) |
| Mechvent |  |  |  |  |  |
| 0 |  |  |  |  | 1.00 (Reference) |
| 1 | -0.27 | 0.08 | -3.56 | **<.001** | 0.76 (0.65 ~ 0.88) |
| Mild Liver Disease |  |  |  |  |  |
| 0 |  |  |  |  | 1.00 (Reference) |
| 1 | 0.11 | 0.06 | 1.82 | 0.068 | 1.12 (0.99 ~ 1.26) |
| Nsaid |  |  |  |  |  |
| 0 |  |  |  |  | 1.00 (Reference) |
| 1 | 0.18 | 0.04 | 4.90 | **<.001** | 1.19 (1.11 ~ 1.28) |
| Oasis | 0.03 | 0.01 | 4.26 | **<.001** | 1.03 (1.02 ~ 1.05) |
| Oasis Prob | -0.54 | 0.34 | -1.56 | 0.119 | 0.59 (0.30 ~ 1.15) |
| Peripheral Vascular Disease |  |  |  |  |  |
| 0 |  |  |  |  | 1.00 (Reference) |
| 1 | 0.10 | 0.05 | 1.98 | **0.047** | 1.11 (1.01 ~ 1.22) |
| Paraplegia |  |  |  |  |  |
| 0 |  |  |  |  | 1.00 (Reference) |
| 1 | 0.14 | 0.08 | 1.80 | 0.072 | 1.14 (0.99 ~ 1.33) |
| Ph | 0.99 | 0.18 | 5.41 | **<.001** | 2.70 (1.88 ~ 3.87) |
| Platelets | -0.01 | 0.00 | -3.05 | **0.002** | 0.99 (0.99 ~ 0.99) |
| Ppt | 0.01 | 0.00 | 2.72 | **0.007** | 1.01 (1.01 ~ 1.01) |
| Preiculos | 0.01 | 0.00 | 4.03 | **<.001** | 1.01 (1.01 ~ 1.01) |
| Pt | 0.01 | 0.00 | 3.50 | **<.001** | 1.01 (1.01 ~ 1.02) |
| Renal |  |  |  |  |  |
| 0 |  |  |  |  | 1.00 (Reference) |
| 1 | 0.01 | 0.05 | 0.15 | 0.882 | 1.01 (0.92 ~ 1.11) |
| 2 | -0.12 | 0.07 | -1.71 | 0.087 | 0.89 (0.78 ~ 1.02) |
| 3 | -0.23 | 0.07 | -3.28 | **0.001** | 0.79 (0.69 ~ 0.91) |
| 4 | -0.37 | 0.09 | -3.99 | **<.001** | 0.69 (0.57 ~ 0.83) |
| Renal Disease |  |  |  |  |  |
| 0 |  |  |  |  | 1.00 (Reference) |
| 1 | 0.24 | 0.05 | 5.15 | **<.001** | 1.27 (1.16 ~ 1.40) |
| Sapsii | 0.02 | 0.01 | 2.38 | **0.017** | 1.02 (1.01 ~ 1.03) |
| Sapsii Prob | -0.59 | 0.36 | -1.61 | 0.107 | 0.56 (0.27 ~ 1.13) |
| Sbp | -0.02 | 0.00 | -8.66 | **<.001** | 0.98 (0.98 ~ 0.99) |
| Severe Liver Disease |  |  |  |  |  |
| 0 |  |  |  |  | 1.00 (Reference) |
| 1 | -0.40 | 0.09 | -4.19 | **<.001** | 0.67 (0.56 ~ 0.81) |
| Spo2 | 0.04 | 0.01 | 5.93 | **<.001** | 1.04 (1.03 ~ 1.05) |
| Temperature | 0.04 | 0.03 | 1.24 | 0.214 | 1.04 (0.98 ~ 1.10) |
| Urineoutput | 0.00 | 0.00 | 1.78 | 0.074 | 1.00 (1.00 ~ 1.00) |
| Weight | 0.01 | 0.00 | 13.27 | **<.001** | 1.01 (1.01 ~ 1.01) |
| Ventilation Status |  |  |  |  |  |
| 0 |  |  |  |  | 1.00 (Reference) |
| 1 | 0.06 | 0.08 | 0.74 | 0.459 | 1.06 (0.90 ~ 1.25) |
| 2 | 0.11 | 0.12 | 0.91 | 0.362 | 1.11 (0.88 ~ 1.40) |
| 3 | -0.08 | 0.12 | -0.66 | 0.508 | 0.92 (0.72 ~ 1.18) |
| 4 | -0.03 | 0.11 | -0.27 | 0.791 | 0.97 (0.79 ~ 1.20) |

Abbreviations:Apsiii, Acute Physiology and Chronic Health Evaluation;Ast,Aspartate Aminotransferase;Bun,Blood Urea Nitrogen;Dbp,Diastolic Blood Pressure;Gcs,Glasgow Coma Scale;GCS Eye Opening;Inr.International Normalized Ratio;Mbp,Mean Blood Pressure;oasis,Open Source Anonymized Simulator;PH.Acidity;Pt,Prothrombin Time;Ppt,Partial Thromboplastin Time;Sapsii,Simplified Acute Physiology Score II；Sbp,Systolic Blood Pressure;SPo2,Peripheral Capillary Oxygen Saturation;

### Table S8. Results of the multivariable logistic regression model (External validation model).

| Variables | β | S.E | Z | *P* | OR (95%CI) |
| --- | --- | --- | --- | --- | --- |
|  |  |  |  |  |  |
| Respiration |  |  |  |  |  |
| 0 |  |  |  |  | 1.00 (Reference) |
| 1 | -0.08 | 0.14 | -0.58 | 0.562 | 0.92 (0.69 ~ 1.22) |
| 2 | 0.02 | 0.07 | 0.27 | 0.788 | 1.02 (0.88 ~ 1.18) |
| 3 | -0.06 | 0.07 | -0.88 | 0.379 | 0.94 (0.81 ~ 1.08) |
| 4 | -0.48 | 0.10 | -4.72 | **<.001** | 0.62 (0.51 ~ 0.76) |
| Coagulation |  |  |  |  |  |
| 0 |  |  |  |  | 1.00 (Reference) |
| 1 | 0.31 | 0.08 | 4.04 | **<.001** | 1.36 (1.17 ~ 1.58) |
| 2 | -0.25 | 0.10 | -2.57 | **0.010** | 0.78 (0.64 ~ 0.94) |
| 3 | -0.52 | 0.15 | -3.48 | **<.001** | 0.59 (0.44 ~ 0.80) |
| 4 | -0.70 | 0.28 | -2.53 | **0.011** | 0.50 (0.29 ~ 0.85) |
| Cardiovascular |  |  |  |  |  |
| 0 |  |  |  |  | 1.00 (Reference) |
| 1 | 0.55 | 0.09 | 5.99 | **<.001** | 1.74 (1.45 ~ 2.09) |
| 2 | 0.58 | 0.23 | 2.50 | **0.012** | 1.79 (1.13 ~ 2.82) |
| 3 | 0.78 | 0.13 | 6.12 | **<.001** | 2.18 (1.70 ~ 2.79) |
| 4 | 0.55 | 0.11 | 5.19 | **<.001** | 1.74 (1.41 ~ 2.15) |
| Liver |  |  |  |  |  |
| 0 |  |  |  |  | 1.00 (Reference) |
| 1 | 0.33 | 0.10 | 3.38 | **<.001** | 1.38 (1.15 ~ 1.67) |
| 2 | -0.33 | 0.10 | -3.30 | **<.001** | 0.72 (0.59 ~ 0.87) |
| 3 | -0.51 | 0.19 | -2.69 | **0.007** | 0.60 (0.41 ~ 0.87) |
| 4 | -0.63 | 0.20 | -3.23 | **0.001** | 0.53 (0.36 ~ 0.78) |
| Cns |  |  |  |  |  |
| 0 |  |  |  |  | 1.00 (Reference) |
| 1 | 0.16 | 0.07 | 2.11 | **0.035** | 1.17 (1.01 ~ 1.35) |
| 2 | 0.23 | 0.11 | 2.18 | **0.030** | 1.26 (1.02 ~ 1.56) |
| 3 | 0.12 | 0.10 | 1.19 | 0.233 | 1.13 (0.92 ~ 1.38) |
| 4 | -0.23 | 0.14 | -1.61 | 0.108 | 0.79 (0.60 ~ 1.05) |
| Gcs Eyes |  |  |  |  |  |
| 1 |  |  |  |  | 1.00 (Reference) |
| 2 | 0.05 | 0.10 | 0.45 | 0.653 | 1.05 (0.85 ~ 1.28) |
| 3 | 0.11 | 0.08 | 1.34 | 0.181 | 1.11 (0.95 ~ 1.30) |
| 4 | 0.18 | 0.07 | 2.46 | **0.014** | 1.20 (1.04 ~ 1.38) |
| Renal |  |  |  |  |  |
| 0 |  |  |  |  | 1.00 (Reference) |
| 1 | 0.47 | 0.07 | 6.60 | **<.001** | 1.60 (1.39 ~ 1.84) |
| 2 | 0.59 | 0.09 | 6.59 | **<.001** | 1.80 (1.51 ~ 2.14) |
| 3 | 0.37 | 0.10 | 3.72 | **<.001** | 1.45 (1.19 ~ 1.76) |
| 4 | 0.37 | 0.10 | 3.67 | **<.001** | 1.44 (1.19 ~ 1.76) |
| Electivesurgery |  |  |  |  |  |
| 0 |  |  |  |  | 1.00 (Reference) |
| 1 | 0.38 | 0.14 | 2.66 | **0.008** | 1.46 (1.10 ~ 1.93) |
| Mechvent |  |  |  |  |  |
| 0 |  |  |  |  | 1.00 (Reference) |
| 1 | -0.30 | 0.06 | -4.78 | **<.001** | 0.74 (0.65 ~ 0.84) |
| Age | 0.01 | 0.00 | 14.38 | **<.001** | 1.01 (1.01 ~ 1.01) |
| Apsiii | 0.01 | 0.00 | 3.28 | **0.001** | 1.01 (1.01 ~ 1.01) |
| Abs Eosinophils | -0.02 | 0.03 | -0.52 | 0.601 | 0.98 (0.93 ~ 1.04) |
| Po2 | -0.00 | 0.00 | -0.23 | 0.817 | 1.00 (1.00 ~ 1.00) |
| Abs Lymphocytes | -0.01 | 0.01 | -1.05 | 0.292 | 0.99 (0.98 ~ 1.01) |
| Apsiii Prob | 0.11 | 0.16 | 0.69 | 0.491 | 1.11 (0.82 ~ 1.52) |
| Sofa | -0.01 | 0.01 | -0.68 | 0.498 | 0.99 (0.98 ~ 1.01) |
| Abs Neutrophils | -0.05 | 0.21 | -0.22 | 0.823 | 0.95 (0.63 ~ 1.44) |
| Heartrate | -0.01 | 0.00 | -5.80 | **<.001** | 0.99 (0.99 ~ 0.99) |
| Sbp | -0.01 | 0.00 | -3.39 | **<.001** | 0.99 (0.99 ~ 0.99) |
| Resprate Mean | -0.00 | 0.01 | -0.07 | 0.944 | 1.00 (0.99 ~ 1.01) |
| Temperature | -0.27 | 0.04 | -7.34 | **<.001** | 0.76 (0.71 ~ 0.82) |
| Mbp | -0.02 | 0.00 | -8.60 | **<.001** | 0.98 (0.97 ~ 0.98) |
| Aniongap | -0.02 | 0.01 | -2.83 | **0.005** | 0.98 (0.97 ~ 0.99) |
| Albumin | -0.08 | 0.04 | -1.94 | 0.053 | 0.92 (0.85 ~ 1.00) |
| Spo2 | 0.01 | 0.01 | 0.72 | 0.473 | 1.01 (0.99 ~ 1.02) |
| Bicarbonate | 0.03 | 0.00 | 6.54 | **<.001** | 1.03 (1.02 ~ 1.04) |
| Bilirubin Total | -0.04 | 0.01 | -4.32 | **<.001** | 0.96 (0.95 ~ 0.98) |
| Creatinine | 0.05 | 0.02 | 3.39 | **<.001** | 1.05 (1.02 ~ 1.08) |
| Chloride | -0.01 | 0.00 | -3.12 | **0.002** | 0.99 (0.98 ~ 0.99) |
| Glucose | -0.01 | 0.00 | -2.04 | **0.042** | 0.99 (0.99 ~ 0.99) |
| Hematocrit | -0.01 | 0.00 | -3.08 | **0.002** | 0.99 (0.98 ~ 0.99) |
| Lactate | -0.04 | 0.01 | -3.49 | **<.001** | 0.96 (0.94 ~ 0.98) |
| Platelets | 0.00 | 0.00 | 0.54 | 0.589 | 1.00 (1.00 ~ 1.00) |
| Potassium | 0.10 | 0.03 | 3.45 | **<.001** | 1.10 (1.04 ~ 1.16) |
| Ppt | 0.01 | 0.00 | 4.21 | **<.001** | 1.01 (1.01 ~ 1.01) |
| Inr | 0.23 | 0.02 | 10.56 | **<.001** | 1.26 (1.21 ~ 1.32) |
| Pt | 0.03 | 0.00 | 11.41 | **<.001** | 1.03 (1.03 ~ 1.04) |
| Sodium | 0.01 | 0.01 | 1.08 | 0.281 | 1.01 (1.00 ~ 1.02) |
| Wbc | -0.00 | 0.00 | -0.22 | 0.823 | 1.00 (1.00 ~ 1.00) |
| Gcs | 0.00 | 0.01 | 0.00 | 0.999 | 1.00 (0.98 ~ 1.02) |
| Gcsmotor | 0.04 | 0.02 | 2.46 | **0.014** | 1.04 (1.01 ~ 1.08) |
| Gcsverbal | 0.04 | 0.01 | 3.10 | **0.002** | 1.04 (1.01 ~ 1.07) |
| Sapsii | 0.01 | 0.00 | 8.52 | **<.001** | 1.01 (1.01 ~ 1.02) |
| Sapsii Prob | 0.79 | 0.11 | 7.42 | **<.001** | 2.20 (1.79 ~ 2.71) |
| Bun | 0.01 | 0.00 | 9.64 | **<.001** | 1.01 (1.01 ~ 1.01) |
| Hemoglobin | -0.06 | 0.01 | -4.89 | **<.001** | 0.94 (0.91 ~ 0.96) |
| Oasis | 0.02 | 0.00 | 4.99 | **<.001** | 1.02 (1.01 ~ 1.02) |
| Oasis Prob | 0.72 | 0.15 | 4.85 | **<.001** | 2.05 (1.54 ~ 2.75) |
| Preiculos | 0.00 | 0.00 | 0.17 | 0.861 | 1.00 (1.00 ~ 1.00) |
| Baseexcess | 0.03 | 0.00 | 7.20 | **<.001** | 1.03 (1.02 ~ 1.04) |
| Urineoutput | -0.01 | 0.00 | -5.87 | **<.001** | 0.99 (0.99 ~ 0.99) |
| O2flow | 0.01 | 0.00 | 2.64 | **0.008** | 1.01 (1.01 ~ 1.02) |
| Totalco2 | 0.02 | 0.00 | 6.30 | **<.001** | 1.02 (1.02 ~ 1.03) |
| Dbp | -0.02 | 0.00 | -8.53 | **<.001** | 0.98 (0.97 ~ 0.98) |

Abbreviations:Apsiii, Acute Physiology and Chronic Health Evaluation;Bun,Blood Urea Nitrogen;Dbp,Diastolic Blood Pressure;Gcs,Glasgow Coma Scale;GCS Eye Opening;Inr.International Normalized Ratio;Mbp,Mean Blood Pressure;oasis,Open Source Anonymized Simulator;PH.Acidity;Pt,Prothrombin Time;Ppt,Partial Thromboplastin Time;Sapsii,Simplified Acute Physiology Score II；Sbp,Systolic Blood Pressure;SPo2,Peripheral Capillary Oxygen Saturation;

### Table S9. Comparison of AUC values among Internal validation model on the validation set.

| **Internal validation model** | | XGBOOST | RF | LR | DT | SVM | ANN |
| --- | --- | --- | --- | --- | --- | --- | --- |
|  |  | 0.816 | 0.81 | 0.802 | 0.734 | 0.806 | 0.759 |
| XGBOOST | 0.816 | NA | 0.2613 | 0.0005 | <0.001 | 0.0054 | <0.001 |
| RF | 0.81 | 0.2613 | NA | 0.0351 | <0.001 | 0.1049 | <0.001 |
| LR | 0.802 | 0.0005 | 0.0351 | NA | <0.001 | 0.501 | <0.001 |
| DT | 0.734 | <0.001 | <0.001 | <0.001 | NA | <0.001 | <0.001 |
| SVM | 0.806 | 0.0054 | 0.1049 | 0.501 | <0.001 | NA | <0.001 |
| ANN | 0.759 | <0.001 | <0.001 | <0.001 | <0.001 | <0.001 | NA |

Abbreviations:XGBOOST,Extreme Gradient Boosting;RF,Random Forest;LR,Logistic Regression;DT,Decision Tree;SVM,Support Vector Machine;ANN,Artificial Neural Network

### Table S10. Comparison of AUC values among External validation model on the validation set.

| **External validation model** | | XGBOOST | RF | LR | DT | SVM | ANN |
| --- | --- | --- | --- | --- | --- | --- | --- |
|  |  | 0.771 | 0.822 | 0.742 | 0.685 | 0.750 | 0.739 |
| XGBOOST | 0.771 | NA | <0.001 | <0.001 | <0.001 | 0.0003 | 0.0128 |
| RF | 0.822 | <0.001 | NA | <0.001 | <0.001 | <0.001 | <0.001 |
| LR | 0.742 | <0.001 | <0.001 | NA | <0.001 | 0.1443 | <0.001 |
| DT | 0.685 | <0.001 | <0.001 | <0.001 | NA | <0.001 | <0.001 |
| SVM | 0.750 | 0.0003 | <0.001 | 0.1443 | <0.001 | NA | 0.145 |
| ANN | 0.739 | 0.0128 | <0.001 | <0.001 | <0.001 | 0.145 | NA |

Abbreviations:XGBOOST,Extreme Gradient Boosting;RF,Random Forest;LR,Logistic Regression;DT,Decision Tree;SVM,Support Vector Machine;ANN,Artificial Neural Network

### Table S11 Comparison of AUC values of Internal validation model and External validation model

| **Internal validation model**  **External validation model** | | XGBOOST | RF | LR | DT | SVM | ANN |
| --- | --- | --- | --- | --- | --- | --- | --- |
|  |  | 0.771 | 0.822 | 0.742 | 0.685 | 0.750 | 0.739 |
| XGBOOST | 0.772 | 0.979 | NA | NA | NA | NA | NA |
| RF | 0.778 | NA | <0.001 | NA | NA | NA | NA |
| LR | 0.769 | NA | NA | 0.006 | NA | NA | NA |
| DT | 0.685 | NA | NA | NA | 0.952 | NA | NA |
| SVM | 0.778 | NA | NA | NA | NA | 0.006 | NA |
| ANN | 0.769 | NA | NA | NA | NA | NA | 0.244 |

Abbreviations:XGBOOST,Extreme Gradient Boosting;RF,Random Forest;LR,Logistic Regression;DT,Decision Tree;SVM,Support Vector Machine;ANN,Artificial Neural Network
